# Cochrane Collaboration Systematic Reviews may be based on trials not approved by a research ethics committee

**DOI:** 10.1002/cre2.79

**Published:** 2017-10-27

**Authors:** Asbjørn Jokstad

**Affiliations:** ^1^ Department of Clinical Dentistry, Faculty of Health Sciences UiT The Arctic University of Norway Norway

**Keywords:** biomedical research, research ethics committees, evidence‐based practice, oral health, policy

## Abstract

Systematic reviews (SR) may potentially contain reports of primary trials with ethical problems. The Cochrane Collaboration SRs are considered as the highest standard in evidence‐based health care resources. All SRs completed during the last 5 years (2013–2017) under the management of the Oral Health Group of the Cochrane Collaboration were identified. All primary trials included in the Oral Health Group SRs were identified and examined to establish their status regarding pre‐hoc approval of an independent ethics committee (EC), often termed Institutional Review Board (IRB) before commencing recruitment of trial participants. Ninety‐five SRs contained 960 primary trials, of which 272 (28.3%) were not examined by the author of this paper. Amongst the remaining 688 primary trials, 198 (29%) contained no reference to study conduct approval by a research ethics committee. The majority of primary studies referred to an EC/IRB approval with or without identifying the name of the ethics committee (n = 401, 58%), whereas some papers identified both the committee name and a protocol or reference number of the EC/IRB approval (n = 89, 13%). The Cochrane Collaboration, along with other developers of SRs, should adopt the policy established by COPE with regard to what to do if one suspect an ethical problem, that is, request evidence of EC/IRB approval. All stakeholders should rest assured that clinical policies and practices based on SRs are based on ethically sound clinical research.

## 1 INTRODUCTION

Some matters in evidence‐based medicine are taken for granted, such as assuming that the primary trials in an otherwise methodologically rigorous systematic review (SR) adhere to minimum research ethics standards. Developers of systematic reviews should consider whether or not a reported trial clarify that an independent human research ethics committee had approved the research protocol before commencing recruitment of trial participants. Regrettably, this appears often not to be considered in most SRs, at least in the oral and craniofacial research literature.

The Cochrane Collaboration SRs are internationally recognized as the highest standard in evidence‐based healthcare resources. Yet, the international Cochrane Collaboration provides little guidance on when to include or exclude an ethically dubious primary trial in a SR. Their training website discusses publishing ethics issues, such as authorship and contributorship, conflicts of interest, and libel and plagiarism (Cochrane Collaboration Training Website, 2017) but nothing about ethical aspects of the primary trials. Moreover, the pertinent section in their handbook on protection of human subjects and animals in research has been marked for several years with “We are working on this section” (Cochrane Collaboration Handbook, 2016a). The lack of focus raises the question whether Cochrane SRs may potentially contain reports of primary trials with ethical problems.

It is unknown how many of the existing Cochrane SRs that include trials without a statement about a pre‐hoc research ethics approval. It is also unknown whether the proportion of such trials varies amongst the different medical disciplines and sub‐disciplines. The objective of the current study was to establish the prevalence of primary trials in recent Cochrane SRs within the domains of dental and oral health that contain no explicit statement of a pre‐hoc ethics approval.

## 2 MATERIALS AND METHODS

The author identified all SRs completed during the last five years (2013–2017) under the management of the Oral Health Group (OHG) of the Cochrane Collaboration (OHG of the Cochrane Collaboration, 2017). The list of SRs was verified against the references identified in Medline by way of Pubmed using the search term “Cochrane Database Syst Rev”[jour] AND (dentistry OR dental OR “oral health”). The references were exported to EndNote X8.0.1 (Bld 1; Clarivate Analytics, New York, USA) and thereafter to a relational database (Microsoft Access 2016 [ver. 16.0.4229.1024]), run under Windows 10 operating system (Microsoft, Redmond, Washington, U.S.A.)

All primary trials that were included in the OHG SRs were identified, and these references were merged into the relational database. All primary trials that were accessible digitally were read in full to establish their status regarding pre‐hoc research ethics approval before commencing recruitment of trial participants. The terms “Institutional Research Board” (IRB) and “(Human) (Research) Ethics Committee” (EC) and any permutations thereof were considered as synonyms. Three categories of textual references to EC/IRB were identified; that is, either an EC/IRB was named with approval number and/or date or some reference was made to an EC/IRB, alternatively, there was no reference to any EC/IRB. Any referring to the Declaration of Helsinki was not considered equivalent to a formal EC/IRB approval. The extracted tabular data were not subjected to further statistical analyses.

## 3 RESULTS

Since January 2013, 98 SRs have been published or updated, of which the great majority focus on effects of an intervention (*n* = 95). Three SRs dealing with epidemiology of water fluoridation (Iheozor‐Ejiofor et al., 2015) and precision of diagnostics tests for cancer (Macey et al., 2015), (Walsh et al., 2013) were not considered further. The 95 SRs that were focused on effects of an intervention contained either no primary trials (*n* = 16 SRs) or presented extracted data from one (*n* = 13 SRs) up to 56 primary trials. Altogether, the 95 SRs contained 960 primary trials published between 1964 and 2016, of which 272 (28.3%) were not examined by the author of this paper. The reasons were predominantly due to no reading access (*n* = 115), paper was not available online (*n* = 88), non‐English language (*n* = 38), or for other reasons (abstract only, letter to the editor, and dissertation).

Amongst the 688 primary trials available digitally in English for full text reading, 198 (29%) contained no reference to any EC/IRB, whereas the majority referred to an EC/IRB (*n* = 401, 58%) and even included the protocol number of the EC/IRB approval (*n* = 89, 13%).

A marked change of the proportions of primary trials with and without a reference to an EC/IRB is apparent over time (Table 1). The first paper that describes an ethics approval in the identified pool of primary trials was published as late as 1989(Baab & Johnson, 1989). Since 2010, the vast majority of the primary trials describe that they were pre‐hoc approved by an EC/IRB.

**Table 1:** Description of approval by an ethics committee in the primary trials (*n* = 960) identified in all Cochrane Oral Health Group systematic reviews published since January 2013 (*n* = 95)

Period	1970←	1970–1979	1980–1984	1985–1989	1990–1994	1995–1999	2000–2004	2005–2009	2010→	All
Approved, with identifier	0	0	0	0	0	0	6	26	57	89
Approved	0	0	0	1	6	34	54	148	158	401
Not mentioned	4	6	7	13	13	29	44	44	38	198
Paper not examined	6	33	18	24	46	33	28	48	36	272
Sum	10	39	25	38	65	96	132	266	289	960
Proportions	%	%	%	%	%	%	%	%	%	%
Approved, with identifier	0.0	0.0	0.0	0.0	0.0	0.0	4.5	9.8	19.7	9.3
Approved	0.0	0.0	0.0	2.6	9.2	35.4	40.9	55.6	54.7	41.8
Not mentioned	40.0	15.4	28.0	34.2	20.0	30.2	33.3	16.5	13.1	20.6
Paper not examined	60.0	84.6	72.0	63.2	70.8	34.4	21.2	18.0	12.5	28.3

## 4 DISCUSSION

The question whether it is necessary that an EC/IRB needs to approve a human clinical trial before commencing recruitment of trial participants should today be a non‐issue. A requirement for a pre‐hoc approval by an IRB was introduced in 1974 in USA (National Research Act, 1974) and rapidly included as Article 2 in the first revision of the Declaration of Helsinki in October 1975(World Medical Association, 1975). Succeeding requirements were formulated in the Belmont report written by the National Commission for the Protection of Human Services of Biomedical and Behavioral Research in USA in 1977, and by the joint Council for International Organizations of Medical Sciences and the World Health Organization in 1982. The United Nations Educational, Scientific and Cultural Organization have even included the requirement under Article 19 in the 2005 Universal Declaration on Bioethics and Human Rights (United Nations Educational, Scientific and Cultural Organization, 2017). The publishing world has followed suit by a continuously amending and modifying minimum requirements for manuscript contents, as detailed by, for example, the World Association of Medical Editors, the International Committee of Medical Journal Editors and the Committee on Publication Ethics (COPE). The references above are an incomplete list of source documents. Claims have been made that it borders to research misconduct today to initiate a human trial without proper pre‐hoc EC/IRB approval before commencing recruitment of trial participants. There has been some considerations about how authors of Cochrane SRs may expose research misconduct, but issues about pre‐hoc EC/IRB approval has not been specifically mentioned (Vlassov & Groves, 2017).

Several of the current Cochrane SRs on oral and craniofacial medicine contain a disproportional fraction of primary trials with no statement about EC/IRB approval. Not only individual clinicians but also national and commercial health bodies develop clinical policies and practices that rely heavily on Cochrane SRs. Moreover, the Cochrane Collaboration is doing an excellent job promoting sensible guidance for lay people about the therapy effectiveness of different ailments and conditions (Cochrane Collaboration, 2016). In the opinion of the author, all readers need to be informed on whether the primary trials have been approved by an ethics committee. It is an ethical dilemma for all stakeholders to decide whether health recommendations should and can be founded on primary trials with possible questionable research ethics.

The Cochrane Collaboration has established clear guidelines for ethical considerations in their Editorial and Publishing Policy Resources and are extremely cognizant of potential risk of bias in trials. A recent survey amongst experts and stakeholders on how to improve the Cochrane Collaboration's tool for assessing the risk of bias is perhaps representative (Savović et al., 2014). Apart from the positive element that the survey had been pre‐hoc approved by the Ottawa Hospital Research Institute Ethics Committee, the investigators apparently did not solicit anyone's opinions about possible association between lack of EC/IRB approval and risk of potential bias. Perhaps this is just a reflection of the mentality of ethically cognizant researchers that some matters in clinical research are plainly obvious. This perception is reinforced by a quote from the Cochrane handbook in the section on risk of bias in nonrandomized studies: “Because of the need for research ethics approval, all randomized trials must have a protocol” (Cochrane Collaboration Handbook, 2016b).

The intention of this paper was not to single out particular factors associated with characteristics of the primary trials with regard to, for example, topic, the type of journal or the origin of the investigators. Rather, it was to focus on the need to base our clinical policies and practices on ethically sound primary trials. For the interested reader, further details about the EC/IRB status of the 960 primary trials may be sourced from the Supporting Information located on the website of Clinical and Experimental Dental Research (Clinical and Experimental Dental Research, 2017).

It is unknown whether the limited proportion of primary trials in compliance with proper publishing ethics is representative for Cochrane SRs within other medical domains. It seems sensible to conduct comparable analyses amongst other biomedical fields in the existing pool of Cochrane SRs.

The 11,578+ editors of journals that currently are members of COPE are expected to follow the *COPE Code of Conduct for Journal Editors* (*The COPE Code of Conduct for Journal Editors,* 2017), which includes assuring that research involving humans and animals is ethical. The practical guidance is that “editors should seek assurances that all research has been approved by an appropriate body where one exists”. It follows that journal editors have a primary duty to ensure that ethical approval is reported.

The lack of documented EC/IRB approval in a primary trial does not necessarily invalidate its conclusions, or by extension, conclusions in an SR based on aggregated data from such trials. The reason is that one cannot discount completely that the clinical investigators did indeed have an EC/IRB approval but forgot to add this information in the manuscript and both the journal referees and the editor simply had a bad day.

One solution to rectify the current predicament is that the Cochrane collaboration, along with other developers of SRs, adopts the policy established by COPE with regard to what to do if one suspects an ethical problem (Committee on Publication Ethics (COPE), 2017), that is, request documented evidence of EC/IRB approval. Moreover, one cannot rule out that conclusions in current SRs may potentially change if the primary trials without EC/IRB approval are disregarded. With regard to Cochrane SRs, the collaboration needs to decide on whether the editorial team of the Cochrane special groups should be responsible for verifying EC/IRB approvals in case of doubts, or if this should be left to the SR authors. The question may perhaps require some legal consultations due to potential unanticipated ramifications. Regardless, the essential issue is that all stakeholders may rest assured that clinical policies and practices based on Cochrane SRs are based on ethically sound clinical research.

## CONFLICT OF INTEREST

None declared.

## Supporting information

Data S1 Supporting information item
