# Supplementary material for: Cochrane Collaboration Systematic Reviews may be based on trials not approved by a research ethics committee
Source: Clin Exp Dent Res. 2017 Oct 27;3(5):179–82. doi: 10.1002/cre2.79 (PMC5839214; doi:10.1002/cre2.79)
Supplement: Supplementary file 1 — Data S1 Supporting information item [file CRE2-3-179-s001.pdf]

Cochrane Oral Health Group Systematic Reviews 2017.5 - 2013.1

Cochrane_Author | Primary studies_ | doi

Agnihotry A, Fedorowicz Z, Nasser M — http://doi.org/10.1002/14651858.CD007517.pub3

2016 Adhesively bonded versus non-bonded amalgam restorations for dental caries — Primary studies: 1

Year	Study	Reference
1999	Setcos 1999	Setcos JC, Staninec M, Wilson NH. A two-year randomized, controlled clinical evaluation of bonded amalgam restorations. Journal of Adhesive Dentistry 1999;1(4):323-31.
	quintpub.com/journals/jad/fulltext.php?article_id=9802	1 approved by a local ethics committee

Agnihotry A, Fedorowicz Z, van Zuuren EJ, Farman AG, Al-Langawi JH — http://doi.org/10.1002/14651858.CD004969.pub4

2016 Antibiotic use for irreversible pulpitis — Primary studies: 1

Year	Study	Reference
2000	Nagle 2000	Nagle D, Reader A, Beck M, Weaver J. Effect of systemic penicillin on pain in untreated irreversible pulpitis. Oral Surgery, Oral Medicine, Oral Pathology, Oral Radiology & Endodontics 2000;90(5):636-40.
	doi.org/10.1067%2Fmoe.2000.109777	1 approved by the Ohio State University Human Subjects Committee

Agostino P, Ugolini A, Signori A, Silvestrini-Biavati A, Harrison JE, Riley P — http://doi.org/10.1002/14651858.CD000979.pub2

2014 Orthodontic treatment for posterior crossbites — Primary studies: 15

Year	Study	Reference
2013	Lippold 2013	Lippold C, Stamm T, Meyer U, Végh A, Moiseenko T, Danesh G. Early treatment of posterior crossbite - a randomised clinical trial. Trials 2013;14:20.
	doi.org/10.1186%2F1745-6215-14-20	1 approved by the local Ethics Committee of the Medical Faculty, Wesphalian Wilhelms University, Münster (Germany)
2012	Martina 2012	Martina R, Cioffi I, Farella M, Leone P, Manzo P, Matarese G, et al. Transverse changes determined by rapid and slow maxillary expansion. A low-dose CT-based randomised controlled trial. Orthodontics & Craniofacial Research 2012;15:159-68.
	doi.org/10.1111/j.1601-6343.2012.01543.x	1 approved by the Institutional Review Board and the local Ethics Committee
2012	Oshagh 2012	Oshagh M, Momeni Danaei Sh, Hematiyan MR, Hajian Kh, Shokoohi Z. Comparison of dental arch changes and patients' discomforts between newly designed maxillary expansion screw and slow expansion procedures. Journal of Dentistry, Shiraz University of Medical Sciences 2012;13(3):110-9.
	dentjods.sums.ac.ir/index.php/JDSUMS/article/viewFile/43/[illegible]	1 approved by the ethical committee at the Shiraz University of Medical Sciences.
2011	Godoy 2011	Godoy F, Godoy-Bezerra J, Rosenblatt A. Treatment of posterior crossbite comparing 2 appliances: a community-based trial. American Journal of Orthodontics and Dentofacial Orthopedics 2011;139:e45-52.
	doi.org/10.1016/j.ajodo.2010.06.017	1 approved by the ethics committee of the University of Pernambuco

Year	Cochrane_Author	doi	Primary studies_	Ethics
2010	Lagravere 2010	doi.org/10.1016/j.ajodo.2009.10.004	Lagravere MO, Carey J, Heo G, Toogood RW, Major PW. Transverse, vertical, and anteroposterior changes from bone-anchored maxillary expansion vs traditional rapid maxillary expansion: a randomized clinical trial. American Journal of Orthodontics and Dentofacial Orthopedics 2010;137(3):304.	0 Ethics committee not mentioned
2010	Ramoglu 2010	doi.org/10.1093%2Fejo%2Fcjp057	Ramoglu SI, Sari Z. Maxillary expansion in the mixed dentition: rapid or semi-rapid?. European Journal of Orthodontics 2010;32:11-8.	0 Ethics committee not mentioned
2008	Kilic 2008	doi.org/10.1093/ejo/cjm099	Kilic N, Kiki A, Oktay H. A comparison of dentoalveolar inclination treated by two palatal expanders. European Journal of Orthodontics 2008;30(1):67-72.	0 Ethics committee not mentioned
2008	Petrén 2008	doi.org/10.1016%2Fj.ajodo.2007.11.021	Petrén S, Bondemark L. Correction of unilateral posterior crossbite in the mixed dentition: a randomized controlled trial. American Journal of Orthodontics and Dentofacial Orthopedics 2008;133:790.e7-13.	1 approved by the ethics committee of Lund University, Lund, Sweden
2005	Garib 2005	angle.org/doi/10.1043/0003-3219(2005)75[548:RMETVT]2.[illegible]	Garib DG, Henriques JF, Janson G, Freitas MR, Coelho RA. Rapid maxillary expansion - tooth tissue-borne versus tooth-borne expanders: a computed tomography evaluation of dentoskeletal effects. The Angle Orthodontist 2005;75(4):548-57.	1 approved by the Ethical Committee at Bauru School of Dentistry, University of Sao Paulo
2005	McNally 2005	doi.org/10.1179%2F146531205225020769	McNally MR, Spary DJ, Rock WP. Randomized controlled trial comparing the quadhelix and the expansion arch for the correction of crossbite. Journal of Orthodontics 2005;32(1):29-35.	1 approved by the Staffordshire Health Authority
2004	Oliveira 2004	doi.org/10.1016%2Fj.ajodo.2003.07.008	Oliveira NL, Da Silveira AC, Kusnoto B, Viana G. Three-dimensional assessment of morphologic changes of the maxilla: a comparison of 2 kinds of palatal expanders. American Journal of Orthodontics and Dentofacial Orthopedics 2004;126(3):354-62.	0 Ethics committee not mentioned
2003	Lamparski 2003	doi.org/10.1067%2Fmod.2003.10	Lamparski DG Jr, Rinchuse DJ, Close JM, Sciote JJ. Comparison of skeletal and dental changes between 2-point and 4-point rapid palatal expanders. American Journal of Orthodontics and Dentofacial Orthopedics 2003;123(3):321-8.	0 Ethics committee not mentioned
1997	Asanza 1997	angle.org/doi/10.1043/0003-3219(1997)067<0015:COHABE[illegible]	Asanza S. Comparison of Hyrax and bonded expansion appliances. The Angle Orthodontist 1997;67(1):15-22.	0 Ethics committee not mentioned

1989 Mossaz-Joelson Mossaz-Joelson K, Mossaz C. Slow maxillary expansion: a comparison between bonded and banded appliances. European Journal of Orthodontics 1989;11:67-76.

doi.org/10.1093%2Foxfordjournals.ejo.a035967

--- (article has not been accessed online) ---

1984 Thilander 1984 Thilander B, Wahlund S, Lennartsson B. The effect of early interceptive treatment in children with posterior cross-bite. European Journal of Orthodontics 1984;6:25-34.

doi.org/10.1093%2Fejo%2F6.1.25

--- (article has not been accessed online) ---

Ahangari Z, Nasser M, Mahdian M, Fedorowicz Z, Marchesan MA http://doi.org/10.1002/14651858.CD008003.pub3

2015 Interventions for the management of external root resorption Primary studies: 0

EMPTY Empty Systematic Review

Ahovuo-Saloranta A, Forss H, Hiiri A, Nordblad A, Makela M http://doi.org/10.1002/14651858.CD003067.pub4

2016 Pit and fissure sealants versus fluoride varnishes for preventing dental decay in the permanent teeth of children and ad Primary studies: 8

2014 Salem 2014 Salem K, Shaahsavari F, Kazemnejad E, Poorhabibi Z. Pit and fissure sealant versus fluoride varnish in prevention of occlusal caries. Journal of Dentomaxillofacial Radiology, Pathology and Surgery 2014;2(4):37-47.

3dj.gums.ac.ir/browse.php?a_id=93&slc_lang=en&sid=1&ftx[illegible]

2 approved by the Institutional Review Board and Ethical Committee of Guilan University of Medical Sciences, Rasht, IRAN, (6051 & 138802091861 IRCT)

2012 Liu 2012 Liu BY, Lo ECM, Chu CH, Lin HC. Randomized trial on fluorides and sealants for fissure caries prevention. Journal of Dental Research 2012;91(8):753-8.

doi.org/10.1177%2F0022034512452278

1 approved by the by the University of Hong Kong

2011 Tagliaferro 201 Tagliaferro EP, Pardi V, Ambrosano GM, Meneghim Mde C, da Silva SR, Pereira AC. Occlusal caries prevention in high and low risk schoolchildren. A clinical trial. American Journal of Dentistry 2011;24(2):109-14.

ncbi.nlm.nih.gov/pubmed/21698991

2 approved by the research ethics committee of the Piracicaba Dental School, UNICAMP, protocl # 025/2004

2007 Ji 2007 Ji PH, Xu QL, Ba Y. Clinical evaluation of fluor protector and glass-ionomer cement used as pit and fissure sealant for preventing pit and fissure caries in children. Shanghai Kou Qiang Yi Xue 2007;16(4):374-6.

ncbi.nlm.nih.gov/pubmed/17924020

--- (article has not been accessed online) ---

2005 Bravo 2005 Bravo M, Montero J, Bravo JJ, Baca P, Llodra JC. Sealant and fluoride varnish in caries: a randomized trial. Journal of Dental Research 2005;84(12):1138-43.

doi.org/10.1177%2F154405910508401209

1 approved by the University of Granada Faculty of Dentistry Ethics Committee

2001 Florio 2001
ncbi.nlm.nih.gov/pubmed/11985192
Florio FM, Pereira AC, Meneghim Mde C, Ramacciato JC. Evaluation of non-invasive treatment applied to occlusal surfaces. ASDC Journal of Dentistry for Children 2001;68(5-6):326-31, 301.
--- (article has not been accessed online) ---

2001 Splieth 2001
ejpd.eu/bibliografia_dettaglio.asp?id=74
Splieth C, Förster M, Meyer G. Additional caries protection by sealing permanent first molars compared to fluoride varnish applications in children with low caries prevalence: 2-year results. European Journal of Paediatric Dentistry 2001;2(3):133-8.
--- (article has not been accessed online) ---

1984 Raadal 1984
[doi.org/10.1111/j.1600-0528.1984.tb01414.x](doi.org/10.1111/j.1600-0528.1984.tb01414.x)
Raadal M, Laegreid O, Laegreid KV, Hveem H, Korsgaard EK, Wangen K. Fissure sealing of permanent first molars in children receiving a high standard of prophylactic care. Community Dentistry and Oral Epidemiology 1984;12(2):65-8.
--- (article has not been accessed online) ---

Albrecht M, Kupfer R, Reissmann DR, Muhlhauser I, Kopke S http://doi.org/10.1002/14651858.CD010535.pub2

2016 Oral health educational interventions for nursing home staff and residents Primary studies: 9

2013 Van der Putten
[doi.org/10.1007%2Fs00784-012-0793-2](doi.org/10.1007%2Fs00784-012-0793-2)
Van der Putten GJ, Mulder J, de Baat C, de Visschere L, Vanobbergen J, Schols J. Effectiveness of supervised implementation of an oral health care guideline in care homes; a single-blinded cluster randomized controlled trial. Clinical Oral Investigations 2013;17:1143-53.
2 approved by the Ethics Committee of Radboud University Nijmegen, the Netherlands (NL24666.091.08 approval 2008/273)

2013 Zenthöfer 2013
doi.org/10.1111/cdoe.12007
Zenthöfer A, Dieke R, Dieke A, Wege KC, Rammelsberg P, Hassel AJ. Improving oral hygiene in the long-term care of the elderly - a RCT. Community Dentistry and Oral Epidemiology 2013;41:261-8.
2 approved by the local university review board (number: 256/2004)

2012 De Visschere 20
[doi.org/10.1111/j.1741-2358.2010.00418.x](doi.org/10.1111/j.1741-2358.2010.00418.x)
De Visschere L, Schols J, Van der Putten GJ, De Baat C, Vanobbergen J. Effect evaluation of a supervised versus non-supervised implementation of an oral health care guideline in nursing homes: a cluster randomised controlled clinical trial. Gerodontology 2012;29:e96-106.
2 approved by the Ethics Committees of the Ghent University, Belgium (OG017 – approval 2008/440)

2011 De Visschere 20
[doi.org/10.1111/j.1600-0528.2011.00610.x](doi.org/10.1111/j.1600-0528.2011.00610.x)
De Visschere L, De Baat C, Schols JMGA, Deschepper E, Vanobbergen J. Evaluation of the implementation of an 'oral hygiene protocol' in nursing homes: a 5-year longitudinal study. Community Dentistry and Oral Epidemiology 2011;39:416-25.
2 approved by the Ethics Committee of the Gent University Hospital (OG017)

2007 MacEntee 2007
[doi.org/10.1111/j.1600-0528.2007.00318.x](doi.org/10.1111/j.1600-0528.2007.00318.x) [Indexed for MEDLINE]
MacEntee MI, Wyatt CCL, Beattie BL, Paterson B, Levy-Milne R, McCandless L, et al. Provision of mouth-care in long-term care facilities: an educational trial. Community Dentistry and Oral Epidemiology 2007;35:25-34.
1 approved by the Clinical Research Ethics Board

2005	Bellomo 2005	Bellomo F, De Preux F, Chung JP, Julien N, Budtz-Jørgensen E, Müller F. The advantages of occupational therapy in oral hygiene measures for institutionalised elderly adults. Gerodontology 2005;22:24-31.	
doi.org/10.1111/j.1741-2358.2004.00047.x		1 approved by the the local research ethics committee	
2001	Frenkel 2001	Frenkel HF, Harvey I, Needs KM. Oral health care education and its effect on caregivers' knowledge and attitudes: a randomised controlled trial. Community Dentistry and Oral Epidemiology 2002;30:91-100.	
doi.org/10.1034/j.1600-0528.2001.290408.x		1 approved by the Research Ethics Committee	
1998	Mojon 1998	Mojon P, Rentsch A, Budtz-Jørgensen E, Baehni PC. Effects of an oral health program on selected clinical parameters and salivary bacteria in a long-term care facility. European Journal of Oral Sciences 1998;106:827-34.	
doi.org/10.1046/j.0909-8836.1998.eos106401.x		--- (article has not been accessed online) ---	
1989	Schou 1989	Schou L, Wight C, Clemson N, Douglas S, Clark C. Oral health promotion for institutionalised elderly. Dental Health Education 1989;17:2-6.	
doi.org/10.1111/j.1600-0528.1985.tb00426.x		--- (article has not been accessed online) ---	

Ashley PF, Parekh S, Moles DR, Anand P, MacDonald LC http://doi.org/10.1002/14651858.CD008392.pub3

2016 Preoperative analgesics for additional pain relief in children and adolescents having dental treatment Primary studies: 5

2011	Baygin 2011	Baygin O, Tuzuner T, Isik B, Kusgoz A, Tanriver M. Comparison of pre-emptive ibuprofen, paracetamol, and placebo administration in reducing post-operative pain in primary tooth extraction. International Journal of Paediatric Dentistry 2011;21(4):306-13.	
doi.org/10.1111/j.1365-263X.2011.01124.x		1 approved by the Ethical Committee of Karadeniz Technical University, Faculty of Medicine	
2001	Bernhardt 2001	Bernhardt MK, Southard KA, Batterson KD, Logan HL, Baker KA, Jakobsen JR. The effect of pre-emptive and/or postoperative ibuprofen therapy for orthodontic pain. American Journal of Orthodontics and Dentofacial Orthopedics 2001;120(1):20-7.	
doi.org/10.1067%2Fmod.2001.115616		0 Ethics committee not mentioned	
2000	Law 2000	Steen Law SL, Southard KA, Law AS, Logan HL, Jakobsen JR. An evaluation of preoperative ibuprofen for treatment of pain associated with orthodontic separator placement. American Journal of Orthodontics and Dentofacial Orthopedics 2000;118(6):629-35.	
doi.org/10.1067%2Fmod.2000.110638		0 Ethics committee not mentioned	
1995	Primosch 1995	Primosch RE, Nichols DL, Courts FJ. Comparison of preoperative ibuprofen, acetaminophen, and placebo administration on parental report of postextraction pain in children. Pediatric Dentistry 1995;17(3):187-91.	
ncbi.nlm.nih.gov/pubmed/7617493		--- (article has not been accessed online) ---	

1993	Primosch 1993	Primosch R, Antony SJ, Courts FJ. The efficacy of preoperative analgesic administration for postoperative pain management of pediatric dental patients. Anesthesia and Pain Control in Dentistry 1993;2(2):102-6.
		--- (article has not been accessed online) ---

Ashley PF, Williams CE, Moles DR, Parry J http://doi.org/10.1002/14651858.CD006334.pub4

2015 Sedation versus general anaesthesia for provision of dental treatment to patients younger than 18 years Primary studies: 0

	EMPTY	Empty Systematic Review

Atieh MA, Alsabeeha NH, Payne AG, Duncan W, Faggion CM, Esposito M http://doi.org/10.1002/14651858.CD010176.pub2

2015 Interventions for replacing missing teeth: alveolar ridge preservation techniques for dental implant site development Primary studies: 8

2013	Festa 2013	Festa VM, Addabbo F, Laino L, Femiano F, Rullo R. Porcine-derived xenograft combined with a soft cortical membrane versus extraction alone for implant site development: a clinical study in humans. Clinical Implant Dentistry and Related Research 2013;15(5):707-13
doi.org/10.1111/j.1708-8208.2011.00398.x		1 approved by the local ethical committee
2013	Patel 2013	Patel K, Mardas N, Donos N. Radiographic and clinical outcomes of implants placed in ridge preserved sites: a 12-month post-loading follow-up. Clinical Oral Implants Research 2013;24(6):599-605
doi.org/10.1111/j.1600-0501.2012.02500.x		1 approved by the relevant independent committee on the Ethics of Human Research of University College London
2012	Barone 2012	Barone A, Orlando B, Cingano L, Marconcini S, Derchi G, Covani U. A randomised clinical trial to evaluate and compare implants placed in augmented versus non-augmented extraction sockets: 3-year results. Journal of Periodontology 2012;83(7):836-46
doi.org/10.1902%2Fjop.2011.110205		1 approved by the Ethics Committee of the Versilia General Hospital, Lido
2012	Brkovic 2012	Brkovic B, Prasad H, Rohrer M, Konandreas G, Agrogiannis G, Antunovic D, et al. Beta-tricalcium phosphate/type I collagen cones with or without a barrier membrane in human extraction socket healing: clinical, histologic, histomorphometric, and immunohistochemical evaluation. Clinical Oral Investigations 2012;16(2):581-90
doi.org/10.1007%2Fs00784-011-0531-1		2 approved by the Ethical Committee of the Faculty of Dentistry, University of Belgrade (No. 22/2,2006)
2012	Gholami 2012	Gholami GA, Najafi B, Mashhadiabbas F, Goetz W, Najafi S. Clinical, histologic and histomorphometric evaluation of socket preservation using a synthetic nanocrystalline hydroxyapatite in comparison with a bovine xenograft: a randomized clinical trial. Clinical Oral Implants Research 2012;23(10):1198-204
doi.org/10.1111/j.1600-0501.2011.02288.x		1 approved by the ethical committee of Shaheed Beheshti University of Medical Sciences
2012	Hoang 2012	Hoang TN, Mealey BL. Histologic comparison of healing after ridge preservation using human demineralized bone matrix putty with one versus two different-sized bone particles. Journal of Periodontology 2012;83(2):174-8
doi.org/10.1902%2Fjop.2011.110209		1 approved by the Institutional Review Board of the University of Texas Health Science Center at San Antonio (UTHSCSA), San Antonio, Texas

2011 Fernandes 2011 Fernandes PG, Novaes AB Jr, de Queiroz AC, de Souza SL, Taba M Jr, Palioto DB, et al. Ridge preservation with acellular dermal matrix and anorganic bone matrix cell-binding peptide P-15 after tooth extraction in humans. Journal of Periodontology 2011;82(1):72-9

doi.org/10.1902%2Fjop.2010.100241

2 approved by the Ethical Committee for Human Research of the same institution, protocol number 2009.1.388.58.0

2003 Iasella 2003 Iasella JM, Greenwell H, Miller RL, Hill M, Drisko C, Bohra AA, et al. Ridge preservation with freeze-dried bone allograft and a collagen membrane compared to extraction alone for implant site development: a clinical and histologic study in humans. Journal of Periodontology 2003;74(7):990-9.

doi.org/10.1902%2Fjop.2003.74.7.990

1 approved by the Human Studies Committee

Bailey E, Worthington HV, van Wijk A, Yates JM, Coulthard P, Afzal Z http://doi.org/10.1002/14651858.CD004624.pub2

2013 Ibuprofen and/or paracetamol (acetaminophen) for pain relief after surgical removal of lower wisdom teeth Primary studies: 7

2010 Mehlisch 2010 Mehlisch DR, Aspley S, Daniels SE, Bandy DP. Comparison of the analgesic efficacy of concurrent ibuprofen and paracetamol with ibuprofen or paracetamol alone in the management of moderate to severe acute postoperative dental pain in adolescents and adults: a randomized, double-blind, placebo-controlled, parallel-group, single-dose, two-center, modified factorial study. Clinical Therapeutics 2010;32(5):882-95.

doi.org/10.1016%2Fj.clinthera.2010.04.022

1 approved by the Coast Independent Review Board (Colorado Springs, Colorado)

2010 Mehlisch 2010a Mehlisch DR, Aspley S, Daniels SE, Southerden KA, Christensen KS. A single-tablet fixed-dose combination of racemic ibuprofen/paracetamol in the management of moderate to severe postoperative dental pain in adult and adolescent patients: a multicenter, two-stage, randomized, double-blind, parallel-group, placebo-controlled, factorial study. Clinical Therapeutics 2010;32(6):1033-49.

doi.org/10.1016%2Fj.clinthera.2010.06.002

1 approved by the Coast Independent Review Board (Colorado Springs, Colorado)

2009 Danliels 2009 Daniels S, Reader S, Berry P, Goulder M. Onset of analgesia with sodium ibuprofen, ibuprofen acid incorporating poloxamer and acetaminophen--a single-dose, double-blind, placebo-controlled study in patients with post-operative dental pain. European Journal of Clinical Pharmacology 2009;65(4):343-53.

doi.org/10.1007%2Fs00228-009-0614-y

1 approved by the Quorum Review Inc. (Seattle, WA)

2001 Olson 2001 Olson NZ, Otero AM, Marrero I, Tirado S, Cooper S, Doyle G, et al. Onset of analgesia for liquigel ibuprofen 400 mg, acetaminophen 1000 mg, ketoprofen 25 mg, and placebo in the treatment of postoperative dental pain. Journal of Clinical Pharmacology 2001;41(11):1238-47.

doi.org/10.1177/00912700122012797

1 approved by the appropriate institutional review board

2000 Hersch 2000 Hersh EV, Levin LM, Cooper SA, Doyle G, Waksman J, Wedell D, et al. Ibuprofen liquigel for oral surgery pain. Clinical Therapeutics 2000;22(11):1306-18.

doi.org/10.1016%2FS0149-2918%2800%2983027-1

1 approved by the University of Pennsylvania Committee on Studies Involving Human Beings

1995 Mehlisch 1995 Mehlisch DR, Jasper RD, Brown P, Korn SH, McCarroll K, Murakami AA. Comparative study of ibuprofen lysine and acetaminophen in patients with postoperative dental pain. Clinical Therapeutics 1995;17(5):852-60.

doi.org/10.1016%2F0149-2918%2895%2980063-8

1 approved by an institutional review board

1990 Forbes 1990
Forbes JA, Kehm CJ, Grodin CD, Beaver WT. Evaluation of ketorolac, ibuprofen, acetaminophen, and an acetaminophen-codeine combination in postoperative oral surgery pain. Pharmacotherapy 1990;10(6 (Pt 2)):94S-105S.
ncbi.nlm.nih.gov/pubmed/2082318
--- (article has not been accessed online) ---

Belmonte FM, Macedo CR, Day PF, Saconato H, Fernandes Moca Trevisani V http://doi.org/10.1002/14651858.CD006203.pub2
2013 Interventions for treating traumatised permanent front teeth: luxated (dislodged) teeth Primary studies: 0

EMPTY
Empty Systematic Review

Benson PE, Parkin N, Dyer F, Millett DT, Furness S, Germain P http://doi.org/10.1002/14651858.CD003809.pub3
2013 Fluorides for the prevention of early tooth decay (demineralised white lesions) during fixed brace treatment Primary studies: 3

2007 Stecksén-Blicks
Stecksén-Blicks C, Renfors G, Oscarson ND, Bergstrand F, Twetman S. Caries-preventive effectiveness of fluoride varnish: a randomized controlled trial in adolescents with fixed orthodontic appliances. Caries Research 2007;41(6):455-9.
doi.org/10.1159%2F000107932
1 approved by the local Ethics Committee of Umeå University

2006 Øgaard 2006
Øgaard B, Alm AA, Larsson E, Adolfsson U. A prospective, randomized clinical study on the effects of an amine fluoride/stannous fluoride toothpaste/mouthrinse on plaque, gingivitis and initial caries lesion development in orthodontic patients. European Journal of Orthodontics 2006;28(1):8-12.
doi.org/10.1093%2Fejo%2Fcji075
1 Approved by the ethics committee at the University of Gothenburg

2005 Luther 2005
Luther F, Tobin M, Robertson AJ, Toumba KJ. Fluoride-releasing glass beads in orthodontic treatment to reduce decay: a randomized, controlled clinical trial. World Journal of Orthodontics Supplement 2005;6(5):166-7.
--- (article has not been accessed online) ---

Borrie FR, Bearn DR, Innes NP, Iheozor-Ejiofor Z http://doi.org/10.1002/14651858.CD008694.pub2
2015 Interventions for the cessation of non-nutritive sucking habits in children Primary studies: 6

1997 Villa 1997
Villa NL, Cisneros GJ. Changes in the dentition secondary to palatal crib therapy in digit-suckers: a preliminary study. Pediatric Dentistry 1997;19(5):323-6.
ncbi.nlm.nih.gov/pubmed/9260225
--- (article has not been accessed online) ---

1990 Friman 1990
Friman PC, Leibowitz JM. An effective and acceptable treatment alternative for chronic thumb- and finger-sucking. Journal of Pediatric Psychology 1990;15(1):57-65.
doi.org/10.1093%2Fjpepsy%2F15.1.57
--- (article has not been accessed online) ---

1987	Christensen 198	doi.org/10.1111/j.1469-7610.1987.tb00211.x	Christensen AP, Sanders MR. Habit reversal and differential reinforcement of other behaviour in the treatment of thumb-sucking: an analysis of generalization and side-effects. Journal of Child Psychology and Psychiatry and Allied Disciplines 1987;28(2):281-95. 0 Ethics committee not mentioned
1980	Azrin 1980	doi.org/10.1016%2F0005-7967%2880%2990004-2	Azrin NH, Nunn R, Frantz-Renshaw S. Habit reversal treatment of thumbsucking. Behaviour Research and Therapy 1980;18:395-9. 0 Ethics committee not mentioned
1972	Larsson 1972	ncbi.nlm.nih.gov/pubmed/4517177	Larsson E. Dummy - and finger-sucking habits with special attention to their significance for facial growth and occlusion. Swedish Dental Journal 1972;65:1-5. --- (article has not been accessed online) ---
1967	Haryett 1967	doi.org/10.1016%2F0002-9416%2867%2990069-3	Haryett RD, Hansen FC, Davidson PO, Sandilands ML. Chronic thumb-sucking: The psychologic effects and the relative effectiveness of various methods of treatment. American Journal of Orthodontics 1967;53(8):569-85. 0 Ethics committee not mentioned

Brignardello-Petersen R, Carrasco-Labra A, Araya I, Yanine N, Cordova Jara L, http://doi.org/10.1002/14651858.CD010266.pub2

2015 Antibiotic prophylaxis for preventing infectious complications in orthognathic surgery Primary studies: 11

2011	Tan 2011	doi.org/10.1016%2Fj.ijom.2011.07.903	Tan SK, Lo J, Zwahlen RA. Are postoperative intravenous antibiotics necessary after bimaxillary orthognathic surgery? A prospective, randomized, double-blind, placebo-controlled clinical trial. International Journal of Oral and Maxillofacial Surgery 2011;40(12):1363-8. 1 approved by the local Human Ethics Committee
2010	Danda 2010	doi.org/10.1016%2Fj.joms.2009.09.081	Danda AK, Wahab A, Narayanan V, Siddareddi A. Single-dose versus single-day antibiotic prophylaxis for orthognathic surgery: a prospective, randomized, double-blind clinical study. Journal of Oral and Maxillofacial Surgery 2010;68(2):344-6. 0 Ethics committee not mentioned
2010	Samman 2010		Samman N, Cheung LK. Antibiotic prophylaxis for orthognathic surgery: a prospective trial of four penicillin regimes [abstract]. Journal of Craniomaxillofacial Surgery 1996;24(Suppl 1):100. --- (article has not been accessed online) ---
2009	Kang 2009	doi.org/10.3349%2Fymj.2009.50.1.55	Kang SH, Yoo JH, Yi CK. The efficacy of postoperative prophylactic antibiotics in orthognathic surgery: a prospective study in Le Fort I osteotomy and bilateral intraoral vertical ramus osteotomy. Yonsei Medical Journal 2009;50(1):55-9. 1 approved by the Institutional Review Board of the College of Dentistry, Yonsei University

2008 Jansisyanont 20
ncbi.nlm.nih.gov/pubmed/19127796
Jansisyanont P, Sessirisombat S, Sastravaha P, Bamroong P. Antibiotic prophylaxis for orthognathic surgery: a prospective, comparative, randomized study between amoxicillin-clavulanic acid and penicillin. Journal of the Medical Association of Thailand 2008;91(11):1726-31.
--- (article has not been accessed online) ---

2004 Baqain 2004
doi.org/10.1016%2Fj.bjoms.2004.06.010
Baqain ZH, Hyde N, Patrikidou A, Harris M. Antibiotic prophylaxis for orthognathic surgery: a prospective, randomised clinical trial. British Journal of Oral and Maxillofacial Surgery 2004;42(6):506-10.
1 approved by the Ethical Committee

2003 Lindeboom 200
doi.org/10.1067%2Fmoe.2003.54
Lindeboom JA, Baas EM, Kroon FH. Prophylactic single-dose administration of 600 mg clindamycin versus 4-time administration of 600 mg clindamycin in orthognathic surgery: a prospective randomized study in bilateral mandibular sagittal ramus osteotomies. Oral Surgery, Oral Medicine, Oral Pathology and Radiology and Endodontics 2003:95(2):145-9.
1 approved by the Medical Ethical Committee of the Medical Center of Amsterdam,

1999 Bentley 1999
ncbi.nlm.nih.gov/pubmed/10077192
Bentley KC, Head TW, Aiello GA. Antibiotic prophylaxis in orthognathic surgery: a 1-day versus 5-day regimen. Journal of Orthopaedic Trauma 1999;57(3):226-30.
0 Ethics committee not mentioned

1999 Zijderveld 1999
[doi.org/10.1016%2FS0278-2391%2899%2990718-8](doi.org/10.1016%2FS0278-2391%2899%2990718-8)
Zijderveld SA, Smeele LE. Preoperative antibiotic prophylaxis in orthognathic surgery: a randomised double-blind and placebo-controlled clinical trial. J Oral Maxillofac Surg. 1999 Dec;57(12):1403-6
1 approved by the local Ethics Committee

1994 Fridrich 1994
ncbi.nlm.nih.gov/pubmed/7989814
Fridrich KL, Partnoy BE, Zeitler DL. Prospective analysis of antibiotic prophylaxis for orthognathic surgery. International Journal of Adult Orthodontics and Orthodontic Surgery 1994;9(2):129-31.
--- (article has not been accessed online) ---

1984 Ruggles 1984
[doi.org/10.1016%2F0278-2391%2884%2990348-3](doi.org/10.1016%2F0278-2391%2884%2990348-3)
Ruggles JE, Hann JR. Antibiotic prophylaxis in intraoral orthognathic surgery. Journal of Oral and Maxillofacial Surgery 1984;42(12):797-801.
0 Ethics committee not mentioned

Brocklehurst P, Kujan O, O'Malley LA, Ogden G, Shepherd S, Glenny AM http://doi.org/10.1002/14651858.CD004150.pub4
2013 Screening programmes for the early detection and prevention of oral cancer Primary studies: 1

2000 Sankaranarayan
[doi.org/10.1016%2FS1368-8375%2803%2900041-1](doi.org/10.1016%2FS1368-8375%2803%2900041-1)
Sankaranarayanan R, Mathew B, Jacob BJ, Thomas G, Somanathan T, Pisani P, et al. Early findings from a community-based, cluster-randomized, controlled oral cancer screening trial in Kerala, India. Cancer 2000;88(3):664-73.
1 approved by the institutional ethical committee

Carvalho FR, Lentini-Oliveira DA, Prado LB, Prado GF, Carvalho LB http://doi.org/10.1002/14651858.CD005520.pub3
2016 Oral appliances and functional orthopaedic appliances for obstructive sleep apnoea in children Primary studies: 1

2002 Villa 2002 Villa MP, Bernkopf E, Pagani J, Broia V, Montesano M, Ronchetti R. Randomized controlled study of an oral jaw-positioning appliance for the treatment of obstructive sleep apnea in children with malocclusion. American Journal of Respiratory and Critical Care Medicine 2002;165(1):123-7

doi.org/10.1164%2Fajrccm.165.1.2011031

1 approved by the local ethics committee

Chan KK, Glenny AM, Weldon JC, Furness S, Worthington HV, Wakeford H http://doi.org/10.1002/14651858.CD010341.pub2

2015 Interventions for the treatment of oral and oropharyngeal cancers: targeted therapy and immunotherapy Primary studies: 12

2014 Ang 2014 Ang KK, Zhang QE, Rosenthal DI, Nguyen-Tan P, Sherman EJ, Weber RS, et al. A randomized phase III trial (RTOG 0522) of concurrent accelerated radiation plus cisplatin with or without cetuximab for stage III-IV head and neck squamous cell carcinomas (HNC). Journal of Clinical Oncology 2014;32(27):2940-50.

doi.org/10.1200%2FJCO.2013.53.5633

1 approved by the central and institutional review boards of the 151 participating centers

2014 Reddy 2014 Reddy BK, Lokesh V, Vidyasagar MS, Shenoy K, Babu KG, Shenoy A, et al. Nimotuzumab provides survival benefit to patients with inoperable advanced squamous cell carcinoma of the head and neck: a randomized, open-label, phase IIb, 5-year study in Indian patients. Oral Oncology 2014;50(5):498-505.

doi.org/10.1016%2Fj.oraloncology.2013.11.008

2 approved by the respective institutional review boards or ethical committees (Trial registry: h-R3/SCCHN/001/IND)

2013 Harrington 201 Harrington K, Berrier A, Robinson M, Remenar E, Housset M, de Mendoza FH, et al. Randomised Phase II study of oral lapatinib combined with chemoradiotherapy in patients with advanced squamous cell carcinoma of the head and neck: rationale for future randomised trials in human papilloma virus-negative disease. European Journal of Cancer 2013: Vol. 49. issue 7:1609-18

doi.org/10.1016/j.ejca.2012.11.023

1 approved by the institutional review board or independent ethics committee at each clinical site

2013 Koh 2013 Koh Y, Lee KW, Kim SB, Park KH, Shin SW, Kang JH, et al. A randomised multicentre open phase 2 study of cetuximab with docetaxel, cisplatin as induction chemotherapy in unresectable locally advanced head and neck squamous cell cancer. Journal of Clinical Oncology 2013;31:Abstract 6069.

doi.org/10.1634/theoncologist.2015-0208

--- (article has not been accessed online) ---

2013 Martins 2013 Martins RG, Parvathaneni U, Bauman JE, Sharma AK, Raez LE, Papagikos MA, et al. Cisplatin and radiotherapy with or without erlotinib in locally advanced squamous cell carcinoma of the head and neck: a randomized phase II trial. Journal of Clinical Oncology 2013; Vol. 31, issue 11:1415-21.

doi.org/10.1200/JCO.2012.46.3299

0 Ethics committee not mentioned

2013 Singh 2013 Singh KR, Dixit AK, Prashad SN, Saxena T, Shahoo DP, Sharma D. A randomized trial comparing radiotherapy alone versus radiotherapy with geftinib in locally advance oral cavity cancer. Clinical Cancer Investigation Journal 2013;2:29-33.

doi.org/10.4103%2F2278-0513.110768

0 Ethics committee not mentioned

2011 Gregoire 2011 Gregoire V, Hamoir M, Chen C, Kane M, Kawecki A, Julka PK, et al. Gefitinib plus cisplatin and radiotherapy in previously untreated head and neck squamous cell carcinoma: a phase II, randomized, double-blind, placebo-controlled study. Radiotherapy and Oncology: Journal of the European Society for Therapeutic Radiology and Oncology 2011; Vol. 100. issue 1:62-9.

sciencedirect.com/science/article/pii/S0167814011003847

1 approved by the the Institutional Review Board or Independent Ethics Committee at participating centers

2010	Rodriguez 2010	Rodriguez MO, Rivero TC, Bahi RDC, Muchuli CR, Bilbao MA, Vinageras EN, et al. Nimotuzumab plus radiotherapy for unresectable squamous-cell carcinoma of the head and neck. Cancer Biology and Therapy 2010; Vol. 9, issue 5:343-9.	
tandfonline.com/doi/pdf/10.4161/cbt.9.5.10981		1	approved by the Institutional Review Boards of all research sites as well as by the National Regulatory Authority
2006	Bonner 2006	Bonner JA, Harari PM, Giralt J, Azarnia N, Shin DM, Cohen RB, et al. Radiotherapy plus cetuximab for squamous-cell carcinoma of the head and neck. New England Journal of Medicine 2006; Vol. 354, issue 6:567-78.	
nejm.org/doi/full/10.1056/nejmoa053422 - t=article		1	approved by the ethics review boards at the participating institution
2002	De Stefani 2002	De Stefani A, Forni G, Ragona R, Cavallo G, Bussi M, Usai A, et al. Improved survival with perilymphatic interleukin 2 in patients with resectable squamous cell carcinoma of the oral cavity and oropharynx. Cancer 2002; Vol. 95, issue 1:90-7.	
doi.org/10.1002/cncr.10654		1	approved by the Italian Ministry of Health and the Ethics Committee of the Turin University St. John the Baptist Greater Hospital
1998	Mantovani 199	Mantovani G, Gebbia V, Airoldi M, Bumma C, Contu P, Bianchi A, et al. Neo-adjuvant chemo-(immuno-)therapy of advanced squamous-cell head and neck carcinoma: a multicenter, phase III, randomized study comparing cisplatin + 5-fluorouracil (5-FU) with cisplatin + 5-FU + recombinant interleukin 2. Cancer Immunology, Immunotherapy 1998; Vol. 47, issue 3:149-56.	
		1	approved by the Ethics Committee of the Department of Internal Medicine, University of Cagliari
1981	Bier 1981	Bier J, Rapp HJ, Borsos T. Randomized clinical study on intratumoral BCG-cell wall preparation (CWP) therapy in patients with squamous cell carcinoma in the head and neck region. Cancer Immunology, Immunotherapy 1981; Vol. 12, issue 1:71-9.	
		0	Ethics committee not mentioned

Chong LY, Clarkson JE, Dobbyn-Ross L, Bhakta S http://doi.org/10.1002/14651858.CD005101.pub3

2014 Slow-release fluoride devices for the control of dental decay Primary studies: 1

2005	Toumba 2005	Toumba KJ, Curzon ME. A clinical trial of a slow-releasing fluoride device in children. Caries Research 2005;39(3):195-200. [DOI:10.1159/000084798]	
doi.org/10.1159%2F000084798		1	approved by the Research Ethics Committee of the Leeds General Infirmary

Cooper AM, O'Malley LA, Elison SN, Armstrong R, Burnside G, Adair P, Dugdill http://doi.org/10.1002/14651858.CD009378.pub2

2013 Primary school-based behavioural interventions for preventing caries Primary studies: 4

2009	Saied-Moallemi	Saied-Moallemi Z, Virtanen JI, Vehkalahti MM, Tehranchi A, Murtomaa H. School-based intervention to promote preadolescents' gingival health: a community trial. Community Dentistry and Oral Epidemiology 2009;37(6):518-26.	
doi.org/10.1111/j.1600-0528.2009.00491.x		1	approved by the Ethics Committee of Shaheed Beheshti University, Tehran, Iran
2007	Zanin 2007	Zanin L, Meneghim MC, Assaf AV, Cortellazzi KL, Pereira AC. Evaluation of an educational program for children with high risk of caries. Journal of Clinical Pediatric Dentistry 2007;31(4):246-50.	
doi.org/10.17796%2Fjcpd.31.4.e521842t26200h27		1	approved by the Ethical Committee in Research at Piracicaba Dentistry Schoo/UNICAMP (State University of Campinas)

2001 Worthington 20 Worthington HV, Hill KB, Mooney J, Hamilton FA, Blinkhorn AS. A cluster randomized controlled trial of a dental health education program for 10-year-old children. Journal of Public Health Dentistry 2001;61(1):22-7.
doi.org/10.1111/j.1752-7325.2001.tb03351.x
0 Ethics committee not mentioned

1994 Petrecca 1994 Petrecca S, D'Arcangelo C, Esposito P, Di Marco N. A 2-year evaluation of different methods of dental caries prophylaxis in a sample school-aged population. Minerva Stomatologica 1994;43(1-2):23-8.
--- (article has not been accessed online) ---

Cope A, Francis N, Wood F, Mann MK, Chestnutt IG http://doi.org/10.1002/14651858.CD010136.pub2
2014 Systemic antibiotics for symptomatic apical periodontitis and acute apical abscess in adults Primary studies: 2

2001 Henry 2001 Henry M, Reader A, Beck M. Effect of penicillin on postoperative endodontic pain and swelling in symptomatic necrotic teeth. Journal of Endodontics 2001;27(2):117-23.
doi.org/10.1097%2F00004770-200102000-00016
1 approved by the Ohio State University Human Subjects Committee

1996 Fouad 1996 Fouad AF, Rivera EM, Walton RE. Penicillin as a supplement in resolving the localized acute apical abscess. Oral Surgery, Oral Medicine, Oral Pathology, Oral Radiology, and Endodontology 1996;81(5):590-5.
doi.org/10.1016%2FS1079-2104%2896%2980054-0
0 Ethics committee not mentioned

Coulthard P, Bailey E, Esposito M, Furness S, Renton TF, Worthington HV http://doi.org/10.1002/14651858.CD004345.pub2
2014 Surgical techniques for the removal of mandibular wisdom teeth Primary studies: 35

2013 Rullo 2013 Rullo R, Addabbo F, Papaccio G, D'Aquino R, Festa VM. Piezoelectric device vs. conventional rotative instruments in impacted third molar surgery: relationships between surgical difficulty and postoperative pain with histological evaluations. Journal of Cranio-Maxillo-Facial Surgery 2013;41(2):e33-8
doi.org/10.1016%2Fj.jcms.2012.07.007
1 approved by the local ethical committee

2012 Baqain 2012 Baqain ZH, Al-Shafii A, Hamdan AA, Sawair FA. Flap design and mandibular third molar surgery: A split mouth randomized clinical study. International Journal of Oral and Maxillofacial Surgery 2012; Vol. 41, issue 8:1020-4
doi.org/10.1016/j.ijom.2012.02.011
2 approved by the JUH Research Board (protocol no 155/2010).

2012 Goldsmith 2012 Goldsmith SM, De Silva RK, Tong DC, Love RM. Influence of a pedicle flap design on acute postoperative sequelae after lower third molar removal. International Journal of Oral and Maxillofacial Surgery 2012;41(3):371-5.
doi.org/10.1016%2Fj.ijom.2011.08.006
1 approved by the Health and Disability Commissioner Lower South Island ethics committee and Ngai Tahu Research consultation committee

2012 Hashemi 2012 Hashemi HM, Beshkar M, Aghajani R. The effect of sutureless wound closure on postoperative pain and swelling after impacted mandibular third molar surgery. British Journal of Oral and Maxillofacial Surgery 2012;50(3):256-8.
doi.org/10.1016%2Fj.bjoms.2011.04.075
1 approved by the ethics committee of Tehran University of Medical Sciences

2012	Osunde 2012	Osunde OD, Adebola RA, Saheeb BD. A comparative study of the effect of suture-less and multiple suture techniques on inflammatory complications following third molar surgery. International Journal of Oral and Maxillofacial Surgery 2012; Vol. 41, issue 10:1275-9.	doi.org/10.1016/j.ijom.2012.04.009
		1 approved by the Ethics Committee of the Aminu Kano Teaching Hospital.	
2011	Bello 2011	Bello SA, Olaitan AA, Ladeinde AL. A randomized comparison of the effect of partial and total wound closure techniques on postoperative morbidity after mandibular third molar surgery. Journal of Oral and Maxillofacial Surgery 2011;69:e24-30.	doi.org/10.1016%2Fj.joms.2011.01.025
		1 approved by the ethics and privileges committee of the hospital	
2011	Briguglio 2011	Briguglio F, Zenobio EG, Isola G, Briguglio R, Briguglio E, Farronato D, et al. Complications in surgical removal of impacted mandibular third molars in relation to flap design: clinical and statistical evaluations. Quintessence International 2011; Vol. 42, issue 6:445-53.	quintpub.com/journals/qi/fulltext.php?article_id=10727
		1 approved by the local ethics committee (PUC Minas)	
2011	Erdogan 2011	Erdogan O, Tatli U, Ustun Y, Damlar I. Influence of two different flap designs on the sequelae of mandibular third molar surgery. Oral and Maxillofacial Surgery 2011;15(3):147-52.	doi.org/10.1007%2Fs10006-011-0268-7
		0 Ethics committee not mentioned	
2011	Osunde 2011	Osunde OD, Saheeb BD, Adebola RA. Comparative study of effect of single and multiple suture techniques on inflammatory complications after third molar surgery. Journal of Oral and Maxillofacial Surgery 2011;69:971-6.	doi.org/10.1016%2Fj.joms.2010.05.009
		1 approved by the research and ethics committee of the Aminu Kano Teaching Hospital	
2011	Refo'a 2011	Refo'a Y, Ouatik N, Golchin F, Mahboobi N. Comparing primary and secondary wound healing discomfort after mandibular third molar surgery: a randomized, double-blind clinical trial. General Dentistry 2011; Vol. 59, issue 4:310-3.	ncbi.nlm.nih.gov/pubmed/21903571
		1 approved by the research ethics committee of Tehran University for Medical Sciences	
2010	Barone 2010	Barone A, Marconcini S, Giacomelli L, Rispoli L, Calvo JL, Covani U. A randomized clinical evaluation of ultrasound bone surgery versus traditional rotary instruments in lower third molar extraction. Journal of Oral and Maxillofacial Surgery 2010; Vol. 68, issue 2:330-6.	doi.org/10.1016/j.joms.2009.03.053
		0 Ethics committee not mentioned	
2010	Danda 2010	Danda AK, Krishna Tatiparthi M, Narayanan V, Siddareddi A. Influence of primary and secondary closure of surgical wound after impacted mandibular third molar removal on postoperative pain and swelling--a comparative and split mouth study. Journal of Oral and Maxillofacial Surgery 2010;68(2):309-12.	doi.org/10.1016%2Fj.joms.2009.04.060
		1 approved by the ethics committee of Saveetha University	
2010	Haraji 2010	Haraji A, Motamedi MH, Rezvani F. Can flap design influence the incidence of alveolar osteitis following removal of impacted mandibular third molars?. General Dentistry 2010; Vol. 58, issue 5:e187-9.	ncbi.nlm.nih.gov/pubmed/20829150
		0 Ethics committee not mentioned	

Cochrane_Author		Primary studies_	doi
2010	Roode 2010	Roode GJ, Butow K. An alternative surgical flap design for impacted third molars: a comparison of two different surgical techniques. SADJ 2010;65(6):246, 248-51.	
ncbi.nlm.nih.gov/pubmed/20879647		--- (article has not been accessed online) ---	
2010	Sandhu 2010	Sandhu A, Sandhu S, Kaur T. Comparison of two different flap designs in the surgical removal of bilateral impacted mandibular third molars. International Journal of Oral and Maxillofacial Surgery 2010;39(11):1091-6.	
doi.org/10.1016%2Fj.ijom.2010.07.003		0 Ethics committee not mentioned	
2009	Leung 2009	Leung YY, Cheung LK. Safety of coronectomy versus excision of wisdom teeth: a randomized controlled trial. Oral Surgery Oral Medicine Oral Pathology Oral Radiology Endodontics 2009; Vol. 108, issue 6:821-7.	
doi.org/10.1016/j.tripleo.2009.07.004		1 approved by the Institutional Review Board of The University of Hong Kong/Hospital Authority Hong Kong West Cluster	
2008	Chukwuneke 20	Chukwuneke FN, Oji C, Saheeb DB. A comparative study of the effect of using a rubber drain on postoperative discomfort following lower third molar surgery. International Journal of Oral and Maxillofacial Surgery 2008;37(4):341-4.	
doi.org/10.1016%2Fj.ijom.2007.11.016		1 approved by the ethics committee of the University of Nigeria Teaching Hospital, Enug	
2008	Xavier 2008	Xavier RL, Vasconcelos BC, Caubi AF, Porto GG, Maurette MA. Passive drainage through the vestibular oblique incision in impacted inferior third molar surgery: a preliminary study. Acta Odontologica Latinoamericana 2008; Vol. 21, issue 1:57-63.	
		--- (article has not been accessed online) ---	
2007	Kirk 2007	Kirk DG, Liston PN, Tong DC, Love RM. Influence of two different flap designs on incidence of pain,swelling, trismus, and alveolar osteitis in the week following third molar surgery. Oral Surgery, Oral Medicine, Oral Pathology, Oral Radiology and Endodontics 2007;104(1):e1-6.	
doi.org/10.1016%2Fj.tripleo.2007.01.032		0 Ethics committee not mentioned	
2007	Praveen 2007	Praveen G, Rajesh P, Neelakandan RS, Nandagopal CM. Comparison of morbidity following the removal of mandibular third molar by lingual split, surgical bur and simplified split bone technique. Indian Journal of Dental Research 2007;18(1):15-8.	
doi.org/10.4103%2F0970-9290.30916		--- (article has not been accessed online) ---	
2006	Srinivas 2006	Srinivas DR. Effect of Surgical Tube Drain with Primary Closure Technique after Removal of Impacted Mandibular Third Molars - a Clinical Study (Dissertation). Kamataka, Bangalore: Rajiv Gandhi University of Health Sciences. Karnataka, Bangalore, India: Rajiv Gandhi University of Health Sciences, 2006:1-51.	
		--- (article has not been accessed online) ---	
2005	Gomes 2005	Gomes ACA, Vasconcelos BC, de Oliveira e Silva ED, da Silva LC. Lingual nerve damage after mandibular third molar surgery: a randomised clinical trial. Journal of Oral and Maxillofacial Surgery 2005;63(10):1443-6.	
doi.org/10.1016%2Fj.joms.2005.06.012		0 Ethics committee not mentioned	

Year	Cochrane_Author	Primary studies_
2005	Pasqualini 2005	Pasqualini D, Cocero N, Castella A, Mela L, Bracco P. Primary and secondary closure of the surgical wound after removal of impacted mandibular third molars: a comparative study. International Journal of Oral and Maxillofacial Surgery 2005;34(1):52-7.
doi.org/10.1016%2Fj.ijom.2004.01.023		0 Ethics committee not mentioned
2005	Renton 2005	Renton T, Hankins M, Sproate C, McGurk M. A randomised controlled clinical trial to compare the incidence of injury to the inferior alveolar nerve as a result of coronectomy and removal of mandibular third molars. British Journal of Oral and Maxillofacial Surgery 2005;43(1):7-12.
doi.org/10.1016%2Fj.bjoms.2004.09.002		1 approved by the local ethics committee
2004	Cerqueira 2004	Cerqueira PR, Vasconcelos BC, Bessa-Nogueira RV. Comparative study of the effect of a tube drain in impacted lower third molar surgery. Journal of Oral and Maxillofacial Surgery 2004;62(1):57-61.
doi.org/10.1016%2FS0278-2391%2803%2900675-X		0 Ethics committee not mentioned
2003	Saglam 2003	Saglam AA. Effects of tube drain with primary closure techniques on postoperative trismus and swelling after removal of fully impacted mandibular third molars. Quintessence International 2003;34(2):143-7.
quintpub.com/journals/qi/fulltext.php?article_id=4859		0 Ethics committee not mentioned
2002	Nageshwar 200	Nageshwar N. Comma incision for impacted mandibular third molars. Journal of Oral and Maxillofacial Surgery 2002;60(12):1506-9.
doi.org/10.1053%2Fjoms.2002.36152		0 Ethics committee not mentioned
2000	Gargallo-Albiol	Gargallo-Albiol J, Buenechea-Imaz R, Gay-Escoda C. Lingual nerve protection during surgical removal of lower third molars. A prospective randomised study. International Journal Oral and Maxillofacial Surgery 2000;29(4):268-71.
doi.org/10.1016%2FS0901-5027%2800%2980026-0		0 Ethics committee not mentioned
1997	Rakprasitkul 19	Rakprasitkul S, Pairuchvej V. Mandibular third molar surgery with primary closure with a drain. International Journal of Oral and Maxillofacial Surgery 1997;26(3):187-90.
doi.org/10.1016%2FS0901-5027%2897%2980817-X		0 Ethics committee not mentioned
1996	Mocan 1996	Mocan A, Kisnisci R, Ucok C. Stereophotogrammetric and clinical evaluation of morbidity after removal of lower third molars by two different surgical techniques. Journal of Oral and Maxillofacial Surgery 1996;54(2):171-5.
doi.org/10.1016%2FS0278-2391%2896%2990441-3		0 Ethics committee not mentioned
1994	Greenwood 199	Greenwood M, Langton SG, Rood JP. A comparison of broad and narrow retractors for lingual nerve protection during lower third molar surgery. British Journal of Oral and Maxillofacial Surgery 1994;32(2):114-7.
doi.org/10.1016%2F0266-4356%2894%2990142-2		0 Ethics committee not mentioned

1993	Absi 1993	Absi EG, Shepherd JP. A comparison of morbidity following the removal of lower third molars by lingual split and surgical bur methods. International Journal of Oral and Maxillofacial Surgery 1993;22(3):149-53.	
doi.org/10.1016%2FS0901-5027%2805%2980240-1		0	Ethics committee not mentioned
1988	de Brabander 1	de Brabander EC, Cattaneo G. The effect of surgical drain together with a secondary closure technique on postoperative trismus, swelling and pain after mandibular third molar surgery. International Journal of Oral and Maxillofacial Surgery 1998;17(2):119-21.	
doi.org/10.1016%2FS0901-5027%2888%2980164-4		0	Ethics committee not mentioned
1977	Butler 1977	Butler DP, Sweet JB. Effect of lavage on the incidence of localized osteitis in mandibular third molar extraction sites. Oral Surgery 1977;44(1):14-20.	
doi.org/10.1016%2F0030-4220%2877%2990235-3		0	Ethics committee not mentioned
1976	Sweet 1976	Sweet JB, Butler MS, Drager JL. Effects of lavage techniques with third molar surgery. Oral Surgery, Oral Medicine, and Oral Pathology 1976;41(2):152-68.	
doi.org/10.1016%2F0030-4220%2876%2990226-7		0	Ethics committee not mentioned

Coulthard P, Kushnerev E, Yates JM, Walsh T, Patel N, Bailey E, Renton TF http://doi.org/10.1002/14651858.CD005293.pub2

2014 Interventions for iatrogenic inferior alveolar and lingual nerve injury Primary studies: 2

1996	Khullar 1996a	Khullar SM, Brodin P, Barkvoll P, Haanæs HR. Preliminary study of low-level laser for treatment of long-standing sensory aberrations in the inferior alveolar nerve. Journal of Oral and Maxillofacial Surgery 1996;54(1):2-7.	
doi.org/10.1016%2FS0278-2391%2896%2990290-6		1	approved by the Research Committee, Health Region II, Norway
1996	Khullar 1996b	Khullar SM, Emami B, Westermark A, Haanæs HR. Effect of low-level laser treatment on neurosensory deficits subsequent to sagittal split ramus osteotomy. Oral Surgery, Oral Medicine, Oral Pathology, Oral Radiology and Endodontics 1996;82(2):132-8.	
doi.org/10.1016%2FS1079-2104%2896%2980215-0		1	approved by the Ethical Committee of the Karolinska Hospital.

Dashash M, Yeung CA, Jamous I, Blinkhorn A http://doi.org/10.1002/14651858.CD007157.pub2

2013 Interventions for the restorative care of amelogenesis imperfecta in children and adolescents Primary studies: 0

	EMPTY	Empty Systematic Review

de Souza RF, Travess H, Newton T, Marchesan MA http://doi.org/10.1002/14651858.CD007820.pub3

2015 Interventions for treating traumatised ankylosed permanent front teeth Primary studies: 0

Cochrane_Author	Primary studies_	doi
EMPTY	Empty Systematic Review	

Del Fabbro M, Corbella S, Sequeira-Byron P, Tsesis I, Rosen E, Lolato A, Taschi http://doi.org/10.1002/14651858.CD005511.pub3

2016 Endodontic procedures for retreatment of periapical lesions Primary studies: 20

Year	Study	Reference
2015	Angerame 2015<br>doi.org/10.1016%2Fj.gien.2015.08.003	Angerame D, De Biasi M, Kastrioti I, Franco V, Castaldo A, Maglione M. Application of platelet-rich fibrin in endodontic surgery: a pilot study. Giornale Italiano di Endodonzia 2015;29:51-7<br>0 Ethics committee not mentioned
2014	Kurt 2014<br>doi.org/10.1016%2Fj.joms.2014.02.004	Kurt SN, Ustun Y, Erdogan O, Evlice B, Yoldas O, Oztunc H. Outcomes of periradicular surgery of maxillary first molars using a vestibular approach: a prospective, clinical study with one year of follow-up. Journal of Oral and Maxillofacial Surgery 2014;72:1049-61<br>2 approved by the ethical committee of Cukurova University (21.05.200:5:13)
2012	Del Fabbro 201<br>doi.org/10.1016%2Fj.joen.2012.02.022	Del Fabbro M, Ceresoli V, Lolato A, Taschieri S. Effect of platelet concentrate on quality of life after periradicular surgery: a randomized clinical study. Journal of Endodontics 2012;38:733-9<br>1 approved by the Institutional Review Board of Milan University
2012	Song 2012<br>doi.org/10.1016%2Fj.joen.2012.04.008	Song M, Kim E. A prospective randomized controlled study of mineral trioxide aggregate and super ethoxy-benzoic acid as root-end filling materials in endodontic microsurgery. Journal of Endodontics 2012;38:875-9<br>1 approved by the Yonsei University Committee for Research on Human Subjects
2011	Walivaara 2011<br>doi.org/10.1016%2Fj.tripleo.2011.01.016	Walivaara D-A, Abrahamsson P, Fogelin M, Isaksson S. Super-EBA and IRM as root-end fillings in periapical surgery with ultrasonic preparation: a prospective randomized clinical study of 206 consecutive teeth. Oral Surgery, Oral Medicine, Oral Pathology, Oral Radiology, and Endodontics 2011;112:258-63<br>1 approved by the human ethical committee at the University of Lund, Sweden
2009	Christiansen 20<br>doi.org/10.1111/j.1365-2591.2008.01474.x	Christiansen R, Kirkevang L-L, Horsted-Bindslev P, Wenzel A. Randomized clinical trial of root-end resection followed by root-end filling with mineral trioxide aggregate or smoothing of the orthograde guttapercha root filling - 1-year follow-up. International Endodontic Journal 2009;42:105-14<br>1 approved by the regional Committee of Ethics
2009	Del Fabbro 200<br>doi.org/10.1111/j.1365-2591.2008.01534.x	Del Fabbro M, Taschieri S, Weinstein R. Quality of life after microscopic periradicular surgery using two different incision techniques: a randomized clinical study. International Endodontic Journal 2009;42:360-7<br>1 approved by the Institutional Review Board of Milan University

Cochrane_Author		Primary studies_	doi	
2009	Walivaara 2009	Walivaara D-A, Abrahamsson P, Samfors K-A, Isaksson S. Periapical surgery using ultrasonic preparation and thermoplasticized gutta-percha with AH Plus sealer or IRM as retrograde root-end fillings in 160 consecutive teeth: a prospective randomized clinical study. Oral Surgery, Oral Medicine, Oral Pathology, Oral Radiology, and Endodontics 2009:108:784-9	doi.org/10.1016%2Fj.tripleo.2009.06.010	0 Ethics committee not mentioned
2008	Taschier 2008	Taschieri S, Del Fabbro M, Testori T, Weinstein R. Microscope versus endoscope in root-end management: a randomized controlled study. International Journal of Oral and Maxillofacial Surgery 2008;37:1022-6	doi.org/10.1016%2Fj.ijom.2008.07.001	0 Ethics committee not mentioned
2007	De Lange 2007	De Lange J, Putters T, Baas EM, Van Ingen JM. Ultrasonic root-end preparation in apical surgery: a prospective randomized study. Oral Surgery, Oral Medicine, Oral Pathology, Oral Radiology, and Endodontics 2007;104:841-5	doi.org/10.1016%2Fj.tripleo.2007.06.023	0 Ethics committee not mentioned
2007	Taschier 2007	Taschieri S, Del Fabbro M, Testori T, Weinstein R. Efficacy of xenogeneic bone grafting with guided tissue regeneration in the management of bone defects after surgical endodontics. Journal of Oral & Maxillofacial Surgery 2007;65:1121-7	doi.org/10.1016%2Fj.joms.2006.10.022	--- (article has not been accessed online) ---
2005	Lindeboom 200	Lindeboom JAH, Frenken JWH, Valkenburg P, Van den Akker HP. The role of preoperative prophylactic antibiotic administration in periapical endodontic surgery: a randomized, prospective double-blind placebo-controlled study. International Endodontic Journal 2005;38:877-81	doi.org/10.1111/j.1365-2591.2005.01030.x	1 approved by the medical ethical committee of the Academic Medical Centre of Amsterdam
2005	Lindeboom 200	Lindeboom JAH, Frenken JWFH, Kroon FHM, Van den Akker HP. A comparative prospective randomized clinical study of MTA and IRM as root-end filling materials in single-rooted teeth in endodontic surgery. Oral Surgery, Oral Medicine, Oral Pathology, Oral Radiology, and Endodontology 2005;100(4):495-500	doi.org/10.1016%2Fj.tripleo.2005.03.027	0 Ethics committee not mentioned
2005	Payer 2005	Payer M, Jakse N, Pertl C, Truschnegg A, Lechner E, Eskici A. The clinical effect of LLLT in endodontic surgery: a prospective study on 72 cases. Oral Surgery, Oral Medicine, Oral Pathology, Oral Radiology, and Endodontology 2005;100:375-9	doi.org/10.1016%2Fj.tripleo.2004.10.006	0 Ethics committee not mentioned
2004	Velvart 2004	Velvart P, Ebner-Zimmermann U, Ebner JP. Comparison of long-term papilla healing following sulcular full thickness flap and papilla base flap in endodontic surgery. International Endodontic Journal 2004;37:687-93	doi.org/10.1111/j.1365-2591.2004.00852.x	0 Ethics committee not mentioned
2003	Chong 2003	Chong BS, Pitt Ford TR, Hudson MB. A prospective clinical study of Mineral Trioxide Aggregate and IRM when used as root-end filling materials in endodontic surgery. International Endodontic Surgery 2003;36:520-6	doi.org/10.1046/j.1365-2591.2003.00682.x	1 approved by the local ethical committee

2001 Pecora 2001 Pecora G, De Leonardis D, Ibrahim N, Bovi M, Cornelini R. The use of calcium sulphate in the surgical treatment of a 'through and through' periradicular lesion. International Endodontic Journal 2001;34:189-97
doi.org/10.1046/j.1365-2591.2001.00369.x
0 Ethics committee not mentioned

1999 Kvist 1999 Kvist T, Reit C. Results of endodontic retreatment: a randomized clinical study comparing surgical and nonsurgical procedures. Journal of Endodontics 1999;25(12):71-4
doi.org/10.1016%2FS0099-2399%2899%2980304-5
0 Ethics committee not mentioned

1996 Danin 1996 Danin J, Stromberg T, Forsgren H, Linder LE, Ramskold LO. Clinical management of nonhealing periradicular pathosis. Surgery versus endodontic retreatment. Oral Surgery, Oral Medicine, Oral Pathology, Oral Radiology, and Endodontics 1996;82(2):814-7
doi.org/10.1016%2FS1079-2104%2896%2980259-9
0 Ethics committee not mentioned

1991 Zetterqvist 199 Zetterqvist L, Hall G, Holmlund A. Apicectomy: a comparative study of amalgam and glass ionomer cement as apical sealants. Oral Surgery, Oral Medicine, Oral Pathology 1991;71:489-91
doi.org/10.1016%2F0030-4220%2891%2990437-H
1 approved by the local ethical committee of Huddinge Hospital

Del Fabbro M, Taschieri S, Lodi G, Banfi G, Weinstein RL http://doi.org/10.1002/14651858.CD005969.pub3
2015 Magnification devices for endodontic therapy Primary studies: 0

EMPTY Empty Systematic Review

Dorri M, Dunne SM, Walsh T, Schwendicke F http://doi.org/10.1002/14651858.CD010431.pub2
2015 Micro-invasive interventions for managing proximal dental decay in primary and permanent teeth Primary studies: 8

2012 Martignon 2012 Martignon S, Ekstrand KR, Gomex J, Lara JS, Cortes A. Infiltrating/sealing proximal caries lesions: a 3-year randomized clinical trial. Journal of Dental Research 2012;91(3):288-92.
doi.org/10.1177%2F0022034511435328
2 approved by the Ethical Board at the Universidad El Bosque (IRB-089, 2008)

2011 Alkilzy 2011 Alkilzy M, Berndt C, Splieth CH. Sealing proximal surfaces with polyurethane tape: three-year evaluation. Clinical Oral Investigations 2011;15(6):879-84.
doi.org/10.1007%2Fs00784-010-0457-z
1 approved by the ethics committee

2011 Trairatvorakul 2 Trairatvorakul C, Itsaraviriyakul S, Wiboonchan W. Effect of glass-ionomer cement on the progression of proximal caries. Journal of Dental Research 2011;90(1):99-103.
doi.org/10.1177%2F0022034510381265
1 approved by the Chulalongkorn University Institutional Ethics Committee

2010 Ekstrand 2010
doi.org/10.1159%2F000275573
Ekstrand KR, Bakhshandeh A, Martignon S. Treatment of proximal superficial caries lesions on primary molar teeth with resin infiltration and fluoride varnish versus fluoride varnish only: efficacy after 1 year. Caries Research 2010;44(1):41-6.
2 approved by the ethics board in Nuuk (31 January 2008, 011-0027-08/Jh)

2010 Martignon 2010
doi.org/10.1159%2F000321986
Martignon S, Tellez M, Santamaría RM, Gomez J, Ekstrand KR. Sealing distal proximal caries lesions in first primary molars: efficacy after 2.5 years. Caries Research 2010;44(6):562-70.
2 approved by the Ethical Board of the Universidad El Bosque (UB155–2007

2010 Paris2010a
doi.org/10.1159%2F000341807
Meyer-Lueckel H, Bitter K, Paris S. Randomized controlled clinical trial on proximal caries infiltration: three-year follow-up. Caries Research 2012;46(6):544-8.
2 approved by the local institutional board at Charité – Universitätsmedizin Berlin (EA4/053/07)

2006 Martignon 2006
doi.org/10.1159%2F000094282
Martignon S, Ekstrand KR, Ellwood R. Efficacy of sealing proximal early active lesions: an 18-month clinical study evaluated by conventional and subtraction radiography. Caries Research 2006;40(5):382-8.
1 approved by the Ethical Boards in Denmark and Colombia

2005 Gomez 2005
doi.org/10.1007%2Fs00784-005-0010-7
Gomez SS, Basili CP, Emilson CG. A 2-year clinical evaluation of sealed noncavitated approximal posterior carious lesions in adolescents. Clinical Oral Investigations 2005;9(4):239-43.
1 approved by the ethics committee of the Valparaiso University

Eberhard J, Jepsen S, Jervoe-Storm PM, Needleman I, Worthington HV http://doi.org/10.1002/14651858.CD004622.pub3

2015 Full-mouth treatment modalities (within 24 hours) for chronic periodontitis in adults Primary studies: 12

2010 Zijnge 2010
doi.org/10.1111/j.1600-051X.2010.01562.x
Zijnge V, Meijer HF, Lie MA, Tromp JAH, Degener JE, Harmsen HJM, et al. The recolonization hypothesis in a full-mouth or multiple-session treatment protocol: a blinded, randomized clinical trial. Journal of Clinical Periodontology 2010;37:518-25.
1 approved by the Ethical Committee of the University Medical Center Groningen

2009 Swierkot 2009
doi.org/10.1111/j.1600-051X.2008.01368.x
Swierkot K, Nonnenmacher CL, Mutters R, Flores-de-Jacoby L, Mengel R. One-stage full-mouth disinfection versus quadrant and full-mouth root planing. Journal of Clinical Periodontology 2009;36:240-9.
0 Ethics committee not mentioned

2008 Del Peloso 2008
doi.org/10.1111/j.1600-051X.2008.01292.x
Del Peloso RE, Bittencourt S, Sallum ER, Nociti FH Jr, Goncalves RB, Casati MZ. Periodontal debridement as a therapeutic approach for severe chronic periodontitis: a clinical, microbiological and immunological study. Journal of Clinical Periodontology 2008;35:789-98.
2 approved by the Ethics Committee of the University of Campinas – UNICAMP (126/2005)

Cochrane_Author		Primary studies_	doi	
2007	Knöfler 2007	Knöfler GU, Purschwtz RE, Jentsch HFR. Clinical evaluation of partial- and full-mouth scaling in the treatment of chronic periodontitis. Journal of Periodontology 2007;78:2135-42.	doi.org/10.1902%2Fjop.2007.070010	1 approved by the Ethical Committee of the Medical Faculty of the University of Leipzig
2006	Jervøe-Storm 2	Jervøe-Storm PM, Semaan E, AlAhdab H, Engel S, Fimmers R, Jepsen S. Clinical outcomes of quadrant root planing versus full-mouth root planing. Journal of Clinical Periodontology 2006;33(3):209-15.	doi.org/10.1111/j.1600-051X.2005.00890.x	1 approved by an international ethics committee (IRB/IEC, Freiburg, Germany)
2006	Quirynen 2006	Quirynen M, De Soete M, Boschmans G, Pauwels M, Coucke W, Teughels W, et al. Benefit of "one-stage full-mouth disinfection" is explained by disinfection and root planing within 24 hours: a randomized controlled trial. Journal of Clinical Periodontology 2006;33(9):639-47.	doi.org/10.1111/j.1600-051X.2006.00959.x	1 approved by the Clinical Trials Committee of the University Hospital
2006	Zanatta 2006	Zanatta GM, Bittencourt S, Nociti FH Jr, Sallum EA, Sallum AW, Casati MZ. Periodontal debridement with povidone-iodine in periodontal treatment: short-term clinical and biochemical observations. Journal of Periodontology 2006;77(3):498-505.	doi.org/10.1902%2Fjop.2006.050154	1 approved by the Ethics Committee of the School of Dentistry at Piracicaba, University of Campinas
2005	Koshy 2005	Koshy G, Kawashima Y, Kiji M, Nitta H, Umeda M, Nagasawa T, et al. Effects of single-visit full-mouth ultrasonic debridement versus quadrant-wise ultrasonic debridement. Journal of Clinical Periodontology 2005;32(7):734-43.	doi.org/10.1111/j.1600-051X.2005.00775.x	1 approved by the Ethics Committee of the Tokyo Medical and Dental University
2005	Wennström 20	Wennström JL, Tomasi C, Bertelle A, Dellasega E. Full-mouth ultrasonic debridement versus quadrant scaling and root planing as an initial approach in the treatment of chronic periodontitis. Journal of Clinical Periodontology 2005;32(8):851-9.	doi.org/10.1111/j.1600-051X.2005.00776.x	1 approved by the Ethics Committee at Göteborg University
2004	Apatzidou 2004	Apatzidou DA, Kinane DF. Quadrant root planing versus same-day full-mouth root planing. I. Clinical findings. Journal of Clinical Periodontology 2004;31(2):132-40.	doi.org/10.1111/j.0303-6979.2004.00461.x	0 Ethics committee not mentioned
1999	Mongardini 199	Mongardini C, van Steenberghe D, Dekeyser C, Quirynen M. One stage full- versus partial-mouth disinfection in the treatment of chronic adult or generalized early-onset periodontitis. I. Long-term clinical observations. Journal of Periodontology 1999;70(6):632-45.	doi.org/10.1902%2Fjop.1999.70.6.632	1 approved by the Clinical Trials Committee of the University Hospital
1996	Vandekerckhov	Vandekerckhove BN, Bollen CM, Dekeyser C, Darius P, Quirynen M. Full- versus partial-mouth disinfection in the treatment of periodontal infections. Long-term clinical observations of a pilot study. Journal of Periodontology 1996;67(12):1251-9.	doi.org/10.1902%2Fjop.1996.67.12.1251	1 approved by the Ethical Committee of the University Hospital

Cochrane_Author Primary studies_ doi

El-Angbawi A, McIntyre GT, Fleming PS, Bearn DR http://doi.org/10.1002/14651858.CD010887.pub2

2015 Non-surgical adjunctive interventions for accelerating tooth movement in patients undergoing fixed orthodontic treat Primary studies: 2

2015 Pavlin 2015 doi.org/10.1053%2Fj.sodo.2015.06.005
Pavlin D, Anthony R, Raj V, Gakunga PT. Cyclic loading (vibration) accelerates tooth movement in orthodontic patients: A double-blind, randomized controlled trial. Seminars in Orthodontics 2015;21(3):187-94.
2 approved by the U.S. Food and Drug Administration (FDA) under an Investigational Device Exemption (IDE-G080191)

2012 Miles 2012 ncbi.nlm.nih.gov/pubmed/23304970
Miles P, Smith H, Weyant R, Rinchuse DJ. The effects of a vibrational appliance on tooth movement and patient discomfort: a prospective randomised clinical trial. Australian Orthodontic Journal 2012;28(2):213-8
1 approved by the Institutional Review Board at Seton Hill University, Greensburg, Pennsylvania, USA

Eliyas S, Al-Khayatt A, Porter RW, Briggs P http://doi.org/10.1002/14651858.CD008857.pub2

2013 Dental extractions prior to radiotherapy to the jaws for reducing post-radiotherapy dental complications Primary studies: 0

EMPTY
Empty Systematic Review

Esposito M, Ardebili Y, Worthington HV http://doi.org/10.1002/14651858.CD003815.pub4

2014 Interventions for replacing missing teeth: different types of dental implants Primary studies: 27

2014 Pozzi 2014 doi.org/10.1111/j.1708-8208.2012.00465.x
Pozzi A, Agliardi E, Tallarico M, Barlattani A. Clinical and radiological outcomes of two implants with different prosthetic interfaces and neck configurations: randomized, controlled, split-mouth clinical trial.. Clin Implant Dent Relat Res 2014; 16: 96-106 [Epub 2012]
0 Ethics committee not mentioned

2013 Esposito 2013a ncbi.nlm.nih.gov/pubmed/24179977
Esposito M, Dojcinovic I, Germon L, Lévy N, Curno R, Buchini S, Péchy P, Aronsson BO. Safety and efficacy of a biomimetic monolayer of permanently bound multi-phosphonic acid molecules on dental implants: 1 year post-loading results from a pilot quadruple-blinded randomised controlled trial. Eur J Oral Implantol 2013; 6: 227-236
2 approved by the Ethics Committee of Lausanne (214/07) and authorized by SwissMedic (2008-MD-0024)

2012 Al-Nawas 2012 doi.org/10.1111/j.1708-8208.2010.00324.x
Al-Nawas B, Brägger U, Meijer HJ, Naert I, Persson R, Perucchi A, Quirynen M, Raghoebar GM, Reichert TE, Romeo E, Santing H J, Schimmel M, Storelli S, Bruggenkate CT, Vandekerckhove B, Wagner W, Wismeijer D, Muller F. A Double-Blind Randomized Controlled Trial (RCT) of Titanium-13Zirconium versus Titanium Grade IV Small-Diameter Bone Level Implants in Edentulous Mandibles - Results from a 1-Year Observation Period.. Clin Implant Dent Relat Res 2012; 14: 896-904 [Epub 2011]
1 approved by the Ethics Committee s of all study sites

2012 Esposito 2012 ncbi.nlm.nih.gov/pubmed/23000708
Esposito M, Grusovin MG, Pellegrino G, Soardi E, Felice P. Safety and effectiveness of maxillary early loaded titanium implants with a novel nanostructured calcium-incorporated surface (Xpeed): 1-year results from a pilot multicenter randomised controlled trial.. Eur J Oral Implantol 2012; 5: 241-249
0 Ethics committee not mentioned

Cochrane_Author		Primary studies_	doi
2011	Akoglu 2011	Akoglu B, Ucankale M, Ozkan Y, Kulak-Ozkan Y. Five-year treatment outcomes with three brands of implants supporting mandibular overdentures. Int J Oral Maxillofac Implants 2011; 26: 188-194	
ncbi.nlm.nih.gov/pubmed/21365055		1 approved by the Ethics Committee	
2011	Alsabeeha 2011	Alsabeeha NH, Payne AG, De Silva RK, Thomson WM. Mandibular single-implant overdentures: preliminary results of a randomised-control trial on early loading with different implant diameters and attachment systems . Clin Oral Implants Res 2011; 22: 330-337 [Epub 2010]	
doi.org/10.1111/j.1600-0501.2010.02004.x		1 approved by the Lower South Regional Ethics Committee , New Zealand	
2011	den Hartog 201	den Hartog L, Meijer HJ, Stegenga B, Tymstra N, Vissink A, Raghoebar GM. Single implants with different neck designs in the aesthetic zone: a randomized clinical trial. Clin Oral Implants Res 2011; 22: 1289-1297	
doi.org/10.1111/j.1600-0501.2010.02109.x		1 approved by the Medical Ethics Committee of the University Medical Center Groningen	
2011	Heberer 2011	Heberer S, Kilic S, Hossamo J, Raguse JD, Nelson K. Rehabilitation of irradiated patients with modified and conventional sandblasted acid-etched implants: preliminary results of a split-mouth study . Clin Oral Implants Res 2011; 22: 546-551 [Epub 2010]	
doi.org/10.1111/j.1600-0501.2010.02050.x		1 approved by the Ethic Committee of the Charité University Medicine in Berlin, Germany	
2009	Crespi 2009	Crespi R, Cappare P, Gherlone E. Radiographic evaluation of marginal bone levels around platform-switched and non-platform-switched implants used in an immediate loading protocol.. Int J Oral Maxillofac Implants 2009; 24: 920-926	
ncbi.nlm.nih.gov/pubmed/19865633		1 approved by the local ethical committee	
2009	Kielbassa 2009	Kielbassa AM, Martinez-de Fuentes R, Goldstein M, Arnhart C, Barlattani A, Jackowski J, Knauf M, Lorenzoni M, Maiorana C, Mericske-Stern R, Rompen E, Sanz M. Randomized controlled trial comparing a variable-thread novel tapered and a standard tapered implant: Interim one-year results. J Prosthet Dent 2009; 101: 293-305	
doi.org/10.1016%2FS0022-3913%2809%2960060-3		1 approved by the Ethical Review Committees at each of the 12 study centers	
2009	Prosper 2009	Prosper L, Radaelli S, Pasi M, Zarone F, Radaelli G, Gherlone EF. A Randomized Prospective Multicenter Trial Evaluating the Platfom-Switching Technique for the Prevention of Postrestorative Crestal Bone Loss. Int J Oral Maxillofac Implants 2009; 24: 299-308	
ncbi.nlm.nih.gov/pubmed/19492646		1 approved by the research Ethics Committee at San Raffaele Hospital, milan, Italy	
2009	Song 2009	Song DW, Lee DW, Kim CK, Park KH, Moon IS. Comparative Analysis of Peri-Implant Marginal Bone Loss Based on Microthread Location: A 1-Year Prospective Study After Loading. J Periodontol 2009; 80: 1937-1944	
doi.org/10.1902%2Fjop.2009.090330		1 approved by the Institutional Review Board of Yonsei University	
2007	Lang 2007	Lang NP, Tonetti MS, Suvan JE, Pierre Bernard J, Botticelli D, Fourmousis I, Hallund M, Jung R, Laurell L, Salvi GE, Shafer D, Weber HP; European Research Group on Periodontology. Immediate implant placement with transmucosal healing in areas of aesthetic priority. A multicentre randomized-controlled clinical trial I. Surgical outcomes.. Clin Oral Implants Res 2007; 18: 188-196	
doi.org/10.1111/j.1600-0501.2006.01371.x		2 approved by the Ethics Committee of the Canton Bern Switzerland (KEK-nr. 218/01)	

Year	Author	Primary study	Ethics
2007	Lee 2007 doi.org/10.1111/j.1600-0501.2007.01302.x	Lee DW, Choi YS, Park KH, Kim CS, Moon IS. Effect of microthread on the maintenance of marginal bone level: a 3-year prospective study.. Clin Oral Implants Res 2007; 18: 465-470	1 approved by the Yonsei University Ethics Committee
2007	Schincaglia 200 ncbi.nlm.nih.gov/pubmed/17340895	Schincaglia GP, Marzola R, Scapoli C, Scotti R. Immediate loading of dental implants supporting fixed partial dentures in the posterior mandible: A randomized controlled split-mouth study – machined vs titanium oxide implant surface. Int J Oral Maxillofac Implants 2007; 22: 35-46	1 approved by the Ethics Committee of the University of Bologna approved the research protocol
2006	Fröberg 2006 doi.org/10.1111/j.1708-8208.2006.00017.x	Fröberg KK, Lindh C, Ericsson I. Immediate loading of Brånemark System Implants: a comparison between TiUnite and turned implants placed in the anterior mandible. Clin Implant Dent Relat Res 2006; 8: 187-197	0 Ethics committee not mentioned
2004	Payne 2004 doi.org/10.1111/j.1708-8208.2004.tb00028.x	Payne AG, Tawse-Smith A, Thomson WM, Duncan WD, Kumara R. One-stage surgery and early loading of three implants for maxillary overdentures: a 1-year report. Clin Implant Dent Relat Res 2004; 6: 61-74	1 approved by the Otago Ethics Committee
2004	Wennström 20 doi.org/10.1111/j.1600-051X.2004.00568.x	Wennström JL, Ekestubbe A, Gröndahl K, Karlsson S, Lindhe J. Oral rehabilitation with implant-supported fixed partial dentures in periodontitis-susceptible subjects. J Clin Periodontol 2004; 31: 713-724	1 approved by the Ethics Committee at the Sahlgrenska Academy, Göteborg University
2003	Payne 2003 doi.org/10.1111/j.1708-8208.2003.tb00196.x	Payne AG, Tawse-Smith A, Thomson WM, Kumara R. Early functional loading of unsplinted roughened surface implants with mandibular overdentures 2 weeks after surgery. Clin Implant Dent Relat Res 2003; 5: 143-153	0 Ethics committee not mentioned
2002	Astrand 2002 doi.org/10.1111/j.1708-8208.2002.tb00161.x	Åstrand P, Engquist B, Anzen B, Bergendal T, Hallman M, Karlsson U, Kvint S, Lysell L, Rundcrantz T. Nonsubmerged and submerged implants in the treatment of partially edentulous maxilla. Clin Implant Dent Relat Res 2002; 4: 115-127	1 approved by the research Ethics Committee at the Linköping University Hospital
2002	Gatti 2002 doi.org/10.1111/j.1708-8208.2002.tb00171.x	Gatti C, Chiapasco M. Immediate loading of Brånemark implants: a 24-month follow-up of a comparative prospective pilot study between mandibular overdentures supported by conical transmucosal and standard MKII implants. Clin Implant Dent Relat Res 2002; 4: 190-199	0 Ethics committee not mentioned
2002	Heydenrijk 200 doi.org/10.1034/j.1600-0501.2002.130405.x	Heydenrijk K, Raghoebar GM, Meijer HJ, van der Reijden WA, van Winkelhoff AJ, Stegenga B. Two-stage IMZ implants and ITI implants inserted in a single-stage procedure. A prospective comparative study. Clin Oral Implants Res 2002; 13: 371-380	0 Ethics committee not mentioned

2002 Tawse-Smith 20 [doi.org/10.1111/j.1708-8208.2002.tb00149.x](doi.org/10.1111/j.1708-8208.2002.tb00149.x)	Tawse-Smith A, Payne AG, Kumara R, Thomson WM. Early loading of unsplinted implants supporting mandibular overdentures using a one-stage operative procedure with two different implant systems: a 2-year report. Clin Implant Dent Relat Res 2002; 4: 33-42	1 approved by the Otago Ethics Committee
2001 Moberg 2001 [doi.org/10.1034/j.1600-0501.2001.120504.x](doi.org/10.1034/j.1600-0501.2001.120504.x)	Moberg LE, Köndell PA, Sagulin GB, Bolin A, Heimdahl A, Gynther GW. Brånemark system and ITI Dental Implant System for treatment of mandibular edentulism. Clin Oral Implants Res 2001; 12: 450-461	0 Ethics committee not mentioned
2001 Tawse-Smith 20 [doi.org/10.1111/j.1708-8208.2001.tb00140.x](doi.org/10.1111/j.1708-8208.2001.tb00140.x)	Tawse-Smith A, Payne AG, Kumara R, Thomson WM. One-stage operative procedure using two different implant systems: a prospective study on implant overdentures in the edentulous mandible. Clin Implant Dent Relat Res 2001; 3: 185-193	1 approved by the Otago Ethics Committee
1999 Astrand 1999 [doi.org/10.1111/j.1708-8208.1999.tb00087.x](doi.org/10.1111/j.1708-8208.1999.tb00087.x)	Åstrand P, Engquist B, Dahlgren S, Engquist E, Feldmann H, Gröndahl K. Astra Tech and Brånemark System implants: a prospective 5-year comparative study. Results after one year. Clin Implant Dent Relat Res 1999; 1: 17-26	0 Ethics committee not mentioned
1998 Batenburg 1998 [doi.org/10.1034/j.1600-0501.1996.090603.x](doi.org/10.1034/j.1600-0501.1996.090603.x)	Batenburg RH, Meijer HJA, Raghoebar GM, Van Oort RP, Boering G. Mandibular overdentures supported by two Brånemark, IMZ or ITI implants. A prospective comparative preliminary study: one-year results. Clin Oral Implants Res 1998; 9: 374-383	0 Ethics committee not mentioned

Esposito M, Felice P, Worthington HV http://doi.org/10.1002/14651858.CD008397.pub2

2014 Interventions for replacing missing teeth: augmentation procedures of the maxillary sinus Primary studies: 18

2013 Felice 2013 ncbi.nlm.nih.gov/pubmed/23926587	Felice P, Pistilli R, Piattelli M, Soardi E, Pellegrino G, Corvino V, Esposito M. 1-stage versus 2-stage lateral maxillary sinus lift procedures: 4-month post-loading results of a multicenter randomised controlled trial.. Eur J Oral Implantol 2013; 6: 153-165	0 Ethics committee not mentioned
2013 Merli 2013 ncbi.nlm.nih.gov/pubmed/23513200	Merli M, Moscatelli M, Mariotti G, Rotundo R, Nieri M. Autogenous bone versus deproteinised bovine bone matrix in 1-stage lateral sinus floor elevation in the severely atrophied maxilla: a randomised controlled trial.. Eur J Oral Implantol 2013; 6: 27-37	0 Ethics committee not mentioned
2013 Rickert 2013 [doi.org/10.1111/j.1708-8208.2011.00364.x](doi.org/10.1111/j.1708-8208.2011.00364.x)	Rickert D, Vissink A, Slater JJ, Meijer HJ, Raghoebar GM. Comparison between conventional and piezoelectric surgical tools for maxillary sinus floor elevation. A randomized controlled clinical trial.. Clin Implant Dent Relat Res 2013; 15: 297-302 [Epub 2011]	0 Ethics committee not mentioned

Year	Cochrane_Author	doi	Primary studies_	Ethics
2013	Si 2013	doi.org/10.1111/jcpe.12066	Si MS, Zhuang LF, Gu YX, Mo JJ, Qiao SC, Lai HC. Osteotome sinus floor elevation with or without grafting: a 3-year randomized controlled clinical trial.. J Clin Periodontol 2013; 40: 396-403	1 approved by the Ethics Committee of Shanghai Ninth People's Hospital
2013	Torres 2012	doi.org/10.1111/jcpe.12153	Garcia-Denche JT, Wu X, Martinez PP, Eimar H, Ikbal DJ, Hernandez G, Lopez-Cabarcos E, Fernandez-Tresguerres I, Tamimi F. Membranes over the lateral window in sinus augmentation procedures: a two-arm and split-mouth randomized clinical trials.. J Clin Periodontol 2013; 40: 1043-1051	2 approved by the Ethics Committee for Clinical Trials of “San Carlos” University Hospital (# P-07/151)
2012	Crespi 2012	ncbi.nlm.nih.gov/pubmed/23057028	Crespi R, Cappare P, Gherlone E. Sinus floor elevation by osteotome: hand mallet versus electric mallet. A prospective clinical study.. Int J Oral Maxillofac Implants 2012; 27: 1144-1150	1 approved by the local ethical committee
2012	Esposito 2012	ncbi.nlm.nih.gov/pubmed/22518377	Esposito M, Cannizzaro G, Soardi E, Pistilli R, Piattelli M, Corvino V, Felice P. Posterior atrophic jaws rehabilitated with prostheses supported by 6 mm-long, 4 mm-wide implants or by longer implants in augmented bone. Preliminary results from a pilot randomised controlled trial . Eur J Oral Implantol 2012; 5: 19-33	0 Ethics committee not mentioned
2012	Felice 2012	ncbi.nlm.nih.gov/pubmed/24570980	Felice P, Pistilli R, Piattelli M, Soardi E, Corvino V, Esposito M. Posterior atrophic jaws rehabilitated with prostheses supported by 5 x 5 mm implants with a novel nanostructured calcium-incorporated titanium surface or by longer implants in augmented bone. Preliminary results from a randomised controlled trial.. Eur J Oral Implantol 2012: 5: 149-161	0 Ethics committee not mentioned
2012	Lindgren 2012	doi.org/10.1111/j.1708-8208.2010.00224.x	Lindgren C, Mordenfeld A, Hallman M. A Prospective 1-Year Clinical and Radiographic Study of Implants Placed after Maxillary Sinus Floor Augmentation with Synthetic Biphasic Calcium Phosphate or Deproteinized Bovine Bone. Clin Implant Dent Relat Res 2012; 14: 41-50 [Epub 2010]	1 approved by the regional ethics board, Uppsala
2011	Felice 2011	ncbi.nlm.nih.gov/pubmed/22043464	Felice P, Soardi E, Pellegrino G, Pistilli R, Marchetti C, Gessaroli M, Esposito M. Treatment of the atrophic edentulous maxilla: short implants versus bone augmentation for placing longer implants. Five-month post-loading results of a pilot randomised controlled trial. Eur J Oral Implantol 2011; 4: 191-202	0 Ethics committee not mentioned
2010	Checchi 2010		Checchi L, Felice P, Antonini ES, Cosci F, Pellegrino G, Esposito M. Crestal sinus lift for implant rehabilitation: a randomised clinical trial comparing the Cosci and the Summers techniques. A preliminary report on complications and patient reference.. Eur J Oral Implantol 2010; 3: 221-232	0 Ethics committee not mentioned
2010	Felice 2009a	ncbi.nlm.nih.gov/pubmed/21594216	Felice P, Checchi V, Pistilli R, Scarano A, Pellegrino G, Esposito M. Bone augmentation versus 5-mm dental implants in posterior atrophic jaws Four-month post-loading results from a randomized controlled clinical trial. Eur J Oral Implantol 2009; 2: 267-281	0 Ethics committee not mentioned

Cochrane_Author	Primary studies_	doi
2010 Felice 2009b ncbi.nlm.nih.gov/pubmed/21180682	Felice P, Scarano A, Pistilli R, Checchi L, Piatelli M, Pellegrino G, Esposito M. A comparison of two techniques to augment maxillary sinuses using the lateral window approach: rigid synthetic resorbable barriers versus anorganic bovine bone Five-month post-loading clinical and histological results of a pilot RCT. Eur J Oral Implantol 2009; 2: 293-306	
	0 Ethics committee not mentioned	
2009 Cannizzaro 200 ncbi.nlm.nih.gov/pubmed/20467616	Cannizzaro G, Felice P, Michele L, Paolo V, Esposito M. Early loading of implants in the atrophic posterior maxilla: lateral sinus lift with autogenous bone and Bio-Oss versus crestal mini sinus lift and 8-mm hydroxyapatite-coated implants:. Eur J Oral Implantol 2009; 2: 25-38	
	0 Ethics committee not mentioned	
2009 Torres 2009 doi.org/10.1111/j.1600-051X.2009.01437.x	Torres J, Tamimi F, Martinez PP, Alkhraisat MH, Linares R, Hernandez G, Torres-Macho J, Lopez-Cabarcos E. Effect of platelet-rich plasma on sinus lifting: a randomized-controlled clinical trial.. J Clin Periodontol 2009; 36: 677-687	
	1 approved by the Ethics Committee for clinical trials of the "Hospital San Carlos" (Madrid, Spain)	
2005 Raghoebar 200 doi.org/10.1111/j.1600-0501.2005.01115.x	Raghoebar GM, Schortinghuis J, Liem RS, Ruben JL, van der Wal JE, Vissink A. Does platelet-rich plasma promote remodeling of autologous bone grafts used for augmentation of the maxillary sinus floor?. Clin Oral Implants Res 2005; 16: 349-356	
	0 Ethics committee not mentioned	
2002 Hallman 2002 ncbi.nlm.nih.gov/pubmed/12381063	Hallman M, Sennerby L, Lundgren S. A clinical and histologic evaluation of implant integration in the posterior maxilla after sinus floor augmentation with autogenous bone, bovine hydroxyapatite, or a 20:80 mixture. Int J Oral Maxillofac Implants 2002; 17: 635-643	
	1 approved by the regional Ethics Committee	
2000 Wannfors 2000 ncbi.nlm.nih.gov/pubmed/11055129	Wannfors K, Johansson B, Hallman M, Strandkvist T. A prospective randomized study of 1- and 2-stage sinus inlay bone grafts: 1-year follow-up. Int J Oral Maxillofac Implants 2000; 15: 625-632	
	1 approved by the Ethics Committee at Karolinska Hospital, Stockholm, Sweden	

Esposito M, Grusovin MG, Maghaireh H, Worthington HV — http://doi.org/10.1002/14651858.CD003878.pub5

2013 Interventions for replacing missing teeth: different times for loading dental implants — Primary studies: 26

2012 Meloni 2012 ncbi.nlm.nih.gov/pubmed/23304688	Meloni SM, De Riu G, Pisano M, De Riu N, Tullio A. Immediate versus delayed loading of single mandibular molars. One-year results from a randomised controlled trial.. Eur J Oral Implantol 2012; 5: 345-353	
	2 Approved by the local ethics board at the U. of Sassari (MF2341)	
2011 De Rouck 2009 ncbi.nlm.nih.gov/pubmed/19302238	De Rouck T, Collys K, Wyn I, Cosyn J. Instant provisionalization of immediate single-tooth implants is essential to optimize esthetic treatment outcome. Clinical Oral Implants Research 2009;20(6):566-70.	
	2 approved by the Institutional Review Board of the University Medical Center Groningen (2010072)	

Cochrane_Author		Primary studies_	doi
2011	den Hartog 201	den Hartog L, Raghoebar GM, Stellingsma K, Vissink A, Meijer HJ. Immediate non-occlusal loading of single implants in the aesthetic zone: a randomized clinical trial. Journal of Clinical Periodontology 2011;38(2):186-94.	doi.org/10.1111/j.1600-051X.2010.01650.x
		1 approved by the Ethics Committee of the University Hospital in Brussels	
2010	Cannizzaro 201	Cannizzaro G, Torchio C, Felice P, Leone M, Esposito M. Immediate occlusal versus non-occlusal loading of single zirconia implants. A multicentre pragmatic randomised clinical trial. Eur J Oral Implantol 2010; 3: 111-120	ncbi.nlm.nih.gov/pubmed/20623036
		0 Ethics committee not mentioned	
2010	Enkling 2010	Enkling N, Albrecht D, Bayer S, Stark H, Mericske-Stern R. [Immediate loading of interforaminal implants using a chairside fabricated bur (oral presentation)]. Clinical Oral Implants Research. 2010; Vol. 21:1013.	
		--- (article has not been accessed online) ---	
2008	Cannizzaro 200	Cannizzaro G, Leone M, Esposito M. Immediate versus early loading of two implants placed with a flapless technique supporting mandibular bar-retained overdentures: a single-blinded, randomised controlled clinical trial.. Eur J Oral Implantol 2008; 1: 33-43	ncbi.nlm.nih.gov/pubmed/20467642
		0 Ethics committee not mentioned	
2008	Cannizzaro 200	Cannizzaro G, Torchio C, Leone M, Esposito M. Immediate versus early loading of flapless-placed implants supporting maxillary full-arch prostheses: a randomised controlled clinical trial. Eur J Oral Implantol 2008; 1: 127-139	
		0 Ethics committee not mentioned	
2008	Cannizzaro 200	Cannizzaro G, Leone M, Torchio C, Viola P, Esposito M. Immediate versus early loading of 7 mm long flapless-placed single implants: a split-mouth randomised controlled clinical trial. Eur J Oral Implantol 2008; 1: 277-292	ncbi.nlm.nih.gov/pubmed/20467635
		0 Ethics committee not mentioned	
2008	Crespi 2008	Crespi R, Cappare P, Gherlone E, Romanos GE. Immediate Versus Delayed Loading of Dental Implants Placed in Fresh Extraction Sockets in the Maxillary Esthetic Zone: A Clinical Comparative Study. Int J Oral Maxillofac Implants 2008; 23: 753-758	ncbi.nlm.nih.gov/pubmed/18807574
		0 Ethics committee not mentioned	
2008	Donati 2008	Donati M, La Scala V, Billi M, Di Dino B, Torrisi P, Berglundh T. Immediate functional loading of implants in single tooth replacement: a prospective clinical multicenter study. Clin Oral Implants Res 2008; 19: 740-748	doi.org/10.1111/j.1600-0501.2008.01552.x
		0 Ethics committee not mentioned	
2008	Güncü 2008	Guncu MB, Aslan Y, Tumer GN, Guncu GN, Uysal S. In-patient comparison of immediate and conventional loaded implants in mandibular molar sites within 12 months.. Clin Oral Implants Res 2008; 19: 335-341	doi.org/10.1111/j.1365-2842.2007.01844.x
		1 approved by the Ethics Committee	

Cochrane_Author		Primary studies_	doi
2008	Merli 2008	Merli M, Merli A, Bernadelli F, Lombardini F, Esposito M. Immediate versus early non-occlusal loading of dental implants placed flapless in partially edentulous patients. One-year results from a randomised controlled clinical trial. Eur J Oral Implantol 2008; 1: 207-220	ncbi.nlm.nih.gov/pubmed/18990996
		0 Ethics committee not mentioned	
2008	Schincaglia 200	Schincaglia G P, Marzola R, Giovanni G F, Chiara C S, Scotti R. Replacement of Mandibular Molars with Single-Unit Restorations Supported by Wide-Body Implants: Immediate Versus Delayed Loading. A Randomized Controlled Study. Int J Oral Maxillofac Implants 2008; 23: 474-480	ncbi.nlm.nih.gov/pubmed/18700371
		1 approved by the Ethics Committee of the University of Bologna	
2008	Zöllner 2008	Zöllner A, Ganeles J, Korostoff J, Guerra F, Krafft T, Brägger U. Immediate and early non-occlusal loading of Straumann implants with a chemically modified surface (SLActive) in the posterior mandible and maxilla: interim results from a prospective multicenter randomized-controlled study.. Clin Oral Implants Res 2008; 19: 442-450	doi.org/10.1111/j.1600-0501.2008.01626.x
		0 Ethics committee not mentioned	
2007	Assad 2007	Assad AS, Hassan SA, Shawky YM, Badawy MM. Clinical and radiographic evaluation of implant-retained mandibular overdentures with immediate loading.. Implant Dent 2007; 16: 212-223	doi.org/10.1097%2FID.0b013e318065a95f
		0 Ethics committee not mentioned	
2007	Testori 2007	Testori T, Galli F, Capelli M, Zuffetti F, Esposito M. Immediate nonocclusal versus early loading of dental implants in partially edentulous patients: 1-year results from a multicenter, randomized controlled clinical trial.. Int J Oral Maxillofac Implants 2007; 22: 815-822	ncbi.nlm.nih.gov/pubmed/17974118
		0 Ethics committee not mentioned	
2007	Turkyilmaz 200	Turkyilmaz I, Tumer C. Early versus late loading of unsplinted TiUnite surface implants supporting mandibular overdentures: a 2-year report from a prospective study.. J Oral Rehabil 2007; 34: 773-780	doi.org/10.1111/j.1365-2842.2006.01683.x
		1 approved by the Ethics Committee of Hacettepe University, Ankara, Turkey	
2006	Hall 2006	Hall JA, Payne AG, Purton DG, Torr B. A randomized controlled clinical trial of conventional and immediately loaded tapered implants with screw-retained crowns.. Int J Prosthodont 2006; 19: 17-19	ncbi.nlm.nih.gov/pubmed/16479752
		1 approved by the necessary ethical approvals and consents	
2006	Lindeboom 200	Lindeboom JA, Frenken JW, Dubois L, Frank M, Abbink I, Kroon FH. Immediate loading versus immediate provisionalization of maxillary single-tooth replacements: a prospective randomized study with BioComp implants.. J Oral Maxillofac Surg 2006; 64: 936-942	doi.org/10.1016%2Fj.joms.2006.02.015
		1 approved by the medical Ethics Committee of the Academic Medical Center	
2006	Oh 2006	Oh TJ, Shotwell JL, Billy EJ, Wang HL. Effect of flapless implant surgery on soft tissue profile: a randomized controlled clinical trial.. J Periodontol 2006; 77: 874-882	doi.org/10.1902%2Fjop.2006.050169
		1 approved by the Human Subjects Institutional Review Board of the University of Michigan	

2004	Fischer 2004	Fischer K, Stenberg T. Early loading of ITI implants supporting a maxillary full-arch prosthesis: 1-year data of a prospective, randomized study. Int J Oral Maxillofac Implants 2004; 19: 374-381	
ncbi.nlm.nih.gov/pubmed/15214221		0	Ethics committee not mentioned
2003	Cannizzaro 200	Cannizzaro G, Leone M. Restoration of partially edentulous patients using dental implants with a microtextured surface: a prospective comparison of delayed and immediate full occlusal loading. Int J Oral Maxillofac Implants 2003; 18: 512-522	
ncbi.nlm.nih.gov/pubmed/12939002		0	Ethics committee not mentioned
2002	Payne 2002	Payne AG, Tawse-Smith A, Duncan WD, Kumara R. Conventional and early loading of unsplinted ITI implants supporting mandibular overdentures. Clin Oral Implants Res 2002; 13: 603-609	
doi.org/10.1034/j.1600-0501.2002.130605.x		1	approved by the Otago Ethics Committee
2002	Romeo 2002	Romeo E, Chiapasco M, Lazza A, Casentini P, Ghisolfi M, Iorio M, Vogel G. Implant-retained mandibular overdentures with ITI implants. Clin Oral Implants Res 2002; 13: 495-501	
doi.org/10.1034/j.1600-0501.2002.130508.x		0	Ethics committee not mentioned
2002	Tawse-Smith 20	Tawse-Smith A, Payne AG, Kumara R, Thomson WM. Early loading of unsplinted implants supporting mandibular overdentures using a one-stage operative procedure with two different implant systems: a 2-year report. Clin Implant Dent Relat Res 2002; 4: 33-42	
doi.org/10.1111/j.1708-8208.2002.tb00149.x		1	approved by the Otago Ethics Committee
2001	Chiapasco 2001	Chiapasco M, Abati S, Romeo E, Vogel G. Implant-retained mandibular overdentures with Brånemark System MKII implants: a prospective comparative study between delayed and immediate loading. Int J Oral Maxillofac Implants 2001; 16: 537-546	
ncbi.nlm.nih.gov/pubmed/11516001		0	Ethics committee not mentioned

Esposito M, Grusovin MG, Worthington HV http://doi.org/10.1002/14651858.CD004152.pub4

2013 Interventions for replacing missing teeth: antibiotics at dental implant placement to prevent complications Primary studies: 6

2014	Nolan 2013	Nolan R, Kemmoona M, Polyzois I, Claffey N. The influence of prophylactic antibiotic administration on post-operative morbidity in dental implant surgery. A prospective double blind randomized controlled clinical trial. Clin Oral Implants Res 2014; 25: 252-259	
doi.org/10.1111/clr.12124		1	approved by the local Ethics Committee
2011	Caiazzo 2011	Caiazzo A, Casavecchia P, Barone A, Brugnami F. A pilot study to determine the effectiveness of different amoxicillin regimens in implant surgery.. J Oral Implantol 2011; 37: 691-696	
doi.org/10.1563%2FAAID-JOI-D-09-00134.1		0	Ethics committee not mentioned

2010	Esposito 2010a	Esposito M, Cannizarro G, Bozzoli P, Checchi L, Ferri V, Landriani S, Leone M, Todisco M, Torchio C, Testori T, Galli F, Felice P. Effectiveness of prophylactic antibiotics at placement of dental implants: a pragmatic multicentre placebo-controlled randomised clinical trial. Eur J Oral Implantol 2010; 3: 135-143
		0 Ethics committee not mentioned
2009	Anitua 2009	Anitua E, Aguirre JJ, Gorosabel A, Barrio P, Errazquin JM, Roman P, Pla R, Carrete J, de Pedro J, Orive G. A multicentre placebo-controlled randomized clinical trial of antibiotic prophylaxis for placement of single dental implants. Eur J Oral Implantol 2009; 2: 283-292
ncbi.nlm.nih.gov/pubmed/20467604		1 approved by the Basque country's Ethics Committee & by the Spanish Medicines Control Agency
2008	Abu-Ta'a 2008	Abu-Ta'a M, Quirynen M, Teughels W, van Steenberghe D. Asepsis during periodontal surgery involving oral implants and the usefulness of peri-operative antibiotics: a prospective, randomized, controlled clinical trial.. J Clin Periodontol 2008; 35: 58-63
ncbi.nlm.nih.gov/pubmed/18021264		1 approved by the Ethics Committee of the University Hospital of the Catholic University Leuven
2008	Esposito 2008a	Esposito M, Cannizzaro G, Bozzoli P, Consolo U, Felice P, Ferri V, Landriani S, Leone M, Magliano A, Pellitteri G, Todisco M, Torchio C. Efficacy of prophylactic antibiotics for dental implants: a multicentre placebo-controlled randomised clinical trial.. Eur J Oral Implantol 2008; 1: 23-31
ncbi.nlm.nih.gov/pubmed/20467641		0 Ethics committee not mentioned

Esposito M, Worthington HV http://doi.org/10.1002/14651858.CD003603.pub3

2013 Interventions for replacing missing teeth: hyperbaric oxygen therapy for irradiated patients who require dental implant Primary studies: 1

2007	Schoen 2007	Schoen PJ, Raghoebar GM, Bouma J, Reintsema H, Vissink A, Sterk W, et al. Rehabilitation of oral function in head and neck cancer patients after radiotherapy with implant-retained dentures: effects of hyperbaric oxygen therapy. Oral Oncology 2007;43(4):379-88.
doi.org/10.1016%2Fj.oraloncology.2006.04.009		0 Ethics committee not mentioned
	EMPTY	Empty Systematic Review

Fleming PS, Fedorowicz Z, Johal A, El-Angbawi A, Pandis N http://doi.org/10.1002/14651858.CD010572.pub2

2015 Surgical adjunctive procedures for accelerating orthodontic treatment Primary studies: 4

2014	Leethanakul 20	Leethanakul C, Kanokkulchai S, Pongpanich S, Leepong N, Charoemratrote C. Interseptal bone reduction on the rate of maxillary canine retraction. Angle Orthodontist 2014;84:839-45.
doi.org/10.2319%2F100613-737.1		1 approved by the ethical committee of the Faculty of Dentistry at the Prince of Songkla Universit

2013	Alikhani 2013	Alikhani M, Raptis M, Zoldan B, Sangsuwon C, Lee YB, Alyami B, et al. Effect of micro-osteoperforations on the rate of tooth movement. American Journal of Orthodontics and Dentofacial Orthopedics 2013;144(5):639-48.	
doi.org/10.1016%2Fj.ajodo.2013.06.017		1	approved by the institutional review board of New York University.
2011	Aboul-Ela 2011	Aboul-Ela SM, El-Beialy AR, El-Sayed KM, Selim EM, El-Mangoury NH, Mostafa YA. Miniscrew implant-supported maxillary canine retraction with and without corticotomy-facilitated orthodontics. American Journal of Orthodontics and Dentofacial Orthopedics 2011;139(2):252-9.	
doi.org/10.1016%2Fj.ajodo.2009.04.028		1	approved by the Ethics Review Committee of the dental school at Cairo University, Cairo, Egypt
2007	Fischer 2007	Fischer TJ. Orthodontic treatment acceleration with corticotomy-assisted exposure of palatally impacted canines. Angle Orthodontist 2007;77:417-20.	
angle.org/doi/10.2319/0003-3219(2007)077[0417:OTAWCE]2.0.CO;2		0	Ethics committee not mentioned

Fleming PS, Strydom H, Katsaros C, MacDonald L, Curatolo M, Fudalej P, Pan http://doi.org/10.1002/14651858.CD010263.pub2

2016 Non-pharmacological interventions for alleviating pain during orthodontic treatment Primary studies: 14

2016	Huang 2016	Huang R, Wang J, Wu D, Long H, Yang X, Liu H, et al. The effects of customised brainwave music on orofacial pain induced by orthodontic tooth movement. Oral Diseases 2016;22(8):766-74	
doi.org/10.1111/odi.12542		2	approved by the Ethics Committee of the West China Hospital of Stomatology (No. WCHSIRB-ST-2016-014; trial # ChiCTR-IOR-16007916)
2016	Miles 2016	Miles P, Fischer E. Assessment of the changes in arch perimeter and irregularity in the mandibular arch during initial alignment with the AcceleDent Aura appliance vs no appliance in adolescents: a single-blind randomized clinical trial. American Journal of Orthodontics and Dentofacial Orthopedics 2016;150(6):928-36	
doi.org/10.1016%2Fj.ajodo.2016.07.016		2	approved by the University of Queensland Dental Sciences Research Ethics Committee (project number 1315), Brisbane, Australia
2015	Lobre 2015	Lobre WD, Callegari BJ, Gardner G, Marsh CM, Bush AC, Dunn WJ. Pain control in orthodontics using a micropulse vibration device: A randomized clinical trial. Angle Orthodontist 2016;86(4):625-30	
doi.org/10.2319/072115-492.1		1	approved by the local institutional review board (IRB)
2015	Woodhouse 20	Woodhouse NR, DiBiase AT, Papageorgiou SN, Johnson N, Slipper C, Grant J, et al. Supplemental vibrational force does not reduce pain experience during initial alignment with fixed orthodontic appliances: a multicenter randomized clinical trial. Scientific Reports 2015;5:17224	
doi.org/10.1038%2Fsrep17224		2	approved by the United Kingdom National Research Ethics Service (South East London REC 3: 11/LO/0056)
2013	Keith 2013	Keith DJ, Rinchuse DJ, Kennedy M, Zullo T. Effect of text message follow-up on patient's self-reported level of pain and anxiety. Angle Orthodontist 2013;83(4):605-10	
doi.org/10.2319%2F091812-742.1		1	approved by the Seton Hill University Institutional Review Board

Year	Cochrane_Author	Primary studies_	Ethics
2013	Kim 2013<br>doi.org/10.2319%2F082012-665.1	Kim WT, Bayome M, Park JB, Park JH, Baek SH, Kook YA. Effect of frequent laser irradiation on orthodontic pain. A single-blind randomized clinical trial. Angle Orthodontist 2013;83(4):611-6	1 approved by the institutional review board of the Catholic University of Kore
2013	Nobrega 2013<br>doi.org/10.1089%2Fpho.2012.3338	Nóbrega C, da Silva EM, de Macedo CR. Low-level laser therapy for treatment of pain associated with orthodontic elastomeric separator placement: a placebo-controlled randomized double-blind clinical trial. Photomedicine and Laser Surgery 2013;31:10-16	2 approved by the ethics committee of Universidade Federal de Sao Paulo, according to document number CEP 0407/08
2012	Benson 2012<br>doi.org/10.1111/j.1601-6343.2012.01546.x	Benson PE, Razi RM, Al-Bloushi RJ. The effect of chewing gum on the impact, pain and breakages associated with fixed orthodontic appliances: a randomized clinical trial. Orthodontics & Craniofacial Research 2012;15(3):178-87	2 Approved by the South Sheffield Research Ethics Committee (reference number 07/H1309/96; November 2007)
2012	Farzanegan 201<br>doi.org/10.1016%2Fj.ajodo.2011.06.042	Farzanegan F, Zebarjad SM, Alizadeh S, Ahrari F. Pain reduction after initial archwire placement in orthodontic patients: a randomized clinical trial. American Journal of Orthodontics and Dentofacial Orthopedics 2012;141(2):169-73	1 approved by the research ethic committee of Mashhad University of Medical Sciences in Iran
2012	Miles 2012<br>ncbi.nlm.nih.gov/pubmed/23304970	Miles P, Smith H, Weyant R, Rinchuse DJ. The effects of a vibrational appliance on tooth movement and patient discomfort: a prospective randomised clinical trial. Australian Orthodontic Journal 2012;28(2):213-8	1 approved by the Institutional Review Board at Seton Hill University, Greensburg, Pennsylvania, USA
2007	Harazaki 2007<br>ncbi.nlm.nih.gov/pubmed/9566142	Harazaki M, Isshiki Y. Soft laser irradiation effects on pain reduction in orthodontic treatment. Bulletin of Tokyo Dental College 1997;38(4):291-5	--- (article has not been accessed online) ---
2006	Otasevic 2006<br>doi.org/10.1016%2Fj.ajodo.2005.11.033	Otasevic M, Naini FB, Gill DS, Lee RT. Prospective randomized clinical trial comparing the effects of a masticatory bite wafer and avoidance of hard food on pain associated with initial orthodontic tooth movement. American Journal of Orthodontics and Dentofacial Orthopedics 2006;130(1):e9-1	0 Ethics committee not mentioned
2006	Turhani 2006<br>doi.org/10.1016%2Fj.ajodo.2005.04.036	Turhani D, Scheriau M, Kapral D, Benesch T, Jonke E, Bantleon HP. Pain relief by single low-level laser irradiation in orthodontic patients undergoing fixed appliance therapy. American Journal of Orthodontics and Dentofacial Orthopedics 2006;130(3):371-7.	1 approved by the ethics commission of the Medical University of Vienna
2003	Marie 2003<br>ncbi.nlm.nih.gov/pubmed/12747073	Marie SS, Powers M, Sheridan JJ. Vibratory stimulation as a method of reducing pain after orthodontic appliance adjustment. Journal of Clinical Orthodontics 2003;37(4):205-8	--- (article has not been accessed online) ---

Cochrane_Author	Primary studies_	doi
Furness S, Bryan G, McMillan R, Worthington HV		http://doi.org/10.1002/14651858.CD009603.pub3
2013	Interventions for the management of dry mouth: non-pharmacological interventions	Primary studies: 9

Year	Study	Reference / Ethics
2010	Pfister 2010<br>doi.org/10.1200%2FJCO.2009.26.9860	Pfister DG, Cassileth BR, Deng GE, Yeung KS, Lee JS, Garrity D, et al. Acupuncture for pain and dysfunction after neck dissection: results of a randomized controlled trial. Journal of Clinical Oncology 2010;28(15):2565-70<br>1 approved by the Institutional Review Board at Memorial Sloan-Kettering Cancer Center (MSKCC)
2008	Cho 2008<br>doi.org/10.1089%2Facm.2007.0793	Cho JH, Chung WK, Kang W, Choi SM, Cho CK, Son CG. Manual acupuncture improved quality of life in cancer patients with radiation-induced xerostomia. The Journal of Alternative and Complementary Medicine 2008;14(5):523-6.<br>2 approved by the Institutional Review Board at Dunsan Oriental Hospital of Daejeon University and Konyang University Hospital (H2006-015)
2006	Papas 2006<br>doi.org/10.1111/j.1754-4505.2006.tb01661.x	Papas A, Singh M, Harrington D, Rodriguez S, Ortblad K, de Jager M, et al. Stimulation of salivary flow with powered toothbrush in a xerostomic population. Special Care Dentistry 2006;26(6):241-6.<br>1 approved by the Internal Review Board
2003	Wong 2003<br>doi.org/10.1016%2FS0360-3016%2803%2900572-8	Wong RKW, Jones GW, Sagar SM, Babjak A-F, Whelan T. A phase I-II study in the use of acupuncture-like transcutaneous nerve stimulation in the treatment of radiation-induced xerostomia in head-and-neck cancer patients treated with radical radiotherapy. International Journal of Radiation, Oncology, Biology and Physics 2003;57(2):472-80.<br>1 approved by the Research Ethics Board of the Henderson Hospital, Hamilton Health Sciences Corporation,
1998	List 1998<br>doi.org/10.1080%2F00016359850136058	List T, Lundeberg T, Lundström I, Lindström F, Ravald N. The effect of acupuncture in the treatment of patients with primary Sjögren's syndrome. Acta Odontologica Scandinavica 1998;56:95-9.<br>0 Ethics committee not mentioned
1996	Blom 1996<br>doi.org/10.1016%2F0964-1955%2895%2900085-2	Blom M, Dawidson I, Fernberg JO, Johnson G, Angmar-Månsson B. Acupuncture treatment of patients with radiation-induced xerostomia. Oral Oncology, European Journal of Cancer 1996;32B(3):182-90.<br>1 approved by the Human Ethics Committee at Huddinge Hospital, Karolinska Institutet.
1992	Blom 1992<br>doi.org/10.1016%2F0030-4220%2892%2990124-9	Blom M, Dawidson I, Angmar-Månsson B. The effect of acupuncture on salivary flow rates in patients with xerostomia. Oral Surgery, Oral Medicine, and Oral Pathology 1992;73(3):293-8.<br>1 approved by the Human Ethics Committee at Huddinge Hospital, Karolinska Institutet
1992	Talal 1992<br>doi.org/10.1007%2FBF00300975	Talal N, Quinn JH, Daniels TE. The clinical effects of electrostimulation on salivary function of Sjögren's syndrome patients: a placebo controlled trial. Rheumatology International 1992;12:43-5.<br>0 Ethics committee not mentioned

1988 Steller 1988 Steller M, Chou L, Daniels TE. Electrical stimulation of salivary flow in patients with Sjögren's Syndrome. Journal of Dental Research 1988;67:1334-7.

doi.org/10.1177%2F00220345880670101701

--- (article has not been accessed online) ---

Ghaeminia H, Perry J, Nienhuijs ME, Toedtling V, Tummers M, Hoppenreijs TJ http://doi.org/10.1002/14651858.CD003879.pub4

2016 Surgical removal versus retention for the management of asymptomatic disease-free impacted wisdom teeth Primary studies: 2

2013 Nunn 2013 Nunn ME, Fish MD, Garcia RI, Kaye EK, Figuerosa R, Gohel A, et al. Retained asymptomatic third molars and risk for second molar pathology. Journal of Dental Research 2013;92(12):1095-9.

doi.org/10.1177%2F0022034513509281

1 approved by the Veterans Affairs Subcommittee on Human Studies, and the Boston University Medical Campus Institutional Review Board

1998 Harradin 1998 Harradine NW, Pearson MH, Toth B. The effect of extraction of third molars on late lower incisor crowding: a randomized controlled trial. British Journal of Orthodontics 1998;25(2):117-22.

doi.org/10.1093%2Fortho%2F25.2.117

0 Ethics committee not mentioned

Glenny AM, Oliver R, Roberts GJ, Hooper L, Worthington HV http://doi.org/10.1002/14651858.CD003813.pub4

2013 Antibiotics for the prophylaxis of bacterial endocarditis in dentistry Primary studies: 0

EMPTY Empty Systematic Review

Hu H, Li C, Li F, Chen J, Sun J, Zou S, Sandham A, Xu Q, Riley P, Ye Q http://doi.org/10.1002/14651858.CD005516.pub2

2013 Enamel etching for bonding fixed orthodontic braces Primary studies: 13

2009 Cal-Neto 2009 Cal-Neto JP, Quintao CA, Almeida MA, Miguel JA. Bond failure rates with a self-etching primer: a randomized controlled trial. American Journal of Orthodontics & Dentofacial Orthopedics 2009;135(6):782-6.

doi.org/10.1016%2Fj.ajodo.2008.11.022

1 approved by the university's Research Ethics Committee

2009 Ghiz 2009 Ghiz MA, Ngan P, Kao E, Martin C, Gunel E. Effects of sealant and self-etching primer on enamel decalcification. Part II: an in-vivo study. American Journal of Orthodontics and Dentofacial Orthopedics 2009;135(2):206-13.

doi.org/10.1016%2Fj.ajodo.2007.02.060

0 Ethics committee not mentioned

2009 Paschos 2009 Paschos E, Kurochkina N, Huth KC, Hansson CS, Rudzki-Janson I. Failure rate of brackets bonded with antimicrobial and fluoride-releasing, self-etching primer and the effect on prevention of enamel demineralization. American Journal of Orthodontics and Dentofacial Orthopedics 2009;135(5):613-20.

doi.org/10.1016%2Fj.ajodo.2008.01.016

2 approved by the ethics committee of our university (no. 288/06)

Cochrane_Author		Primary studies_	doi
2008	Elekdag-Turk 20	Elekdag-Turk S, Cakmak F, Isci D, Turk T. 12-month self-ligating bracket failure rate with a self-etching primer. The Angle Orthodontist 2008;78(6):1095-100.	
doi.org/10.2319%2F112507-552.1		1 approval was obtained	
2008	Elekdag-Turk 20	Elekdag-Turk S, Isci D, Turk T, Cakmak F. Six-month bracket failure rate evaluation of a self-etching primer. European Journal of Orthodontics 2008;30(2):211-6.	
doi.org/10.1093%2Fejo%2Fcjm119		1 approval was obtained	
2007	Banks 2007	Banks P, Thiruvenkatachari B. Long-term clinical evaluation of bracket failure with a self-etching primer: a randomized controlled trial. Journal of Orthodontics 2007;34(4):243-51.	
doi.org/10.1179%2F146531207225022293		2 approved by the Oldham Local Research Ethics Committee (reference 03/OL/45)	
2006	House 2006	House K, Ireland AJ, Sherriff M. An investigation into the use of a single component self-etching primer adhesive system for orthodontic bonding: a randomized controlled clinical trial. Journal of Orthodontics 2006;33(1):38-44.	
doi.org/10.1179%2F146531205225021375		2 approved by the Central and South Bristol Local Research Ethics Committee, Project number E5506	
2006	Manning 2006	Manning N, Chadwick SM, Plunkett D, Macfarlane TV. A randomized clinical trial comparing 'one-step' and 'two-step' orthodontic bonding systems. Journal of Orthodontics 2006;33(4):276-83.	
doi.org/10.1179%2F146531205225021825		1 approved by the South Cheshire local research ethics committee	
2006	Murfitt 2006	Murfitt PG, Quick AN, Swain MV, Herbison GP. A randomised clinical trial to investigate bond failure rates using a self-etching primer. European Journal of Orthodontics 2006;28(5):444-9.	
doi.org/10.1093%2Fejo%2Fcjl007		1 approved by the Otago Ethics Committe	
2006	Noble 2006	Noble RR, Salas-Lopez A, English JD, Powers JM. Clinical evaluation of orthodontic self-etching primers. Texas Dental Journal 2006;123(3):274-8.	
ncbi.nlm.nih.gov/pubmed/16625957		--- (article has not been accessed online) ---	
2004	Aljubouri 2004	Aljubouri YD, Millett DT, Gilmour WH. Six and 12 months' evaluation of a self-etching primer versus two-stage etch and prime for orthodontic bonding: a randomized clinical trial. European Journal of Orthodontics 2004;26(6):565-71.	
doi.org/10.1093%2Fejo%2F26.6.565		1 approved by the local area dental ethics committee	
2003	Ireland 2003	Ireland AJ, Knight H, Sherriff M. An in vivo investigation into bond failure rates with a new self-etching primer system. American Journal of Orthodontics and Dentofacial Orthopedics 2004;124(3):323-6.	
doi.org/10.1016%2FS0889-5406%2803%2900403-7		1 approved by the local ethics committee	

2002 Asgari 2002 Asgari S, Salas A, English J, Powers J. Clinical evaluation of bond failure rates with a new self-etching primer. Journal of Clinical Orthodontics 2002;36(12):687-9.

ncbi.nlm.nih.gov/pubmed/12572254

--- (article has not been accessed online) ---

Hua F, Xie H, Worthington HV, Furness S, Zhang Q, Li C http://doi.org/10.1002/14651858.CD008367.pub3

2016 Oral hygiene care for critically ill patients to prevent ventilator-associated pneumonia Primary studies: 38

2016 Mo 2016 Mo ZD, Li XL, Ke JY, Wu JP, Chen XW. Analysis of risk factors in ventilator-associated pneumonia and preventive effect of oral care. Chinese Journal of Nosocomiology 2016;26(3):698-699, 705.

en.cnki.com.cn/Article_en/CJFDTotal-ZHYY201603078.htm

--- (article has not been accessed online) ---

2014 Seguin 2014 Seguin P, Laviolle B, Dahyot-Fizelier C, Dumont R, Veber B, Gergaud S, et al. Effect of oropharyngeal povidone-iodine preventive oral care on ventilator-associated pneumonia in severely brain-injured or cerebral hemorrhage patients: a multicenter, randomized controlled trial. Critical Care Medicine 2014;42:1-8.

doi.org/10.1097%2FCCM.0b013e3182a2770f

2 approved by the Consultative Committee for the Protection of People in Biomedical Research of Rennes, (Protocol no. 07/34–651).

2013 Berry 2013 Berry A M. A comparison of Listerine and sodium bicarbonate oral cleansing solutions on dental plaque colonisation and incidence of ventilator associated pneumonia in mechanically ventilated patients: a randomised control trial. Intensive & critical care nursing 2013;29(5):275-81.

doi.org/10.1016%2Fj.iccn.2013.01.002

1 approved by the Ethics Committee of a 1000 bed tertiary referral teaching hospita

2013 Stefanescu 201 Stefanescu BM, Hétu C, Slaughter JC, O'Shea TM, Shetty AK. A pilot study of Biotene OralBalance gel for oral care in mechanically ventilated preterm neonates. Contemporary Clinical Trials 2013;35(2):33-9.

doi.org/10.1016%2Fj.cct.2013.03.010

1 approved by the Institutional Review Boards of the Wake Forest School of Medicine and Forsyth Medical Center, Winston-Salem, North Carolina

2013 Tang 2013 Tang J, Chen SL, Deng JL. Efficacy of mouth cavity irrigation in prevention of ventilator-associated pneumonia. Chinese Journal of Nosocomiology 2013;23(17):4119-21.

en.cnki.com.cn/Article_en/CJFDTotal-ZHYY201317018.htm

--- (article has not been accessed online) ---

2012 Feng 2012 Feng S, Sun X, Chen Y. Application of different mouthwashes in oral nursing for patients with orotracheal intubation. China Medicine and Pharmacy 2012;8(2):100-1.

--- (article has not been accessed online) ---

2012 Kushara 2012a Kusahara DM, Peterlini MA, Pedreira ML. Oral care with 0.12% chlorhexidine for the prevention of ventilator-associated pneumonia in critically ill children: Randomised, controlled and double blind trial. International Journal of Nursing Studies 2012;49(11):1354-63.

doi.org/10.1016%2Fj.ijnurstu.2012.06.005

1 approved by the institution's Research Ethics Committee

2012	Long 2012		Long Y, Mou G, Zuo Y, lv F, Feng Q, Du J. Effect of modified oral nursing method on the patients with orotracheal intubation. Journal of Nurses Training 2012;27(24):2290-3.<br>--- (article has not been accessed online) ---
2012	Lorente 2012	doi.org/10.1007%2Fs10096-012-1605-y	Lorente L, Lecuona M, Jimenez A, Palmero S, Pastor E, Lafuente N, et al. Ventilator-associated pneumonia with or without toothbrushing: a randomized controlled trial. European Journal of Clinical Microbiology and Infectious Diseases 2012;31(10):2621-9.<br>1 approved by the Institutional Review Board
2012	Meinberg 2012	doi.org/10.1590%2FS0103-507X2012000400013	Meinberg MC, Cheade M de F, Miranda AL, Fachini MM, Lobo SM. The use of 2% chlorhexidine gel and toothbrushing for oral hygiene of patients receiving mechanical ventilation: effects on ventilator-associated pneumonia [Uso de clorexidina 2% gel e escovacao mecanica na higiene bucal de pacientes sob ventilacao mecanica: efeitos na pneumonia associada a ventilador]. Revista Brasileira de Terapia Intensiva 2012;24(4):369-74<br>1 approved by the Research Ethics Committee at the Faculdade de Medicina de São José do Rio Preto
2012	Ozcaka 2012	doi.org/10.1111/j.1600-0765.2012.01470.x	Özçaka O, Basoglu OK, Buduneli N, Tasbakan MS, Bacakoglu F, Kinane DF. Chlorhexidine decreases the risk of ventilator-associated pneumonia in intensive care unit patients: a randomized clinical trial. Journal of Periodontal Research 2012;47(5):584-92.<br>1 approved by the local Ethics Committee
2012	Predenergast 2	doi.org/10.1007%2Fs12028-011-9502-2	Prendergast V, Jakobsson U, Renvert S, Hallberg IR. Effects of a standard versus comprehensive oral care protocol among intubated neuroscience ICU patients: results of a randomized controlled trial. Journal of Neuroscience Nursing 2012;44(3):134-46.<br>1 approved by the Institutional Review Board of St. Joseph's Hospital and Medical Center, Phoenix, Arizona
2012	Sebastian 2012	doi.org/10.1097%2FPCC.0b013e31824ea119	Sebastian MR, Lodha R, Kapil A, Kabra SK. Oral mucosal decontamination with chlorhexidine for the prevention of ventilator-associated pneumonia in children - a randomized, controlled trial. Pediatric Critical Care Medicine 2012;13(5):e305-10.<br>1 approved by the institutional ethics committe
2012	Zhao 2012	en.cnki.com.cn/Article_en/CJFDTotal-ZHYY201223024.htm	Zhao Y. Research on application of Yikou gargle in prevention of ventilation associated pneumonia. Chinese Journal of Nosocomiology 2012;23(22):5232-3.<br>--- (article has not been accessed online) ---
2011	Berry 2011	doi.org/10.1016%2Fj.ijnurstu.2010.11.004	Berry AM, Davidson PM, Masters J, Rolls K, Ollerton R. Effects of three approaches to standardized oral hygiene to reduce bacterial colonization and ventilator associated pneumonia in mechanically ventilated patients: A randomised control trial. International Journal of Nursing Studies 2011;48(6):681-8.<br>1 approved by both a hospital and a university institutional review boards

Year	Study	Reference / Ethics
2011	Grap 2011<br>doi.org/10.1016%2Fj.hrtlng.2011.01.006	Grap MJ, Munro CL, Hamilton VA, Elswick RK Jr, Sessler CN, Ward KR. Early, single chlorhexidine application reduces ventilator-associated pneumonia in trauma patients. Heart & Lung 2011;40(5):e115-22.<br>0 Ethics committee not mentioned
2011	Jacomo 2011<br>doi.org/10.1086%2F660018	Jacomo AD, Carmona F, Matsuno AK, Manso PH, Carlotti AP. Effect of oral hygiene with 0.12% chlorhexidine gluconate on the incidence of nosocomial pneumonia in children undergoing cardiac surgery. Infection Control & Hospital Epidemiology 2011;32(6):591-6.<br>--- (article has not been accessed online) ---
2011	Roca Biosca 201<br>doi.org/10.1378/chest.09-0706.	Roca Biosca A, Anguera Saperas L, García Grau N, Rubio Rico L, Velasco Guillén MC. Prevention of mechanical ventilator-associated pneumonia: a comparison of two different oral hygiene methods. Enfermería Intensiva 2011;22(3):104-11/Chest 136 (2009) 433–439.<br>1 approved by the Institutional Ethics Board
2011	Yao 2011<br>doi.org/10.1097%2FJNR.0b013e318236d05f	Yao LY, Chang CK, Maa SH, Wang C, Chen CC. Brushing teeth with purified water to reduce ventilator-associated pneumonia. Journal of Nursing Research 2011;19(4):289-97.<br>2 approved by the institutional ethics board of the study hospital in northern Taiwan
2010	Cabov 2010<br>doi.org/10.1097%2FCCM.0b013e3181930026	Cabov T, Macan D, Husedzinovic I, Skrlin-Subic J, Bosnjak D, Sestan-Crnek S, et al. The impact of oral health and 0.2% chlorhexidine oral gel on the prevalence of nosocomial infections in surgical intensive-care patients: a randomized placebo-controlled study. Wiener Klinische Wochenschrift 2010;122(13-14):397-404.<br>1 approved by the Institutional Ethics Board of the University Hospital Dubrava and the School of Dental Medicine in Zagreb
2009	Bellissimo-Rodri<br>doi.org/10.1086%2F605722	Bellissimo-Rodrigues F, Bellissimo-Rodrigues WT, Viana JM, Teixeira GC, Nicolini E, Auxiliadora-Martins M, et al. Effectiveness of oral rinse with chlorhexidine in preventing nosocomial respiratory tract infections among intensive care unit patients. Infection Control & Hospital Epidemiology 2009;30(10):952-8.<br>--- (article has not been accessed online) ---
2009	Caruso 2009<br>doi.org/10.1097%2FCCM.0b013e3181930026	Caruso P, Denari S, Ruiz SAL, Demarzo SE, Deheinzelin D. Saline instillation before tracheal suctioning decreases the incidence of ventilator-associated pneumonia. Critical Care Medicine 2009;37(1):32-8.<br>1 approved by the hospital ethics committee
2009	Hu 2009	Hu X, Chen X. Application of improved oral nursing method to orotracheal intubation. Chinese Journal of Misdiagnostics 2009;9(17):4058-9.<br>--- (article has not been accessed online) ---

Cochrane_Author		Primary studies_	doi
2009	Munro 2009	Munro CL, Grap MJ, Jones DJ, McClish DK, Sessler CN. Chlorhexidine, toothbrushing, and preventing ventilator-associated pneumonia in critically ill adults. American Journal of Critical Care 2009;18(5):428-37.	doi.org/10.4037%2Fajcc2009792
		1 approved by the Office of Research Subjects Protection of Virginia Commonwealth University, Richmond, Virginia	
2009	Panchabhai 200	Panchabhai TS, Dangayach NS, Krishnan A, Kothari VM, Karnad DR. Oropharyngeal cleansing with 0.2% chlorhexidine for prevention of nosocomial pneumonia in critically ill patients: an open-label randomized trial with 0.01% potassium permanganate as control. Chest 2009;135(5):1150-6.	doi.org/10.1378%2Fchest.08-1321
		--- (article has not been accessed online) ---	
2009	Pobo 2009	Pobo A, Lisboa T, Rodriguez A, Sole R, Magret M, Trefler S, et al. A randomized trial of dental brushing for preventing ventilator-associated pneumonia. Chest 2009;136(2):433-9.	doi.org/10.1378%2Fchest.09-0706
		1 approved by the Institutional Ethics Board	
2009	scannpieco 200	Scannapieco FA, Yu J, Raghavendran K, Vacanti A, Owens SI, Wood K, et al. A randomized trial of chlorhexidine gluconate on oral bacterial pathogens in mechanically ventilated patients. Critical Care 2009;13(4):R117.	doi.org/10.1186%2Fcc7967
		1 approved by the University at Buffalo Institutional Review Board	
2008	Chen 2008	Chen QL, Ye XF, Jiang YZ, Yan MQ. Application of new oral care method to orotracheal intubation. Fujian Medical Journal 2008;30(5):155-7.	doi.org/10.1378%2Fchest.109.6.1556
		--- (article has not been accessed online) ---	
2008	Fields 2008	Fields LB. Oral care intervention to reduce incidence of ventilator-associated pneumonia in the neurologic intensive care unit. Journal of Neuroscience Nursing 2008;40(5):291-8.	doi.org/10.1097%2F01376517-200810000-00007
		1 approved by the hospital's institutional review board (IRB)	
2008	Tantipong 2008	Tantipong H, Morkchareonpong C, Jaiyindee S, Thamlikitkul V. Randomized controlled trial and meta-analysis of oral decontamination with 2% chlorhexidine solution for the prevention of ventilator-associated pneumonia. Infection Control & Hospital Epidemiology 2008;29(2):131-6.	doi.org/10.1086%2F526438
		--- (article has not been accessed online) ---	
2008	Xu 2008	Xu HL. Application of improved oral nursing method to the prevention of ventilator-associated pneumonia. Journal of Qilu Nursing 2008;14(19):15-6.	
		--- (article has not been accessed online) ---	

2007	Xu 2007		Xu J, Feng B, He L, Shen H, Chen XY. Influence of different oral nursing methods on ventilator-associated pneumonia and oral infection in the patients undergoing mechanical ventilation. Journal of Nursing Science 2007;7(22):56-7.
			--- (article has not been accessed online) ---
2006	Bopp 2006	ncbi.nlm.nih.gov/pubmed/16953990	Bopp M, Darby M, Loftin KC, Broscious S. Effects of daily oral care with 0.12% chlorhexidine gluconate and a standard oral care protocol on the development of nosocomial pneumonia in intubated patients: a pilot study. Journal of Dental Hygiene 2006;80(3):9.
			--- (article has not been accessed online) ---
2006	Koeman 2006	doi.org/10.1164%2Frccm.200505-820OC	Koeman M, Van der Ven AJ, Hak E, Joore HC, Kaasjager K, De Smet AG, et al. Oral decontamination with chlorhexidine reduces the incidence of ventilator-associated pneumonia. American Journal of Respiratory & Critical Care Medicine 2006;173(12):1348-55.
			1 approved by the institutional review board committee of each participating hospital
2006	Seguin 2006	doi.org/10.1097%2F01.CCM.0000214516.73076.82	Seguin P, Tanguy M, Laviolle B, Tirel O, Malledant Y. Effect of oropharyngeal decontamination by povidone-iodine on ventilator-associated pneumonia in patients with head trauma. Critical Care Medicine 2006;34(5):1514-9.
			1 approved by the IRB for human research at our hospital (Comité Consultatif de Protection des Personnes dans la Recherche Biomedicale de Rennes)
2005	Fourrier 2005	doi.org/10.1097%2F01.CCM.0000171537.03493.B0	Fourrier F, Dubois D, Pronnier P, Herbecq P, Leroy O, Desmettre T, et al. Effect of gingival and dental plaque antiseptic decontamination on nosocomial infections acquired in the intensive care unit: a double-blind placebo-controlled multicenter study. Critical Care Medicine 2005;33(8):1728-35.
			1 approved by the institutional scientific and ethics committee of our university hospital
2000	Fourrier 2000	doi.org/10.1007%2Fs001340000585	Fourrier F, Cau-Pottier E, Boutigny H, Roussel-Delvallez M, Jourdain M, Chopin C. Effects of dental plaque antiseptic decontamination on bacterial colonization and nosocomial infections in critically ill patients. Intensive Care Medicine 2000;26(9):1239-47.
			1 approved by the Institutional ethics board of our hospital
1996	DeRiso 1996	doi.org/10.1378%2Fchest.109.6.1556	DeRiso AJ 2nd, Ladowski JS, Dillon TA, Justice JW, Peterson AC. Chlorhexidine gluconate 0.12% oral rinse reduces the incidence of total nosocomial respiratory infection and nonprophylactic systemic antibiotic use in patients undergoing heart surgery. Chest 1996;109(6):1556-61.
			1 approved by the Institutional Review Board of Lutheran Hospital of Indiana

Iheozor-Ejiofor Z, Worthington HV, Walsh T, O'Malley L, Clarkson JE, Macey R http://doi.org/10.1002/14651858.CD010856.pub2

2015 Water fluoridation for the prevention of dental caries Primary studies: 155

EPIDEMIOLOGY EPIDEMIOLOGY REVIEW

Innes NP, Ricketts D, Chong LY, Keightley AJ, Lamont T, Santamaria RM http://doi.org/10.1002/14651858.CD005512.pub3

2015 Preformed crowns for decayed primary molar teeth Primary studies: 5

2014 Santemaria 201 Santamaria RM, Innes NP, Machiulskiene V, Evans DJ, Splieth CH. Caries management strategies for primary molars: 1-yr randomized control trial results. Journal of Dental Research 2014;93(11):1062-9.

doi.org/10.1177%2F0022034514550717

2 approved by the Research Ethics Committee of Greifswald University, (BB 39/11;

2012 Hutcheson 201 Hutcheson C, Seale NS, McWhorter A, Kerins C, Wright J. Multi-surface composite vs stainless steel crown restorations after mineral trioxide aggregate pulpotomy: a randomized controlled trial. Pediatric Dentistry 2012;34:460-7.

ingentaconnect.com/contentone/aapd/pd/2012/00000034/[illegible]

1 approved by the the Institutional Review Board at Texas A&M University Health Science Center, Baylor College of Dentistry

2012 Innes 2012 Innes NP, Evans DJ, Stirrups DR. Sealing caries in primary molars: randomized control trial, 5-year results. Journal of Dental Research 2011;90:1405-10

doi.org/10.1177%2F0022034511422064

2 approved by the Tayside Committee on Medical Research Ethics approval 108/00)

2008 Atieh 2008 Atieh M. Stainless steel crown versus modified open-sandwich restorations for primary molars: a 2-year randomized clinical trial. International Journal of Paediatric Dentistry 2008 Sep;18(5):325-32. [PUBMED: 18328050]

doi.org/10.1111/j.1365-263X.2007.00900.x

1 approved by the Health Services at the Saudi Electricity Company

2003 Ram 2003 Ram D, Fuks AB, Eidelman E. Long-term clinical performance of esthetic primary molar crowns. Pediatric Dentistry 2003;25(6):582-4.

ncbi.nlm.nih.gov/pubmed/14733474

--- (article has not been accessed online) ---

Jambi S, Thiruvenkatachari B, O'Brien KD, Walsh T http://doi.org/10.1002/14651858.CD008375.pub2

2013 Orthodontic treatment for distalising upper first molars in children and adolescents Primary studies: 10

2011 Armi 2011 Armi P, Cozza P, Baccetti T. Effect of RME and headgear treatment on the eruption of palatally displaced canines: a randomized clinical study. The Angle Orthodontist 2011;81(3):370-4.

doi.org/10.2319%2F062210-339.1

1 approved by the Ethical Committee at the University of Florence and Rome.

2010 Acar 2010 Acar AG, Gursoy S, Dincer M. Molar distalization with a pendulum appliance K-loop combination. European Journal of Orthodontics 2010;32(4):459-65.

doi.org/10.1093%2Fejo%2Fcjp136

1 approved by the ethics committee of Başkent University

Year	Study	Primary study / Ethics	doi
2010	Papadopoulos 2	Papadopoulos MA, Melkos AB, Athanasiou AE. Noncompliance maxillary molar distalization with the First Class appliance: A randomized controlled trial. American Journal of Orthodontics and Dentofacial Orthopedics 2010;137(5):586.e1-13.<br>1 approved by the ethical committee of the School of Dentistry of the Aristotle University of thessaloniki	doi.org/10.1016%2Fj.ajodo.2009.10.033
2008	Altug-Atac 2008	Altug-Atac AT, Erdem D, Arat ZM. Three-dimensional bimetric maxillary distalization arches compared with a modified Begg intraoral distalization system. European Journal of Orthodontics 2008;30(1):73-9.<br>0 Ethics committee not mentioned	doi.org/10.1093%2Fejo%2Fcjm083
2008	Baccetti 2008	Baccetti T, Leonardo M, Armi P. A randomized clinical study of two interceptive approaches to palatally displaced canines. European Journal of Orthodontics 2008;30:381-5.<br>1 approved by the Ethical Committee at the University of Florence	doi.org/10.1093%2Fejo%2Fcjn023
2007	De Oliveira 200	de Oliveira Jr JN, de Almeida RR, de Almeida MR, de Oliveira JN. Dentoskeletal changes induced by the Jasper jumper and cervical headgear appliances followed by fixed orthodontic treatment. American Journal of Orthodontics and Dentofacial Orthopedics 2007;132:54-62.<br>0 Ethics committee not mentioned	doi.org/10.1016%2Fj.ajodo.2005.07.028
2006	Karacay 2006	Karacay S, Akin E, Olmez H, Gurton AU, Sagdic D. Forsus Nitinol flat spring and Jasper Jumper corrections of Class II division 1 malocclusions. The Angle Orthodontist 2006;76(4):666-72.<br>1 approved by ethics committee of Gulhane Military Medical Academy	angle.org/doi/10.1043/0003-3219(2006)076[0666:FNFSAJ]2.0.CO;2
2005	Bondemark 200	Bondemark L, Karlsson I. Extraoral vs intraoral appliance for distal movement of maxillary first molars: a randomized controlled trial. The Angle Orthodontist 2005;75(5):699-706.<br>1 approved by The ethic committee of Lund/Malmö¨ University, Sweden	angle.org/doi/10.1043/0003-3219(2005)75[699:EVIAFD]2.0.CO;2

Jambi S, Walsh T, Sandler J, Benson PE, Skeggs RM, O'Brien KD http://doi.org/10.1002/14651858.CD005098.pub3

2014 Reinforcement of anchorage during orthodontic brace treatment with implants or other surgical methods Primary studies: 15

Year	Study	Primary study / Ethics	doi
2011	Toy 2011	Toy E, Enacar A. The effects of the pendulum distalising appliance and cervical headgear on the dentofacial structures. Australian Orthodontic Journal 2011;27(1):10-6.<br>--- (article has not been accessed online) ---	ncbi.nlm.nih.gov/pubmed/21696108
2002	Paul 2002	Paul LD, O'Brien KD, Mandall NA. Upper removable appliance or Jones Jig for distalizing first molars? A randomized clinical trial. Orthodontics and Craniofacial Research 2002;5(4):238-42.<br>0 Ethics committee not mentioned	doi.org/10.1034/j.1600-0544.2002.01169.x

James P, Worthington HV, Parnell C, Harding M, Lamont T, Cheung A, Whelto http://doi.org/10.1002/14651858.CD008676.pub2

2017 Chlorhexidine mouthrinse as an adjunctive treatment for gingival health Primary studies: 51

Cochrane_Author		Primary studies_	doi
2015	Graziani 2015	Graziani F, Gabriele M, D'Aiuto F, Suvan J, Tonelli M, Cei S. Dental plaque, gingival inflammation and tooth -discolouration with different commercial -formulations of 0.2% chlorhexidine rinse: a double-blind randomised controlled clinical trial. Oral Health and Preventive Dentistry 2015;13(2):101-11.	doi.org/10.3290/j.ohpd.a32827
		1 approved by the ethics committe of the university hospital of Pisa (Italy)	
2015	Jose 2015	Jose A, Butler A, Payne D, Maclure R, Rimmer P, Bosma ML. A randomised clinical study to evaluate the efficacy of alcohol-free or alcohol-containing mouthrinses with chlorhexidine on gingival bleeding. British Dental Journal 2015;219(3):125-30, 6.	doi.org/10.1038%2Fsj.bdj.2015.592
		1 approved by an independent ethics committee	
2015	Zimmer 2015	Zimmer S, Korte P, Verde P, Ohmann C, Naumova E, Jordan RA. Randomized controlled trial on the efficacy of new alcohol-free chlorhexidine mouthrinses after 8 weeks. International Journal of Dental Hygiene 2015;13(2):110-6.	doi.org/10.1111/idh.12111
		2 approved by the ethical reviewboard of the Witten/Herdecke University (Approval# 22/2010)	
2014	Anauate-Netto	Anauate-Netto C, Anido-Anido A, Leegoy HR, Matsumoto R, Alonso RC, Marcucci MC, et al. Randomized, double-blind, placebo-controlled clinical trial on the effects of propolis and chlorhexidine mouthrinses on gingivitis. Brazilian Dental Science 2014;17(1):11-5.	ncbi.nlm.nih.gov/pubmed/24971228
		2 approved by the Institutional Review Board (UNIBAN-Protocol N.0038/2007),	
2014	Bhat 2014	Bhat N, Mitra R, Oza S, Mantu Vinayak K, Bishnoi S, Gohil M, et al. The antiplaque effect of herbal mouthwash in comparison to chlorhexidine in human gingival disease: a randomized placebo controlled clinical trial. Journal of Complementary and Integrative Medicine 2014;11(2):129-37.	doi.org/10.1515%2Fjcim-2014-0002
		--- (article has not been accessed online) ---	
2012	Feres 2012	Feres M, Soares GM, Mendes JA, Silva MP, Faveri M, Teles R, et al. Metronidazole alone or with amoxicillin as adjuncts to non-surgical treatment of chronic periodontitis: a 1-year double-blinded, placebo-controlled, randomized clinical trial. Journal of Clinical Periodontology 2012;39(12):1149-58.	doi.org/10.1111/jcpe.12004
		1 approved by the Guarulhos University Clinical Research Ethics Committee	
2012	Lopez-Jornet 20	Lopez-Jornet P, Plana-Ramon E, Leston JS, Pons-Fuster A. Short-term side effects of 0.2% alcohol-free chlorhexidine mouthrinse in geriatric patients: a randomized, double-blind, placebo-controlled study. Gerodontology 2012; Vol. 29, issue 4:292-8.	doi.org/10.1111/j.1741-2358.2012.00671.x
		1 approved by the local Ethics Committee of the University of Murcia.	
2011	Bajaj 2011	Bajaj N, Tandon S. The effect of Triphala and Chlorhexidine mouthwash on dental plaque, gingival inflammation, and microbial growth. International Journal of Ayurveda Research 2011;2(1):29-36.	doi.org/10.4103%2F0974-7788.83188
		--- (article has not been accessed online) ---	
2011	Pereira 2011	Pereira SL, de Oliveira JW, Angelo KK, da Costa AM, Costa F. Clinical effect of a mouth rinse containing Ocimum gratissimum on plaque and gingivitis control. Journal of Contemporary Dental Practice 2011;12:350-5.	ncbi.nlm.nih.gov/pubmed/22269195
		2 approved by the Institutional Ethics Committee (Report Cotica/Unifor: 161/ 2009)	

Cochrane_Author		Primary studies_	doi
2009	Feres 2009	Feres M, Gursky LC, Faveri M, Tsuzuki CO, Figueiredo LC. Clinical and microbiological benefits of strict supragingival plaque control as part of the active phase of periodontal therapy. Journal of Clinical Periodontology 2009; Vol. 36, issue 10:857-67.	doi.org/10.1111/j.1600-051X.2009.01471.x
		1 approved by Guarulhos University's Ethics Committee in Clinical Research	
2009	Turkoglu 2009	Turkoglu O, Becerik S, Emingil G, Kutukculer N, Baylas H, Atilla G. The effect of adjunctive chlorhexidine mouthrinse on clinical parameters and gingival crevicular fluid cytokine levels in untreated plaque-associated gingivitis. Inflammation Research 2009; Vol. 58, issue 5:277-83.	doi.org/10.1007/s00011-008-8129-z
		1 approved by the ethics committee of the Ege University School of Medicine	
2008	Van Strydonck	Van Strydonck DA, Timmerman MF, Van der Velden U, Van der Weijden F. Clinical efficacy of a chlorhexidine-delivering toothbrush. Journal of Clinical Periodontology 2008; Vol. 35, issue 7:584-90.	doi.org/10.1111/j.1600-051X.2008.01227.x
		2 approved by the MedicalEthic Committee of the Amsterdam Medical Center (MEC 98/139)	
2007	Jayaprakash 20	Jayaprakash K, Veeresha KL, Hiremath SS. A comparative study of two mouthrinses on plaque and gingivitis in school children in the age group of 13-16 years in Bangalore city. Journal of the Indian Society of Pedodontics and Preventive Dentistry 2007;25(3):126-9.	doi.org/10.4103%2F0970-4388.36562
		1 approved by the Ethical committee of the Government Dental College, Bangalor	
2006	Faveri 2006	Faveri M, Gursky LC, Feres M, Shibli JA, Salvador SL, de Figueiredo LC. Scaling and root planing and chlorhexidine mouthrinses in the treatment of chronic periodontitis: a randomized, placebo-controlled clinical trial. Journal of Clinical Periodontology 2006; Vol. 33, issue 11:819-28.	doi.org/10.1111/j.1600-051X.2006.00994.x
		1 approved by Ethics Committee	
2006	Rahmani 2006	Rahmani ME, Radvar M. Effects of chlorhexidine (0.2%) as irrigant during ultrasonic debridement: A clinical study. International Journal of Pharmacology 2006; Vol. 2, issue 1:89-92.	
		--- (article has not been accessed online) ---	
2006	Southern 2006	Southern EN, McCombs GB, Tolle SL, Marinak K. The comparative effects of 0.12% chlorhexidine and herbal oral rinse on dental plaque-induced gingivitis. Journal of Dental Hygiene 2006; Vol. 80, issue 1:12.	ncbi.nlm.nih.gov/pubmed/16451766
		1 approved by the Old Dominion University Institutional Review Board.	
2006	Zimmer 2006	Zimmer S, Kolbe C, Kaiser G, Krage T, Ommerborn M, Barthel C. Clinical efficacy of flossing versus use of antimicrobial rinses. Journal of Periodontology 2006; Vol. 77, issue 8:1380-5.	doi.org/10.1902/jop.2006.050362
		1 approved by the ethics committee of the Medical Faculty, Heinrich-Heine-University	
2005	Ernst 2005	Ernst CP, Canbek K, Dillenburger A, Willershausen B. Clinical study on the effectiveness and side effects of hexetidine and chlorhexidine mouthrinses versus a negative control. Quintessence International 2005; Vol. 36, issue 8:641-52.	quintpub.com/journals/qi/fulltext.php?article_id=1938
		0 Ethics committee not mentioned	

Cochrane_Author	Primary studies_	doi
2005 Stookey 2005	Stookey GK, Beiswanger B, Mau M, Isaacs RL, Witt JJ, Gibb R. A 6-month clinical study assessing the safety and efficacy of two cetylpyridinium chloride mouthrinses. American Journal of Dentistry 2005;18(Spec No):24A-8A.	amjdent.com/Archive/2005/July%2005%20SI%20-%20Press.pdf
	1 approved by the Institutional Ethics-Review Board	
2004 Charles 2004	Charles CH, Mostler KM, Bartels LL, Mankodi SM. Comparative antiplaque and antigingivitis effectiveness of a chlorhexidine and an essential oil mouthrinse: 6-month clinical trial. Journal of Clinical Periodontology 2004; Vol. 31, issue 10:878-84.	doi.org/10.1111/j.1600-051X.2004.00578.x
	1 approved by the Institu-tional Review Board of the CorningBesselaar Clinical Research Unit	
2001 Hoffmann 2001	Hoffmann T, Bruhn G, Richter S, Netuschil L, Brecx M. Clinical controlled study on plaque and gingivitis reduction under long-term use of low-dose chlorhexidine solutions in a population exhibiting good oral hygiene. Clinical Oral Investigations 2001; Vol. 5, issue 2:89-95.	doi.org/10.1007/s007840100114
	2 approved by the Medical Faculty of the University of Technology, Dresden, agreed to the study protocol (EK 890999).	
1999 Lucas 1999	Lucas GQ, Lucas ON. Preventive action of short-term and long-term chlorhexidine rinses. Acta Odontologica Latinoamericana 1999; Vol. 12, issue 1:45-58.	
	--- (article has not been accessed online) ---	
1998 Hase 1998	Hase JC, Attstrom R, Edwardsson S, Kelty E, Kisch J. 6-month use of 0.2% delmopinol hydrochloride in comparison with 0.2% chlorhexidine digluconate and placebo. (I). Effect on plaque formation and gingivitis. Journal of Clinical Periodontology 1998; Vol. 25, issue 9:746-53.	doi.org/10.1111/j.1600-051X.1998.tb02516.x
	1 approved by theSwedish National Board of Heallh and Welfare, and the Ethics Committee of the Faculty of Medicine, Lund University	
1998 Lang 1998	Lang NP, Hase JC, Grassi M, Hammerle CH, Weigel C, Kelty E, et al. Plaque formation and gingivitis after supervised mouthrinsing with 0.2% delmopinol hydrochloride, 0.2% chlorhexidine digluconate and placebo for 6 months. Oral Diseases 1998; Vol. 4, issue 2:105-13.	
	--- (article has not been accessed online) ---	
1998 Navarro 1998	Navarro D, Santos EAT, Rocha JCF, Bremm LL, Jukoski M, Ribeiro PG, et al. Effect of chlorhexidine digluconate, Plantago major and placebo mouth rinse on dental plaque and gingivitis [Efeitos do digluconato de clorexidina, Plantago major e placebo sobre placa dental e gengivite: uma comparacao clinica da eficacia de colutorios]. Revista Brasileira de Plantas Medicinais 1998;1(1):28.	
	--- (article has not been accessed online) ---	
1997 Anderson 1997	Anderson GB, Bowden J, Morrison EC, Caffesse RG. Clinical effects of chlorhexidine mouthwashes on patients undergoing orthodontic treatment. American Journal of Orthodontics and Dentofacial Orthopedics 1997;111(6):606-12.	doi.org/10.1016%2FS0889-5406%2897%2970312-3
	1 approved by the Committee for the Protection of Human Subjects of the institution	
1997 Corbet 1997	Corbet EF, Tam JO, Zee KY, Wong MC, Lo EC, Mombelli AW, et al. Therapeutic effects of supervised chlorhexidine mouthrinses on untreated gingivitis. Oral Diseases 1997; Vol. 3, issue 1:9-18.	
	--- (article has not been accessed online) ---	

Cochrane_Author		Primary studies_	doi
1997	Eaton 1997	Eaton KA, Rimini FM, Zak E, Brookman DJ, Hopkins LM, Cannell PJ, et al. The effects of a 0.12% chlorhexidine-digluconate-containing mouthrinse versus a placebo on plaque and gingival inflammation over a 3-month period. A multicentre study carried out in general dental practices. Journal of Clinical Periodontology 1997; Vol. 24, issue 3:189-97.	doi.org/10.1111/j.1600-051X.1997.tb00490.x
		1 approved by the Joint Researchand Ethics Committee of the EastmanDental Institute and Hospital and fromthe five Local Ethics Comitittees	
1995	Hase 1995	Hase JC, Ainamo J, Etemadzadeh H, Astrom M. Plaque formation and gingivitis after mouthrinsing with 0.2% delmopinol hydrochloride, 0.2% chlorhexidine digluconate and placebo for 4 weeks, following an initial professional tooth cleaning. Journal of Clinical Periodontology 1995; Vol. 22, issue 7:533-9.	doi.org/10.1111/j.1600-051X.1995.tb00801.x
		--- (article has not been accessed online) ---	
1994	Chaves 1994	Chaves ES, Kornman KS, Manwell MA, Jones AA, Newbold DA, Wood RC. Mechanism of irrigation effects on gingivitis. Journal of Periodontology 1994; Vol. 65, issue 11:1016-21.	doi.org/10.1902/jop.1994.65.11.1016
		0 Ethics committee not mentioned	
1994	Sanz 1994	Sanz M, Vallcorba N, Fabregues S, Muller I, Herkstroter F. The effect of a dentifrice containing chlorhexidine and zinc on plaque, gingivitis, calculus and tooth staining. Journal of Clinical Periodontology 1994; Vol. 21, issue 6:431-7.	doi.org/10.1111/j.1600-051X.1994.tb00741.x
		--- (article has not been accessed online) ---	
1993	Brecx 1993	Brecx M, Macdonald LL, Legary K, Cheang M, Forgay MG. Long-term effects of Meridol and chlorhexidine mouthrinses on plaque, gingivitis, staining, and bacterial vitality. Journal of Dental Research 1993; Vol. 72, issue 8:1194-7.	doi.org/10.1177/00220345930720080601
		--- (article has not been accessed online) ---	
1993	Jenkins 1993	Jenkins S, Addy M, Newcombe R. Evaluation of a mouthrinse containing chlorhexidine and fluoride as an adjunct to oral hygiene. Journal of Clinical Periodontology 1993; Vol. 20, issue 1:20-5.	doi.org/10.1111/j.1600-051X.1993.tb01754.x
		--- (article has not been accessed online) ---	
1993	Joyston-Bechal	Joyston-Bechal S, Hernaman N. The effect of a mouthrinse containing chlorhexidine and fluoride on plaque and gingival bleeding. Journal of Clinical Periodontology 1993; Vol. 20, issue 1:49-53.	doi.org/10.1111/j.1600-051X.1993.tb01760.x
		--- (article has not been accessed online) ---	
1993	Taller 1993	Taller SH. The effect of baking soda/hydrogen peroxide dentifrice (Mentadent) and a 0.12 percent chlorhexidine gluconate mouthrinse (Peridex) in reducing gingival bleeding. Journal of the New Jersey Dental Association 1993; Vol. 64, issue 4:23-5.	
		--- (article has not been accessed online) ---	
1992	Emling 1992	Emling RC, Levin S, Shi X, Weinberg S, Yankell SL. Rembrandt toothpaste stain prevention with and without the use of Peridex. Journal of Clinical Dentistry 1992; Vol. 3, issue 2:59-65. Ernst 2005 {published data only}	
		--- (article has not been accessed online) ---	

Cochrane_Author		Primary studies_	doi
1992	Weitz 1992	Weitz M, Brownstein C, Deasy M. Effect of a twice daily 0.12% chlorhexidine rinse on the oral health of a geriatric population. Clinical Preventive Dentistry 1992; Vol. 14, issue 3:9-13.	
		--- (article has not been accessed online) ---	
1991	Brightman 1991	Brightman LJ, Terezhalmy GT, Greenwell H, Jacobs M, Enlow DH. The effects of a 0.12% chlorhexidine gluconate mouthrinse on orthodontic patients aged 11 through 17 with established gingivitis. American Journal of Orthodontics and Dentofacial Orthopedics 1991;100(4):324-9.	
	doi.org/10.1016%2F0889-5406%2891%2970069-9	0 Ethics committee not mentioned	
1990	Flemmig 1990	Flemmig TF, Newman MG, Doherty FM, Grossman E, Meckel AH, Bakdash MB. Supragingival irrigation with 0.06% chlorhexidine in naturally occurring gingivitis. I. 6 month clinical observations. Journal of Periodontology 1990; Vol. 61, issue 2:112-7.	
	doi.org/10.1902/jop.1990.61.2.112	0 Ethics committee not mentioned	
1990	Overholser 199	Overholser CD, Meiller TF, DePaola LG, Minah GE, Niehaus C. Comparative effects of 2 chemotherapeutic mouthrinses on the development of supragingival dental plaque and gingivitis. Journal of Clinical Periodontology 1990; Vol. 17, issue 8:575-9.	
	doi.org/10.1111/j.1600-051X.1993.tb00774.x	--- (article has not been accessed online) ---	
1989	Banting 1989	Banting D, Bosma M, Bollmer B. Clinical effectiveness of a 0.12% chlorhexidine mouthrinse over two years. Journal of Dental Research 1989;68(Spec Iss):1716-8.	
		--- (article has not been accessed online) ---	
1989	Grossman 1989	Grossman E, Meckel AH, Isaacs RL, Ferretti GA, Sturzenberger OP, Bollmer BW, et al. A clinical comparison of antibacterial mouthrinses: effects of chlorhexidine, phenolics, and sanguinarine on dental plaque and gingivitis. Journal of Periodontology 1989; Vol. 60, issue 8:435-40.	
	doi.org/10.1902/jop.1989.60.8.435	0 Ethics committee not mentioned	
1989	Sanz 1989	Sanz M, Newman MG, Anderson L, Matoska W, Otomo-Corgel J, Saltini C. Clinical enhancement of post-periodontal surgical therapy by a 0.12% chlorhexidine gluconate mouthrinse. Journal of Periodontology 1989; Vol. 60, issue 10:570-6.	
	doi.org/10.1902/jop.1989.60.10.570	0 Ethics committee not mentioned	
1988	de la Rosa 1988	de la Rosa M, Sturzenberger OP, Moore DJ. The use of chlorhexidine in the management of gingivitis in children. Journal of Periodontology 1988; Vol. 59, issue 6:387-9.	
		--- (article has not been accessed online) ---	
1988	de la Rosa 1988	de la Rosa M, Sturzenberger OP, Moore DJ. The use of chlorhexidine in the management of gingivitis in children. Journal of Periodontology 1988; Vol. 59, issue 6:387-9.	
		--- (article has not been accessed online) ---	

1987 Axelsson 1987<br>doi.org/10.1111/j.1600-051X.1987.tb00968.x	Axelsson P, Lindhe J. Efficacy of mouthrinses in inhibiting dental plaque and gingivitis in man. Journal of Clinical Periodontology 1987; Vol. 14, issue 4:205-12.<br>--- (article has not been accessed online) ---
1987 Ferretti 1987<br>doi.org/10.14219%2Fjada.archive.1987.0112	Ferretti GA, Ash RC, Brown AT, Largent BM, Kaplan A, Lillich TT. Chlorhexidine for prophylaxis against oral infections and associated complications in patients receiving bone marrow transplants. Journal of the American Dental Association 1987;114(4):461-7.<br>--- (article has not been accessed online) ---
1986 Grossman 1986<br>doi.org/10.1111/j.1600-0765.1986.tb01513.x	Grossman E, Reiter G, Sturzenberger OP. Six-month study of the effects of a chlorhexidine mouthrinse on gingivitis in adults. Journal of Periodontal Research 1986;21(Suppl 16):33-43.<br>0 Ethics committee not mentioned
1986 Segreto 1986<br>doi.org/10.1111/j.1600-0765.1986.tb01512.x	Segreto VA, Collins EM, Beiswanger BB. A comparison of mouthrinses containing two concentrations of chlorhexidine. Journal of Periodontal Research 1986;21(Suppl 16):23-32.<br>0 Ethics committee not mentioned
1985 Fine 1985	Fine PD. A clinical trial to compare the effect of two antiseptic mouthwashes on gingival inflammation. Journal of Hospital Infection 1985; Vol. 6, issue Suppl A:189-93.<br>--- (article has not been accessed online) ---
1972 Flotra 1972<br>doi.org/10.1111/j.1600-0722.1972.tb00258.x	Flotra L, Gjermo P, Rolla G, Waerhaug J. A 4-month study on the effect of chlorhexidine mouth washes on 50 soldiers. Scandinavian Journal of Dental Research 1972; Vol. 80, issue 1:10-7.<br>0 Ethics committee not mentioned

Jian F, Lai W, Furness S, McIntyre GT, Millett DT, Hickman J, Wang Y http://doi.org/10.1002/14651858.CD007859.pub3

2013 Initial arch wires for tooth alignment during orthodontic treatment with fixed appliances Primary studies: 9

2012 Cioffi 2012<br>quintpub.com/journals/qi/fulltext.php?article_id=11716	Cioffi I, Piccolo A, Tagliaferri R, Paduano S, Galeotti A, Martina R. Pain perception following first orthodontic archwire placement--thermoelastic vs superelastic alloys: a randomized controlled trial. Quintessence International 2012; Vol. 43, issue 1:61-9.<br>1 approved by the local ethics committee
2012 Sebastian 2012<br>doi.org/10.2319%2F072111-460.1	Sebastian B. Alignment efficiency of superelastic coaxial nickel-titanium vs superelastic single-stranded nickel-titanium in relieving mandibular anterior crowding: a randomized controlled prospective study. The Angle Orthodontist 2012;82(4):703-8.<br>1 approved by the Institution

2009 Pandis 2009
doi.org/10.1016%2Fj.ajodo.2009.03.030
Pandis N, Polychronopoulou A, Eliades T. Alleviation of mandibular anterior crowding with copper-nickel-titanium vs nickel-titanium wires: a double-blind randomized control trial. American Journal of Orthodontics and Dentofacial Orthopedics 2009;136(2):152-3.
0 Ethics committee not mentioned

1998 Cobb 1998
ncbi.nlm.nih.gov/pubmed/9918641
Cobb NW 3rd, Kula KS, Phillips C, Proffit WR. Efficiency of multi-strand steel, superelastic Ni-Ti and ion-implanted Ni-Ti archwires for initial alignment. Clinical Orthodontics and Research 1998;1(1):12-9.
--- (article has not been accessed online) ---

1998 Evans 1998
doi.org/10.1016%2FS0889-5406%2898%2970234-3
Evans TJ, Jones ML, Newcombe RG. Clinical comparison and performance perspective of three aligning arch wires. American Journal of Orthodontics and Dentofacial Orthopedics 1998;114(1):32-9.
1 approved by the local ethics committee.

1998 Fernandes 1998
doi.org/10.1007%2FBF01299769
Fernandes LM, Øgaard B, Skoglund L. Pain and discomfort experienced after placement of a conventional or a superelastic NiTi aligning archwire. A randomized clinical trial [Schmerzen und Beschwerden nach Eingliederung eines herkömmlichen und eines superelastischen NiTi-Nivellierungsbogens]. Journal of Orofacial Orthopedics 1998:59(6):331-9.
0 Ethics committee not mentioned

1995 West 1995
doi.org/10.1016%2FS0889-5406%2895%2970046-3
West AE, Jones ML, Newcombe RG. Multiflex versus superelastic: a randomized clinical trial of the tooth alignment ability of initial arch wires. American Journal of Orthodontics and Dentofacial Orthopedics 1995;108(5):464-71.
0 Ethics committee not mentioned

1992 Jones 1992
doi.org/10.1016%2F0889-5406%2892%2970054-E
Jones M, Chan C. The pain and discomfort experienced during orthodontic treatment: a randomized controlled clinical trial of two initial aligning arch wires. American Journal of Orthodontics and Dentofacial Orthopedics 1992;102(4):373-81.
1 approved by the Dental Ethics Panel of the University of Wales College of Medicine

1990 O'Brien 1990
doi.org/10.1093%2Fejo%2F12.4.380
O'Brien K, Lewis D, Shaw W, Combe E. A clinical trial of aligning archwires. European Journal of Orthodontics 1990;12(4):380-4.
--- (article has not been accessed online) ---

Kiran Kumar Krishanappa S, Prashanti E, Sumanth KN, Naresh S, Moe S, Aggar http://doi.org/10.1002/14651858.CD011784.pub2

2016 Interventions for treating oro-antral communications and fistulae due to dental procedures Primary studies: 1

2012 Nezafati 2012
doi.org/10.1016%2Fj.ijom.2011.11.011
Nezafati S, Vafaii A, Ghojazadeh M. Comparison of pedicled buccal fat pad flap with buccal flap for closure of oro-antral communication. International Journal of Oral and Maxillofacial Surgery 2012;41(5):624-8
2 approved by the Tabriz University of Medical Sciences with reference number 1001, 23 May 2011.

Kloukos D, Fudalej P, Sequeira-Byron P, Katsaros C http://doi.org/10.1002/14651858.CD010403.pub2

2016 Maxillary distraction osteogenesis versus orthognathic surgery for cleft lip and palate patients Primary studies: 1

2008 Hong Kong Stud Cheung LK, Chua HD, Hägg MB. Cleft maxillary distraction versus orthognathic surgery: clinical morbidities and surgical relapse. Plastic and Reconstructive Surgery 2006;118(4):996-1008.

doi.org/10.1097%2F01.prs.0000232358.31321.ea

1 approved by the research ethics committee of the Faculty of Dentistry, University of Hong Kong

1995 Erbay 1995 Erbay E, Ugur T, Ulgen M. The effects of Frankel's function regulator (FR-4) therapy on the treatment of Angle Class I skeletal anterior open bite malocclusion. American Journal of Orthodontics and Dentofacial Orthopedics 1995;108(1):9-21.

doi.org/10.1016%2FS0889-5406%2895%2970061-7

0 Ethics committee not mentioned

Lentini-Oliveira DA, Carvalho FR, Rodrigues CG, Ye Q, Prado LB, Prado GF, Hu http://doi.org/10.1002/14651858.CD005515.pub3

2014 Orthodontic and orthopaedic treatment for anterior open bite in children Primary studies: 3

2005 Almeida 2005 Almeida RR. A prospective study of the treatment effects of a removable appliance with palatal crib combined with high-pull chincup therapy in anterior open bite patients [Estudo cefalométrico prospectivo do tratamento da mordida aberta anterior utilizando aparelho removível com grade palatina, associada à mentoneira]. [Dissertation]. Brazil: University of Sao Paulo 2005.

--- (article has not been accessed online) ---

1990 Kiliaridis 1990 Kiliaridis S, Egermark I, Thilander B. Anterior open bite treatment with magnets. European Journal of Orthodontics 1990;12(4):447-57.

doi.org/10.1093%2Fejo%2F12.4.447

--- (article has not been accessed online) ---

Li C, Lv Z, Shi Z, Zhu Y, Wu Y, Li L, Iheozor-Ejiofor Z http://doi.org/10.1002/14651858.CD009197.pub2

2014 Periodontal therapy for the management of cardiovascular disease in patients with chronic periodontitis Primary studies: 0

EMPTY Empty Systematic Review

Littlewood SJ, Millett DT, Doubleday B, Bearn DR, Worthington HV http://doi.org/10.1002/14651858.CD002283.pub4

2016 Retention procedures for stabilising tooth position after treatment with orthodontic braces Primary studies: 15

2015 Edman Tynelius Edman Tynelius G, Petrén S, Bondemark L, Lilja-Karlander E. Five-year postretention outcomes of three retention methods - a randomized controlled trial. European Journal of Orthodontics 2015;37(4):345-53.

doi.org/10.1093%2Fejo%2Fcju063

2 approved by the Ethics Committee of Lund/Malmö University, Sweden (LU515-01).

2013 Aslan 2013 Aslan BI, Dincer M, Salmanli I, Qasem MAM. Comparison of the effects of modified and full-coverage thermoplastic retainers on occlusal contacts. Orthodontics: the Art and Practice of Dentofacial Enhancement 2013;14:e198-208.

quintpub.com/journals/wjo/fulltext.php?article_id=13223

0 Ethics committee not mentioned

Cochrane_Author		Primary studies_	doi
2013	O'Rourke	Salehi, P., Najafi, H. Z., & Roeinpeikar, S. M. (2013). Comparison of survival time between two types of orthodontic fixed retainer: a prospective randomized clinical trial. Progress in orthodontics, 14(1), 25.	doi.org/10.1186/2196-1042-14-25
		1 approved by the ethical committee of the Orthodontic Research Center of Shiraz University of Medical Sciences.	
2013	Salehi 2013	Salehi P, Najafi HZ, Roeinpeikar SM. Comparison of survival time between two types of orthodontic fixed retainer: a prospective randomized clinical trial. Progress in Orthodontics 2013;14:25.	doi.org/10.1186%2F2196-1042-14-25
		1 approved by the ethical committee of the Orthodontic Research Center of Shiraz University of Medical Sciences	
2012	Bolla 2012	Bolla E, Cozzani M, Doldo T, Fontana M. Failure evaluation after a 6-year retention period: a comparison between glass fiber- reinforced (GFR) and multistranded bonded retainers. International Orthodontics 2012;10:16-28.	ncbi.nlm.nih.gov/pubmed/22240271
		0 Ethics committee not mentioned	
2011	Kumar 2011	Kumar AG, Bansal A. Effectiveness and acceptability of Essix and Begg retainers: a prospective study. Australian Orthodontic Journal 2011;27:52-6.	ncbi.nlm.nih.gov/pubmed/21696115
		--- (article has not been accessed online) ---	
2011	Sun 2011	Sun J, Yu MY, Liu L, Chen L, Li HW, Zhang L, et al. Survival time comparison between Hawley and clear overlay retainers: a randomized trial. Journal of Dental Research 2011;90:1197-201.	doi.org/10.1177%2F0022034511415274
		2 approved by the ethics committee of West China Stomatology Hospital (project number 200705)	
2010	Shawesh 2010	Shawesh M, Bhatti B, Usmani T, Mandall N. Hawley retainers full or part time? A randomized clinical trial. European Journal of Orthodontics 2010;32:165-70.	doi.org/10.1093%2Fejo%2Fcjp082
		2 approved by the Central Manchester Research Ethics Committee (reference: 03/07/2307	
2010	Thickett 2010	Thickett E, Power S. A randomized clinical trial of thermoplastic retainer wear. European Journal of Orthodontics 2010;32:1-5.	doi.org/10.1093%2Fejo%2Fcjp061
		2 approved by the East Dorset Local Research Ethics Committee (Ref no. 05/Q2201/76	
2007	Gill 2007	Gill DS, Naini FB, Jones A, Tredwin CJ. Part-time versus full-time retainer wear following fixed appliance therapy: a randomized prospective controlled trial. World Journal of Orthodontics 2007;8:300-6.	ncbi.nlm.nih.gov/pubmed/17902334
		--- (article has not been accessed online) ---	
2007	Millett 2007	McDermott P, Field D, Erfida I, Millett DT. Operator and patient experiences with fixed or vacuum formed retainers. Irish Division IADR Conference Abstract 0017. Cork, 2007.	
		--- (article has not been accessed online) ---	

2007	Rowland 2007 doi.org/10.1016%2Fj.ajodo.2006.06.019	Rowland H, Hichens L, Williams A, Hills D, Killingback N, Ewings P, et al. The effectiveness of Hawley and vacuum-formed retainers: a single-center randomized controlled trial. American Journal of Orthodontics and Dentofacial Orthopedics 2007;132:730-7.	2 approved by the Local Research and Ethics Committee at the United Bristol Healthcare Trust (approval number E5421
2006	Rohaya 2006 mda.org.my/mdj/archieve/2006-01/2006-Vol27-No1.pdf	Rohaya MAW, Shahrul Hisham ZA, Doubleday B. Randomised clinical trial: comparing the efficacy of vacuum-formed and Hawley retainers in retaining corrected tooth rotations. Malaysian Dental Journal 2006;27(1):38-44.	0 Ethics committee not mentioned
2002	Rose 2002 quintpub.com/journals/qi/fulltext.php?article_id=5033	Rose E, Frucht S, Jonas IE. Clinical comparison of a multistranded wire and a direct-bonded polyethylene ribbon-reinforced resin composite used for lingual retention. Quintessence International 2002;33(8):579-83.	1 approved by the local ethics committee
1997	Artun 1997 doi.org/10.1093%2Fejo%2F19.5.501	Årtun J, Spadafora AT, Shapiro PA. A 3-year follow-up study of various types of orthodontic canine-to-canine retainers. European Journal of Orthodontics 1997;19(5):501-9.	0 Ethics committee not mentioned

Lodi G, Franchini R, Warnakulasuriya S, Varoni EM, Sardella A, Kerr AR, Carras http://doi.org/10.1002/14651858.CD001829.pub4

2016 Interventions for treating oral leukoplakia to prevent oral cancer Primary studies: 14

2015	Nagao 2015 [doi.org/10.1002/ijc.29156](doi.org/10.1002/ijc.29156)	Nagao T, Warnakulasuriya S, Nakamura T, Kato S, Yamamoto K, Fukano H, et al. Treatment of oral leukoplakia with a low-dose of beta-carotene and vitamin C supplements: a randomized controlled trial. International Journal of Cancer 2015;136:1708-17.	1 approved by the scientific and ethical committees of the involved institutes
2014	Mallery 2014 [doi.org/10.1158%2F1078-0432.CCR-13-3159](doi.org/10.1158%2F1078-0432.CCR-13-3159)	Mallery SR, Tong M, Shumway BS, Curran AE, Larsen PE, Ness GM, et al. Topical application of a mucoadhesive freeze-dried black raspberry gel induces clinical and histologic regression and reduces loss of heterozygosity events in premalignant oral intraepithelial lesions: results from a multicentered, placebo-controlled clinical trial. Clinical Cancer Research 2014:20(7):1910-24.	1 approved by the IRBs at all three participating Universities, that is, Ohio State, University of North Carolina, Chapel Hill and Louisville
2013	Armstrong 2013 doi.org/10.1158%2F1940-6207.CAPR-13-0004	Armstrong WB, Taylor TH, Kennedy AR, Melrose RJ, Messadi DV, Gu M, et al. Bowman Birk inhibitor concentrate and oral leukoplakia: a randomized phase IIb trial. Cancer Prevention Research (Philadelphia, Pa.) 2013;6:410-8.	1 approved by the Institutional review boards of the University of California
2009	Sun 2009 doi.org/10.1016%2Fj.oraloncology.2009.06.004	Sun Z, Guan X, Li N, Liu X, Chen X. Chemoprevention of oral cancer in animal models, and effect on leukoplakias in human patients with ZengShengPing, a mixture of medicinal herbs. Oral Oncology 2010;46:105-10.	2 approved by the ethics committee of West China Stomatology Hospital (project number 200705).

Cochrane_Author		Primary studies_	doi
2009	Tsao 2009	Tsao AS, Liu D, Martin J, Tang XM, Lee JJ, El-Naggar AK, et al. Phase II randomized, placebo-controlled trial of green tea extract in patients with high-risk oral premalignant lesions. Cancer Prevention Research (Philadelphia, Pa.) 2009;2:931-41.	doi.org/10.1158%2F1940-6207.CAPR-09-0121
		1 approved by the M.D.Anderson Institutional Review Board	
2008	Papadimitrakop	Papadimitrakopoulou VA, William WN Jr, Dannenberg AJ, Lippman SM, Lee JJ, Ondrey FG, et al. Pilot randomized phase II study of celecoxib in oral premalignant lesions. Clinical Cancer Research 2008;14:2095-101.	doi.org/10.1158%2F1078-0432.CCR-07-4024
		1 approved by the institutional review board of each center	
2004	Mulshine 2004	Mulshine JL, Atkinson JC, Greer RO, Papadimitrakopoulou VA, Van Waes C, Rudy S, et al. Randomized, double-blind, placebo-controlled phase IIb trial of the cyclooxygenase inhibitor ketorolac as an oral rinse in oropharyngeal leukoplakia. Clinical Cancer Research 2004;10(5):1565-73.	doi.org/10.1158%2F1078-0432.CCR-1020-3
		1 approved by the relevant Institutional Review Boards	
2004	Singh 2004	Singh M, Krishanappa R, Bagewadi A, Keluskar V. Efficacy of oral lycopene in the treatment of oral leukoplakia. Oral Oncology 2004;40(6):591-6.	doi.org/10.1016%2Fj.oraloncology.2003.12.011
		1 approved by the participating institute’s review boards for research	
1999	Li 1999	Li N, Sun Z, Han C, Chen J. The chemopreventive effects of tea on human oral precancerous mucosa lesions. Proceedings of the Society for Experimental Biology and Medicine 1999;220(4):218-24.	doi.org/10.3181%2F00379727-220-44369
		--- (article has not been accessed online) ---	
1999	Piattelli 1999	Piattelli A, Fioroni M, Santinelli A, Rubini C. bcl-2 expression and apoptotic bodies in 13-cis-retinoic acid (isotretinoin)-topically treated oral leukoplakia: a pilot study. Oral Oncology 1999;35(3):314-20.	doi.org/10.1016%2FS1368-8375%2898%2900095-5
		0 Ethics committee not mentioned	
1997	Sankaranarayan	Sankaranarayanan R, Mathew B, Varghese C, Sudhakaran PR, Menon V, Jayadeep A, et al. Chemoprevention of oral leukoplakia with vitamin A and beta carotene: an assessment. Oral Oncology 1997;33(4):231-6.	
		1 approved by the Institutional Review Board for Human Studies at the Medical College, Trivandrum, i	
1988	Stich 1988	Stich HF, Hornby AP, Mathew B, Sankaranarayanan R, Nair MK. Response of oral leukoplakias to the administration of vitamin A. Cancer Letters 1988;40(1):93-101.	doi.org/10.1016%2F0304-3835%2888%2990266-2
		0 Ethics committee not mentioned	
1986	Hong 1986	Hong WK, Endicott J, Itri LM, Doos W, Batsakis JG, Bell R, et al. 13-cis-retinoic acid in the treatment of oral leukoplakia. New England Journal of Medicine 1986;315(24):1501-5.	doi.org/10.1056%2FNEJM198612113152401
		--- (article has not been accessed online) ---	

Cochrane_Author	Primary studies_	doi

Ma X, Li C, Jia L, Wang Y, Liu W, Zhou X, Johnson TM, Huang D — http://doi.org/10.1002/14651858.CD005517.pub2

2016 Materials for retrograde filling in root canal therapy — Primary studies: 6

Year	Study	Reference	Ethics
2012	Song 2012 doi.org/10.1016%2Fj.joen.2012.04.008	Song M, Kim E. A prospective randomized controlled study of mineral trioxide aggregate and super ethoxy-benzoic acid as root-end filling materials in endodontic microsurgery. Journal of Endodontics 2012;38(7):875-9	1 approved by the Yonsei University Committee for Research on Human Subjects
2011	Walivaara 2011 doi.org/10.1016%2Fj.tripleo.2011.01.016	Wälivaara DÅ, Abrahamsson P, Fogelin M, Isaksson S. Super-EBA and IRM as root-end fillings in periapical surgery with ultrasonic preparation: a prospective randomized clinical study of 206 consecutive teeth. Oral Surgery, Oral Medicine, Oral Pathology, Oral Radiology, and Endodontics 2011;112(2):258-63	1 approved by the human ethical committee at the University of Lund, Sweden.
2005	Lindeboom 200 doi.org/10.1016%2Fj.tripleo.2005.03.027	Lindeboom JA, Frenken JW, Kroon FH, van der Akker HP. A comparative prospective randomized clinical study of MTA and IRM as root-end filling materials in single-rooted teeth in endodontic surgery. Oral Surgery, Oral Medicine, Oral Pathology, Oral Radiology, and Endodontics 2005;100(4):495-500	0 Ethics committee not mentioned
2003	Chong 2003 doi.org/10.1046/j.1365-2591.2003.00682.x	Chong BS, Pitt Ford TR, Hudson MB. A prospective clinical study of Mineral Trioxide Aggregate and IRM when used as root-end filling materials in endodontic surgery. International Endodontic Journal 2003;36(8):520-6	1 approved by the local ethical committee
2002	Jensen 2002 doi.org/10.1007%2Fs00784-002-0172-5	Jensen SS, Nattestad A, Egdo P, Sewerin I. A prospective, randomized, comparative clinical study of resin composite and glass ionomer cement for retrograde root filling. Clinical Oral Investigations 2002;6(4):236-43	2 approved by the Scientific Ethical Board in Copenhagen, Denmark (J.nr. KF 03-006/96)
1995	Jesslen 1995 doi.org/10.1016%2FS1079-2104%2805%2980082-4	Jesslen P, Zetterqvist L, Heimdahl A. Long-term results of amalgam versus glass ionomer cement as apical sealant after apicectomy. Oral Surgery, Oral Medicine, Oral Pathology, Oral Radiology, and Endodontics 1995;79(1):101-3	1 approved by the local ethical committee of Huddinge Hospital

Macey R, Walsh T, Brocklehurst P, Kerr AR, Liu JL, Lingen MW, Ogden GR, Wa — http://doi.org/10.1002/14651858.CD010276.pub2

2015 Diagnostic tests for oral cancer and potentially malignant disorders in patients presenting with clinically evident lesions — Primary studies: 41

Year	Study	Reference	Ethics
	DIAGNOSTIC TE	REVIEW OF DIAGNOSTIC TESTS	

Manfredi M, Figini L, Gagliani M, Lodi G — http://doi.org/10.1002/14651858.CD005296.pub3

2016 Single versus multiple visits for endodontic treatment of permanent teeth — Primary studies: 25

Cochrane_Author		Primary studies_	doi
2016	Patil 2016	Patil AA, Joshi SB, Bhagwat SV, Patil SA. Incidence of postoperative pain after single visit and two visit root canal therapy: a randomized controlled trial. Journal of Clinical and Diagnostic Research 2016;10(5):ZC09-12	
doi.org/10.7860/JCDR/2016/16465.7724		1 approved by the institutional the ethical committee on human research of K.L.E. University, India.	
2015	Wong 2015	Wong AW, Tsang CS, Zhang S, Li KY, Zhang C, Chu CH. Treatment outcomes of single-visit versus multiple-visit non-surgical endodontic therapy: a randomised clinical trial. BMC Oral Health 2015;15:62	
doi.org/10.1186%2Fs12903-015-0148-x		2 approved by the Institutional Review Board of the University of Hong Kong/Hospital Authority Hong Kong West Cluster (HKU UW 09–303).	
2014	Rao 2014	Rao NK, Kandaswamy R, Umashetty G, Rathore VP, Hotkar C, Patil BS. Post-obturation pain following one-visit and two-visit root canal treatment in necrotic anterior teeth. Journal of International Oral Health 2014;6(2):28-32	
ncbi.nlm.nih.gov/pubmed/24876699		0 Ethics committee not mentioned	
2013	Akbar 2013	Akbar I, Iqbal A, Al-Omiri MK. Flare-up in molars with periapical radiolucency. Journal of Contemporary Dental Practice 2013;14(3):414-8	
doi.org/10.5005%2Fjp-journals-10024-1337		1 approved by the Punjab Dental Hospital	
2013	Doras 2013	Dorasani G, Madhusudhana K, Chinni SK. Clinical and radiographic evaluation of single-visit band multi-visit endodontic treatment of teeth with periapical pathology: an in vivo study. Journal of Conservative Dentistry 2013;16:484-8	
doi.org/10.4103%2F0972-0707.120933		1 approved by the institutional ethical committee for research on human subjects	
2012	Paredes 2012	Paredes-Vieyra J, Enriquez FJ. Success rate of single- versus two-visit root canal treatment of teeth with apical periodontitis: a randomized controlled trial. Journal of Endodontics 2012;38(9):1164-9.	
doi.org/10.1016%2Fj.joen.2012.05.021		1 approved by the subjects review committee	
2012	Singh 2012	Singh S, Garg A. Incidence of post-operative pain after single visit and multiple visit root canal treatment: a randomized controlled trial. Journal of Conservative Dentistry 2012;15(4):323-7	
doi.org/10.4103%2F0972-0707.101888		1 approved by the committee on human research	
2010	Wang 2010	Wang C, Xu P, Ren L, Dong G, Ye L. Comparison of post-obturation pain experience following one-visit and two visit root-canal treatment on teeth with vital pulps: a randomized controlled trial. International Endodontic Journal 2010;43:692-7	
doi.org/10.1111/j.1365-2591.2010.01748.x		1 approved by the West China School of Stomatology of SichuanUniversity committee on human research	
2010	Xiao 2010	Xiao D, Zhang DH. A clinical study of one-visit endodontic treatment for infected root canals. Hua Xi Kou Qiang Yi Xue Za Zhi 2010;28:57-60	
ncbi.nlm.nih.gov/pubmed/15611847		--- (article has not been accessed online) ---	

Cochrane_Author		Primary studies_	doi
2009	Ince 2009	Ince B, Ercan E, Dalli M, Dulgergil CT, Zorba YO, Colak H. Incidence of postoperative pain after single- and multi-visit endodontic treatment in teeth with vital and non-vital pulp. European Journal of Dentistry 2009;3:273-9	ncbi.nlm.nih.gov/pubmed/19826598
		0 Ethics committee not mentioned	
2008	Penenis 2008	Penenis VA, Fitzgerald PI, Fayad MI, Wenckus CS, BeGole EA, Johnson BR. Outcome of one-visit and two-visit endodontic treatment of necrotic teeth with apical periodontitis: a randomized controlled trial with one-year evaluation. Journal of Endodontics 2008;34(3):251-7	doi.org/10.1016%2Fj.joen.2007.12.015
		1 approved by the University of Illinois at Chicago Institutional Review Board	
2008	Risso 2008	Risso PA, Cunha AJLA, Araujo MCP, Luiz RR. Postobturation pain and associated factors in adolescent patients undergoing one- and two-visit root canal treatment. Journal of Dentistry 2008;36:928-34	doi.org/10.1016%2Fj.jdent.2008.07.006
		1 approved by the local Research Ethics Committee	
2007	Molander 2007	Molander A, Warfvinge J, Reit C, Kvist T. Clinical and radiographic evaluation of one-and two-visit endodontic treatment of asymptomatic necrotic teeth with apical periodontitis: a randomized clinical trial. Journal of Endodontics 2007;33(10):1145-48.	doi.org/10.1016%2Fj.joen.2007.07.005
		1 approved by the Göteborg University committee for research on human subjects	
2006	Al-negr 2006	Al-Negrish AR, Habahbeh R. Flare up rate related to root canal treatment of asymptomatic pulpally necrotic central incisor teeth in patients attending a military hospital. Journal of Dentistry 2006;34(9):635-40.	doi.org/10.1016%2Fj.jdent.2005.12.007
		0 Ethics committee not mentioned	
2006	Gesi 2006	Gesi A, Hakeberg M, Warfvinge J, Bergenholtz G. Incidence of periapical lesions and clinical symptoms after pulpectomy-a clinical and radiographic evaluation of 1- versus 2-session treatment. Oral Surgery, Oral Medicine, Oral Pathology, Oral Radiology, and Endodontics 2006;101(3):379-88	doi.org/10.1016%2Fj.tripleo.2005.05.073
		0 Ethics committee not mentioned	
2006	Ghodd 2006	Ghoddusi J, Javidi M, Zarrabi MH, Bagheri H. Flare-ups incidence and severity after using calcium hydroxide as intracanal dressing. The New York State Dental Journal 2006;72(4):24-8	ncbi.nlm.nih.gov/pubmed/16925009
		--- (article has not been accessed online) ---	
2004	Oginni 2004	Oginni AO, Udoye CI. Endodontic flare-ups: comparison of incidence between single and multiple visit procedures in patients attending a Nigerian teaching hospital. BMC Oral Health 2004;4(1):4	doi.org/10.1186%2F1472-6831-4-4
		0 Ethics committee not mentioned	
2004	Yoldas 2004	Yoldas O, Topuz A, Isci AS, Oztunc H. Postoperative pain after endodontic retreatment: single - versus two -visit treatment. Oral Surgery, Oral Medicine, Oral Pathology, Oral Radiology, and Endodontics 2004;98(4):483-7	doi.org/10.1016%2Fj.tripleo.2004.03.009
		1 approved by the University of Cxukurova Faculty of Dentistry Institutional Review Board	

2002 Direnz 2002
doi.org/10.1067%2Fmoe.2002.121900
DiRenzo A, Gresla T, Johnson BR, Rogers M, Tucker D, BeGole EA. Postoperative pain after 1- and 2-visit root canal therapy. Oral Surgery, Oral Medicine, Oral Pathology, Oral Radiology, and Endodontics 2002;93(5):605-10
1 approved by the University of Illinois at Chicago Institutional Review Board

2002 Peters 2002
doi.org/10.1046/j.1365-2591.2002.00541.x
Peters LB, Wesselink PR. Periapical healing of endodontically treated teeth in one and two visits obturated in the presence or absence of detectable microorganisms. International Endodontic Journal 2002;35(8):660-7.
0 Ethics committee not mentioned

2000 Weiger 2000
doi.org/10.1046/j.1365-2591.1999.00298.x
Weiger R, Rosendahl R, Lost C. Influence of calcium hydroxide intracanal dressings on the prognosis of teeth with endodontically induced periapical lesions. International Endodontic Journal 2000;33(3):219-26
0 Ethics committee not mentioned

1999 Trope 1999
doi.org/10.1016%2FS0099-2399%2806%2981169-6
Trope M, Delano EO, Orstavik D. Endodontic treatment of teeth with apical periodontitis: single vs. multivisit treatment. Journal of Endodontics 1999;25(5):345-50.
1 approved by the Committee on Investigations Involving Human Subjects at the University of North Carolina, School of Dentistry

1998 Albashaireh 199
doi.org/10.1016%2FS0300-5712%2897%2900006-7
Albashaireh ZS, Alnegrish AS. Postobturation pain after single- and multiple-visit endodontic therapy. A prospective study. Journal of Dentistry 1998;26(3):227-32.
1 approved by the Ethics Committee of the University.

1982 Mulhern 1982
doi.org/10.1016%2FS0099-2399%2882%2980197-0
Mulhern JM, Patterson SS, Newton CW, Ringel AM. Incidence of postoperative pain after one-appointment endodontic treatment of asymptomatic pulpal necrosis in single-rooted teeth. Journal of Endodontics 1982;8(8):370-5
0 Ethics committee not mentioned

1978 Soltanoff 1978
doi.org/10.1016%2FS0099-2399%2878%2980144-7
Soltanoff W. A comparative study of the single-visit and the multiple-visit endodontic procedure. Journal of Endodontics 1978;4(9):278-81
0 Ethics committee not mentioned

Marinho VC, Chong LY, Worthington HV, Walsh T http://doi.org/10.1002/14651858.CD002284.pub2

2016 Fluoride mouthrinses for preventing dental caries in children and adolescents Primary studies: 37

2005 Moberg Sköld 2
doi.org/10.1159%2F000088191
Moberg Sköld U, Birkhed D, Borg E, Petersson LG. Approximal caries development in adolescents with low to moderate caries risk after different 3-year school-based supervised fluoride mouth rinsing programmes. Caries Research 2005 Nov-Dec;39(6):529-35
1 approved by the ethics committee at Göteborg University

Year	Cochrane_Author	Primary studies_
1998	Petersson 1998 ncbi.nlm.nih.gov/pubmed/9879918	Petersson LG, Svanholm I, Andersson H, Magnusson K. Approximal caries development following intensive fluoride mouthrinsing in teenagers. A 3-year radiographic study. European Journal of Oral Sciences 1998;106:1048-51<br>--- (article has not been accessed online) ---
1992	Heidmann 1992 [doi.org/10.1111/j.1600-0528.1992.tb01543.x](doi.org/10.1111/j.1600-0528.1992.tb01543.x)	Heidmann J, Poulsen S, Arnbjerg D, Kirkegaard E, Laurberg L. Caries development after termination of a fluoride rinsing program. Community Dentistry and Oral Epidemiology 1992;20:118-21<br>--- (article has not been accessed online) ---
1991	Spets-Happone [doi.org/10.1111/j.1600-0722.1991.tb01875.x](doi.org/10.1111/j.1600-0722.1991.tb01875.x)	Spets-Happonen S, Luoma H, Forss H, Kentala J, Alaluusua S. Effects of a chlorhexidine-fluoride-strontium rinsing program on caries, gingivitis and some salivary bacteria among Finnish schoolchildren. Scandinavian Journal of Dental Research 1991;99:130-8<br>1 approved by the local ethical committee for medical research
1989	Bastos 1989	Bastos JRM, Lopes ES. Fluoride mouthwashes: anticaries effectiveness of a weekly mouthrinsing program using sodium fluoride or sodium monofluorophosphate after 32 months in scholars from 9 to 12 years old [Bochechos com fluoretos: efeito anticariogenico de bochechos semanais com solucoes de fluoreto de sodio ou monofluor fosfato de sodio, apos 32 meses em escolares de 9-12 anos de idade]. Revista da Associacao Paulista de Cirurgioes Dentistas 1989;43:34-6<br>--- (article has not been accessed online) ---
1989	Deliefde 1989 ncbi.nlm.nih.gov/pubmed/2797545	De Liefde B. Identification and preventive care of high caries-risk children: a longitudinal study. New Zealand Dental Journal 1989;85:112-6.<br>--- (article has not been accessed online) ---
1987	Molina 1987 ncbi.nlm.nih.gov/pubmed/2641954	Molina MX, Rodriguez FG, Urbina T, Vargas S. Effect of weekly mouthrinses with 0.2% neutral NaF solution on caries incidence in first permanent molars [Efecto de enjuagatorios semanales con una solucion neutra de NaF al 0.2% en la incidencia de caries en primeros molares definitivos]. Odontologia Chilena 1989;37:176-82<br>--- (article has not been accessed online) ---
1987	Ruuiken 1987 [doi.org/10.1111/j.1600-0528.1987.tb00481.x](doi.org/10.1111/j.1600-0528.1987.tb00481.x)	Ruiken R, Truin GJ, Konig K, Vogels A, van 't Hof M. Clinical cariostatic effectiveness of a NaF rinse in a low prevalence child population. Community Dentistry and Oral Epidemiology 1987;15:57-9<br>--- (article has not been accessed online) ---
1986	Vanwyk 1986 ncbi.nlm.nih.gov/pubmed/3462964	van Wyk I, van Wyk CW. The effectiveness of a 0.2 percent and a 0.05 percent neutral NaF mouthrinsing programme. Journal of the Dental Association of South Africa 1986;41:35-40.<br>--- (article has not been accessed online) ---
1984	Poulsen 1984 [doi.org/10.1111/j.1600-0528.1984.tb01456.x](doi.org/10.1111/j.1600-0528.1984.tb01456.x)	Poulsen S, Kirkegaard E, Bangsbo G, Bro K. Caries clinical trial of fluoride rinses in a Danish Public Child Dental Service. Community Dentistry and Oral Epidemiology 1984;12:283-7<br>--- (article has not been accessed online) ---

Cochrane_Author		Primary studies_	doi
1983	Blinkhorn 1983	Blinkhorn AS, Holloway PJ, Davies TG. Combined effects of a fluoride dentifrice and mouthrinse on the incidence of dental caries. Community Dentistry and Oral Epidemiology 1983;11:7-11	doi.org/10.1111/j.1600-0528.1983.tb01347.x
		--- (article has not been accessed online) ---	
1982	Driscoll 1982	Driscoll WS, Swango PA, Horowitz AM, Kingman A. Caries-preventive effects of daily and weekly fluoride mouthrinsing in a fluoridated community: final results after 30 months. Journal of the American Dental Association 1982;105:1010-3	doi.org/10.14219%2Fjada.archive.1982.0401
		--- (article has not been accessed online) ---	
1982	Heifetz 1982	Heifetz SB, Meyers RJ, Kingman A. A comparison of the anticaries effectiveness of daily and weekly rinsing with sodium fluoride solutions: final results after three years. Pediatric Dentistry 1982;4:300-3	aapd.org/assets/1/25/Heifetz-04-04.pdf
		0 Ethics committee not mentioned	
1982	Ringelberg 198	Ringelberg ML, Conti AJ, Ward CB, Clark B, Lotzkar S. Effectiveness of different concentrations and frequencies of sodium fluoride mouthrinse. Pediatric Dentistry 1982;4:305-8	
		--- (article has not been accessed online) ---	
1981	Craig 1981	Craig EW, Suckling GW, Pearce EI. The effect of a preventive programme on dental plaque and caries in school children. New Zealand Dental Journal 1981;77:89-93	doi.org/10.1111/j.1600-0528.1980.tb01317.x
		--- (article has not been accessed online) ---	
1981	Duany 1981	Wallenstein S, Fleiss JL, Chilton NW. Confidence intervals for percentage reduction in caries increments [Describes Duany L, Zinner DD, and Chilton, NW: unpublished study]. Journal of Dental Research 1982;61:828-30	doi.org/10.1177%2F00220345820610063801
		--- (article has not been accessed online) ---	
1981	Moreira 1981	Moreira BH, Guimaraes LO, Vieira S, Piedade EF. Fluoride mouthwashes in combination with fluoridation of the public water supply for the prevention of dental caries [Bochecho com fluor associado a fluoretacao da agua de abastecimento publico, na prevencao da carie dentaria]. Revista da Associacao Paulista de Cirurgioes Dentistas 1981:35:296-301	ncbi.nlm.nih.gov/pubmed/6947332
		--- (article has not been accessed online) ---	
1980	DePaola 1980	DePaola PF, Soparkar M, Van Leeuwen M, DeVelis R. The anticaries effect of single and combined topical fluoride systems in school children. Archives of Oral Biology 1980;25:649-53	doi.org/10.1016%2F0003-9969%2880%2990095-3
		0 Ethics committee not mentioned	
1979	Ringelberg 197	Ringelberg ML, Webster DB, Dixon DO, LeZotte DC. The caries-preventive effect of amine fluorides and inorganic fluorides in a mouthrinse or dentifrice after 30 months of use. Journal of the American Dental Association 1979;98:202-8	doi.org/10.14219%2Fjada.archive.1979.0466
		--- (article has not been accessed online) ---	

Cochrane_Author		Primary studies_	doi
1977	Ashley 1977	Ashley FP, Mainwaring PJ, Emslie RD, Naylor MN. Clinical testing of a mouthrinse and a dentifrice containing fluoride. A two-year supervised study in school children. British Dental Journal 1977;143:333-8	doi.org/10.1038%2Fsj.bdj.4804004
		--- (article has not been accessed online) ---	
1977	DePaola 1977	DePaola PF, Soparkar P, Foley S, Bookstein F, Bakhos Y. Effect of high-concentration ammonium and sodium fluoride rinses on dental caries in schoolchildren. Community Dentistry and Oral Epidemiology 1977;5:7-14	doi.org/10.1111/j.1600-0528.1977.tb01611.x
		--- (article has not been accessed online) ---	
1977	Mcconchie 197	McConchie JM, Richardson AS, Hole LW, McCombie F, Kolthammer J. Caries-preventive effect of two concentrations of stannous fluoride mouthrinse. Community Dentistry and Oral Epidemiology 1977;5:278-83	doi.org/10.1111/j.1600-0528.1977.tb01014.x
		--- (article has not been accessed online) ---	
1975	Finn 1975	Finn SB, Moller P, Jamison H, Regattieri L, Manson Hing L. The clinical cariostatic effectiveness of two concentrations of acidulated phosphate-fluoride mouthwash. Journal of the American Dental Association 1975;90:398-402	doi.org/10.14219%2Fjada.archive.1975.0063
		--- (article has not been accessed online) ---	
1975	Laswell 1975	Laswell HR, Packer MW, Wiggs JS. Cariostatic effects of fluoride mouthrinses in a fluoridated community. Journal of the Kentucky Dental Association 1975;27:21-5.	ncbi.nlm.nih.gov/pubmed/1075097
		--- (article has not been accessed online) ---	
1975	Packer 1975	Packer MW, Laswell HR, Doyle J, Naff HH, Brown F. Cariostatic effects of fluoride mouthrinses in a non-fluoridated community. Journal of the Tennessee Dental Association 1975;55:22-6	ncbi.nlm.nih.gov/pubmed/1069886
		--- (article has not been accessed online) ---	
1974	Gallagher 1974	Gallagher SJ, Glassgow I, Caldwell R. Self-application of fluoride by rinsing. Journal of Public Health Dentistry 1974;34:13-21.	doi.org/10.1111/j.1752-7325.1974.tb00669.x
		--- (article has not been accessed online) ---	
1973	Heifetz 1973	Heifetz SB, Driscoll WS, Creighton WE. The effect on dental caries of weekly rinsing with a neutral sodium fluoride or an acidulated phosphate-fluoride mouthwash. Journal of the American Dental Association 1973;87:364-8.	doi.org/10.14219%2Fjada.archive.1973.0387
		--- (article has not been accessed online) ---	
1973	Radike 1973	Radike AW, Gish CW, Peterson JK, King JD, Segreto VA. Clinical evaluation of stannous fluoride as an anticaries mouthrinse. Journal of the American Dental Association 1973;86:404-8.	doi.org/10.14219%2Fjada.archive.1973.0061
		--- (article has not been accessed online) ---	

Year	Author	Primary study	doi	Note
1973	Rug-gunn 1973	Rugg-Gunn AJ, Holloway PJ, Davies TG. Caries prevention by daily fluoride mouthrinsing. Report of a three- year clinical trial. British Dental Journal 1973;135:353-60	doi.org/10.1038%2Fsj.bdj.4803087	--- (article has not been accessed online) ---
1972	Brandt 1972	Brandt RS, Slack GL, Waller DF. The use of a sodium fluoride mouthwash in reducing the dental caries increment in eleven year old English school children. Proceedings of the British Paedodontic Society 1972;2:23-5	ncbi.nlm.nih.gov/pubmed/4405623	--- (article has not been accessed online) ---
1972	Moreira 1972	Moreira BH, Tumang AJ. Prevention of dental caries by means of mouthwashes with 0.1 solutions of sodium fluoride. Results of a 2 year study [Prevencao da carie dentaria atraves de bochechos com solucoes de fluoreto de sodio a 0.1%. Resultados apos dois anos de estudos]. Revista Brasileira de Odontologia 1972;29:37-42	ncbi.nlm.nih.gov/pubmed/4502245	--- (article has not been accessed online) ---
1971	Horowitz 1971	Horowitz HS, Creighton WE, McClendon BJ. The effect on human dental caries of weekly oral rinsing with a sodium fluoride mouthwash: a final report. Archives of Oral Biology 1971;16:609-16	doi.org/10.1016%2F0003-9969%2871%2990064-1	0 Ethics committee not mentioned
1971	Horowitz 1971a	Horowitz HS, Creighton WE, McClendon BJ. The effect on human dental caries of weekly oral rinsing with a sodium fluoride mouthwash: a final report. Archives of Oral Biology 1971;16:609-16	doi.org/10.1016%2F0003-9969%2871%2990064-1	--- (article has not been accessed online) ---
1967	koch 1967	Koch G, Lindhe J. The effect of supervised oral hygiene on the gingiva of children. The effect of sodium fluoride. Journal of Periodontal Research 1967;2:64-9	doi.org/10.1111/j.1600-0765.1967.tb01997.x	0 Ethics committee not mentioned
1967	Koch 1967a	Koch G. Effect of sodium fluoride in dentifrice and mouthwash on incidence of dental caries in school children. 12. Effect of surpervised rinsing of the mouth with 0.5 per cent sodium fluoride solution at the regular visits to the school dentist. A 3-year double-blind clinical test. Odontologisk Revy 1967;18:89-96		--- (article has not been accessed online) ---
1967	Kock 1967b	Koch G. Effect of sodium fluoride in dentifrice and mouthwash on incidence of dental caries in school children. 13. Effect of supervised rinsing of the mouth with 0.05 per cent sodium fluoride solution at the regular visits to the school dentist. A 2-year double-blind test. Odontologisk Revy 1967;18:97-100		--- (article has not been accessed online) ---
1965	Torell 1965	Torell P, Ericsson Y. Two year clinical tests with different methods of local caries-preventive fluorine application in Swedish school-children (Part I: The Goteborg study). Acta Odontologica Scandinavica 1965;23:287-312	doi.org/10.3109%2F00016356509007517	--- (article has not been accessed online) ---

Cochrane_Author	Primary studies_	doi
Marinho VC, Worthington HV, Walsh T, Chong LY		http://doi.org/10.1002/14651858.CD002280.pub2

2015 Fluoride gels for preventing dental caries in children and adolescents — Primary studies: 28

Year	Study	Reference	Ethics
2005	Jiang 2005<br>doi.org/10.1016%2Fj.jdent.2004.10.023	Jiang H, Tai B, Du M, Peng B. Effect of professional application of APF foam on caries reduction in permanent first molars in 6-7-year-old children: 24-month clinical trial. Journal of Dentistry 2005;33(6):469-73.	1 approved by the Ethics Committee of the School of Dentistry, University of Wuhan
2005	Truin 2005<br>doi.org/10.1177%2F154405910508400504	Truin GJ, van't Hof MA. Professionally applied fluoride gel in low-caries 10.5-year olds. Journal of Dental Research 2005;84(5):418-21.	2 approved by the research ethics committee of Radboud University (CEOM Nr. 9406-0682)
2004	Van Rijkom 200<br>doi.org/10.1159%2F000075935	van Rijkom HM, Truin GJ, van't Hof MA. Caries-inhibiting effect of professional fluoride gel application in low-caries children initially aged 4.5-6.5 years. Caries Research 2004;38(2):115-23.	2 approved by the research ethics committee of the University of Nijmegen (CEOM No. 9406-0682)
1999	Gisselsson 1999<br>doi.org/10.1080%2F000163599429020	Gisselsson H, Birkhed D, Emilson CG. Effect of professional flossing with NaF or SnF2 gel on approximal caries in 13-16-year-old schoolchildren. Acta Odontologica Scandinavica 1999;57(2):121-5.	1 approved by the Ethics Committee at Lund University and the Swedish National Board of Health and Welfare, Drug Division, Uppsala, Sweden.
1992	Olivier 1992<br>doi.org/10.1111/j.1600-0528.1992.tb00671.x	Olivier M, Brodeur JM, Simard PL. Efficacy of APF treatments without prior toothcleaning targeted to high-risk children. Community Dentistry and Oral Epidemiology 1992;20(1):38-42.	--- (article has not been accessed online) ---
1991	Ran 1991<br>doi.org/10.1111/j.1365-2842.1991.tb00062.x	Ran F, Gedalia I, Fried M, Hadani P, Tved A. Effectiveness of fortnightly tooth brushing with amine fluorides in caries-prone subjects. Journal of Oral Rehabilitation 1991;18(4):311-6.	0 Ethics committee not mentioned
1988	Treide 1988<br>ncbi.nlm.nih.gov/pubmed/3269091	Treide A, Treide B. The anticaries effectiveness of newly developed fluoride-containing gels following 3 years of clinical use in preschool children. Stomatologie der DDR 1988;38(10):708-12.	--- (article has not been accessed online) ---
1985	Hagan 1985<br>aapd.org/assets/1/25/Hagan-07-03.pdf	Hagan PP, Rozier RG, Bawden JW. The caries-preventive effects of full-strength and half-strength topical acidulated phosphate fluoride. Pediatric Dentistry 1985;7(3):185-91.	0 Ethics committee not mentioned

Cochrane_Author		Primary studies_	doi
1983	Mestrinho 1983	Mestrinho HD, Bijella MFTB, Bijella VT, Lopes ES. Prevention of dental caries through topical application of APF gel with plastic trays [Prevenção da cárie dental pela aplicação tópica de gel de flúor fosfato acidulado, através de moldeiras plásticas]. Odontologo Moderno 1983;10(1-2):29-32.	
		--- (article has not been accessed online) ---	
1981	Bijella 1981	Bijella MF, Bijella VT, Lopes ES, Bastos JR. Comparison of dental prophylaxis and toothbrushing prior to topical fluoride applications. Community Dentistry and Oral Epidemiology 1985;13(4):208-11.	doi.org/10.1111/j.1600-0528.1985.tb01904.x
		--- (article has not been accessed online) ---	
1980	Cobb 1980	Cobb BH, Rozier GR, Bawden JW. A clinical study of the caries preventive effects of an APF solution and APF thixotropic gel. Pediatric Dentistry 1980;2(4):263-6.	aapd.org/assets/1/25/Cobb-02-04.pdf
		0 Ethics committee not mentioned	
1980	DePaola 1980	DePaola PF, Soparkar M, Van Leeuwen M, DeVelis R. The anticaries effect of single and combined topical fluoride systems in school children. Archives of Oral Biology 1980;25(10):649-53.	doi.org/10.1016%2F0003-9969%2880%2990095-3
		0 Ethics committee not mentioned	
1978	Abadia 1978	Abadia SMS. Prevenção da cárie dentária através da aplicação tópica de gel de flúor fosfato ácido, utilizando-se isolamento relativo e absoluto [dissertation]. Baurú (SP): Universidade de São Paulo, 1978.	
		--- (article has not been accessed online) ---	
1978	Englander 1978	Englander HR, Mellberg JR, Engler WO. Observations on dental caries in primary teeth after frequent fluoride toplications in a program involving other preventives. Journal of Dental Research 1978;57(9-10):855-60.	doi.org/10.1177%2F00220345780570090101
		--- (article has not been accessed online) ---	
1978	Mainwaring 19	Mainwaring PJ, Naylor MN. A three-year clinical study to determine the separate and combined caries-inhibiting effects of sodium monofluorophosphate toothpaste and an acidulated phosphate-fluoride gel. Caries Research 1978;12(4):202-12.	doi.org/10.1159%2F000260334
		--- (article has not been accessed online) ---	
1976	Shern 1976	Shern RJ, Duany LF, Senning RS, Zinner DD. Clinical study of an amine fluoride gel and acidulated phosphate fluoride gel. Community Dentistry and Oral Epidemiology 1976;4(4):133-6.	doi.org/10.1111/j.1600-0528.1976.tb00968.x-i1
		--- (article has not been accessed online) ---	
1974	Horowitz 1974	Horowitz HS, Heifetz SB, McClendon BJ, Viegas AR, Guimaraes LO, Lopes ES. Evaluation of self-administered prophylaxis and supervised toothbrushing with acidulated phosphate fluoride. Caries Research 1974;8(1):39-51.	doi.org/10.1159%2F000260092
		--- (article has not been accessed online) ---	

Cochrane_Author		Primary studies_ / doi
1973	Trubman 1973	Trubman A, Crellin JA. Effect on dental caries of self-application of acidulated phosphate fluoride paste and gel. Journal of the American Dental Association 1973;86(1):153-7.
doi.org/10.1016%2FS0002-8177%2873%2961038-0		--- (article has not been accessed online) ---
1972	Szwejda 1972	Szwejda LF. Fluorides in community programs; a study of four years of various fluorides applied topically to the teeth of children in fluoridated communities. Journal of Public Health Dentistry 1972;32(1):25-33.
doi.org/10.1111/j.1752-7325.1972.tb03938.x		--- (article has not been accessed online) ---
1971	Englander 1971	Englander HR, Sherrill LT, Miller BG, Carlos JP, Mellberg JR, Senning RS. Incremental rates of dental caries after repeated topical sodium fluoride applications in children with lifelong consumption of fluoridated water. Journal of the American Dental Association 1971;82(2):354-8.
doi.org/10.14219%2Fjada.archive.1971.0042		--- (article has not been accessed online) ---
1971	Horowitz 1971	Horowitz HS, Doyle J. The effect on dental caries of topically applied acidulated phosphate-fluoride: results after three years. Journal of the American Dental Association 1971;82(2):359-65.
doi.org/10.14219%2Fjada.archive.1971.0063		--- (article has not been accessed online) ---
1970	Bryan 1970	Bryan ET, Williams JE. The cariostatic effectiveness of a phosphate-fluoride gel administered annually to school children; final results. Journal of Public Health Dentistry 1970;30(1):13-6.
doi.org/10.1111/j.1752-7325.1970.tb00531.x		--- (article has not been accessed online) ---
1970	Cons 1970	Cons NC, Janerich DT, Senning RS. Albany topical fluoride study. Journal of the American Dental Association 1970;80(4):777-81.
doi.org/10.14219%2Fjada.archive.1970.0113		--- (article has not been accessed online) ---
1970	Heifetz 1970	Heifetz SB, Horowitz HS, Driscoll WS. Evaluation of a self-administered procedure for the topical application of acidulated phosphate-fluoride. Journal of Dental Research 1968;102:Abstr No 257 (Abstract).
		--- (article has not been accessed online) ---
1970	Ingraham 1970	Ingraham RQ, Williams JE. An evaluation of the utility of application and cariostatic effectiveness of phosphate-fluorides in solution and gel states. Journal of Tennessee State Dental Association 1970;50(1):5-12.
ncbi.nlm.nih.gov/pubmed/4391820		--- (article has not been accessed online) ---
1970	Marthaler 1970	Marthaler TM, Konig KG, Muhlemann HR. The effect of a fluoride gel used for supervised toothbrushing 15 or 30 times per year. Helvetica Odontologica Acta 1970;14(2):67-77.
ncbi.nlm.nih.gov/pubmed/4395021		--- (article has not been accessed online) ---

1970	Marthaler 1970 ncbi.nlm.nih.gov/pubmed/4395021	Marthaler TM, Konig KG, Muhlemann HR. The effect of a fluoride gel used for supervised toothbrushing 15 or 30 times per year. Helvetica Odontologica Acta 1970;14(2):67-77.	--- (article has not been accessed online) ---
1967	Englander 1967 doi.org/10.14219%2Fjada.archive.1967.0266	Englander HR, Keyes PH, Gestwicki M, Sultz HA. Clinical anticaries effect of repeated topical sodium fluoride applications by mouthpieces. Journal of the American Dental Association 1967;75(3):638-44.	--- (article has not been accessed online) ---

Marinho VC, Worthington HV, Walsh T, Clarkson JE http://doi.org/10.1002/14651858.CD002279.pub2

2013 Fluoride varnishes for preventing dental caries in children and adolescents — Primary studies: 22

2012	Arruda 2012 [doi.org/10.1111/j.1600-0528.2011.00656.x](doi.org/10.1111/j.1600-0528.2011.00656.x)	Arruda AO, Senthamarai Kannan R, Inglehart MR, Rezende CT, Sohn W. Effect of 5% fluoride varnish application on caries among school children in rural Brazil: a randomized controlled trial. Community Dentistry and Oral Epidemiology 2012;40(3):267-76.	1 approved by the Institutional Review Board for the HealthSciences at the University of Michigan, Ann Arbor campus, MI
2012	Liu 2012 [doi.org/10.1177%2F0022034512452278](doi.org/10.1177%2F0022034512452278)	Liu BY, Lo EC, Chu CH, Lin HC. Randomized trial on fluorides and sealants for fissure caries prevention. Journal of Dental Research 2012;91(8):753-8.	1 approved by the University of Hong Kong
2011	Gugwad 2011 doi.org/10.5005%2Fjp-journals-10024-1068	Gugwad SC, Shah P, Lodaya R, Bhat C, Tandon P, Choudhari S, et al. Caries prevention effect of intensive application of sodium fluoride varnish in molars in children between age 6 and 7 years. Journal of Contemporary Dental Practice 2011;12:408-13.	1 approved by the institutional review board.
2011	Milsom 2011 [doi.org/10.1177%2F0022034511422063](doi.org/10.1177%2F0022034511422063)	Milsom KM, Blinkhorn AS, Walsh T, Worthington HV, Kearney-Mitchell P, Whitehead H, et al. A cluster-randomized controlled trial: fluoride varnish in school children. Journal of Dental Research 2011;90:1306-11.	1 approved by the Preston Research Ethics Committee
2011	Tagliaferro 201 ncbi.nlm.nih.gov/pubmed/21698991	Tagliaferro EP, Pardi V, Ambrosano GM, Meneghim Mde C, da Silva SR, Pereira AC. Occlusal caries prevention in high and low risk schoolchildren. A clinical trial. American Journal of Dentistry 2011;24:109-14.	2 approved by the research ethics committee of the Piracicaba Dental School, UNICAMP, protocl # 025/2004
2008	Lawrence 2008 [doi.org/10.1111/j.1600-0528.2008.00427.x](doi.org/10.1111/j.1600-0528.2008.00427.x)	Lawrence HP, Binguis D, Douglas J, McKeown L, Switzer B, Figueiredo R, et al. A 2-year community-randomized controlled trial of fluoride varnish to prevent early childhood caries in Aboriginal children. Community Dentistry and Oral Epidemiology 2008;36:503-16.	1 approved by the Univers ityof Toronto Health Sciences I Research Ethics Board(REB) and Health Canada’s REB

Cochrane_Author		Primary studies_	doi
2008	Salazar 2008	Salazar M. [Efetividade da aplicação semestral de verniz fluoretado no controle da cárie dentária em pré-escolares: resultados após 12 meses de acompanhamento]. Effectiveness of Bi-Annual Fluoride Varnish Application in the Control of Dental Caries in Preschool Children: Results after 12 Months of Follow-Up [Thesis]. Rio de Janeiro, Brazil: Universidade do Estado do Rio de Janeiro. 2008.	--- (article has not been accessed online) ---
2008	Yang 2008<br>ncbi.nlm.nih.gov/pubmed/18605454	Yang G, Lin JH, Wang JH, Jiang L. Evaluation of the clinical effect of fluoride varnish in preventing caries of primary teeth. West China Journal of Stomatology 2008;26(2):159-61.	--- (article has not been accessed online) ---
2007	Hardman 2007<br>doi.org/10.1159%2F000104795	Hardman MC, Davies GM, Duxbury JT, Davies RM. A cluster randomised controlled trial to evaluate the effectiveness of fluoride varnish as a public health measure to reduce caries in children. Caries Research 2007;41:371-6.	1 approved by the Local Education Authority
2006	Borutta 2006<br>doi.org/10.1055%2Fs-2006-927247	Borutta A, Reuscher G, Hufnagl S, Möbius S. Caries prevention with fluoride varnishes among preschool children [Kariesprophylaxe mit Fluoridlacken bei Vorschulkindern]. Gesundheitswesen 2006;68(11):731-4.	--- (article has not been accessed online) ---
2006	Weintraub 200<br>doi.org/10.1177%2F154405910608500211	Weintraub JA, Ramos-Gomez F, Jue B, Shain S, Hoover CI, Featherstone JD, et al. Fluoride varnish efficacy in preventing early childhood caries. Journal of Dental Research 2006;85(2):172-6.	1 approved by the University of California, San Francisco Institutional Review Board
2005	Sköld 2005<br>doi.org/10.1159%2F000084833	Sköld U, Petersson LG, Lith A, Birkhed D. Effect of school-based fluoride varnish programmes on approximal caries in adolescents from different caries risk areas. Caries Research 2005;39(4):273-9.	1 approved by the ethics committees of the Universities of Göteborg and Lund
2002	Chu 2002<br>doi.org/10.1177%2F154405910208101109	Chu CH, Lo EC, Lin HC. Effectiveness of silver diamine fluoride and sodium fluoride varnish in arresting dentin caries in Chinese pre-school children. Journal of Dental Research 2002;81:767-70.	1 approved by the Ethics Committee of the Faculty of Dentistry, University of Hong Kong
1997	Bravo 1997<br>doi.org/10.1177%2F154405910508401209	Bravo M, Montero J, Bravo JJ, Baca P, Llodra JC. Sealant and fluoride varnish in caries: a randomized trial. Journal of Dental Research 2005;84(12):1138-43.	1 approved by the University of Granada Faculty of Dentistry Ethics Committee,
1991	Borutta 1991<br>ncbi.nlm.nih.gov/pubmed/1756217	Borutta A, Kunzel W, Rubsam F. The caries-protective efficacy of 2 fluoride varnishes in a 2-year controlled clinical trial [Kariesprotektive Wirksamkeit zweier Fluoridlacke in einer klinisch kontrollierten Zweijahresstudie]. Deutsche Zahn Mund und Kieferheilkunde Zentralblatt 1991;79:543-9.	--- (article has not been accessed online) ---

1991 Frostell 1991
doi.org/10.1159%2F000261381
Frostell G, Birkhed D, Edwardsson S, Goldberg P, Petersson LG, Priwe C, et al. Effect of partial substitution of invert sugar for sucrose in combination with Duraphat treatment on caries development in preschool children: the Malmö Study. Caries Research 1991;25(4):304-10.
--- (article has not been accessed online) ---

1990 Tewari 1990
Tewari A, Chawla HS, Utreja A. Comparative evaluation of the role of NaF, APF & Duraphat topical fluoride applications in the prevention of dental caries--a 2 1/2 years study. Journal of the Indian Society of Pedodontics and Preventative Dentistry 1990;8:28-35.
--- (article has not been accessed online) ---

1985 Clark 1985
doi.org/10.1111/j.1600-0528.1985.tb01676.x
Clark DC, Stamm JW, Quee TC, Robert G. Results of the Sherbrooke-Lac Megantic fluoride varnish study after 20 months. Community Dentistry and Oral Epidemiology 1985;13:61-4.
--- (article has not been accessed online) ---

1984 Holm 1984
doi.org/10.3109%2F00016358408993871
Holm GB, Holst K, Mejare I. The caries-preventive effect of a fluoride varnish in the fissures of the first permanent molar. Acta Odontologica Scandinavica 1984;42:193-7.
--- (article has not been accessed online) ---

1984 Modeer 1984
doi.org/10.1111/j.1600-0722.1984.tb00908.x
Modeer T, Twetman S, Bergstrand F. Three-year study of the effect of fluoride varnish (Duraphat) on proximal caries progression in teenagers. Scandinavian Journal of Dental Research 1984;92:400-7.
0 Ethics committee not mentioned

1979 Holm 1979
doi.org/10.1111/j.1600-0528.1979.tb01225.x
Holm AK. Effect of fluoride varnish (Duraphat) in preschool children. Community Dentistry and Oral Epidemiology 1979;7:241-5.
--- (article has not been accessed online) ---

1975 Koch 1975
doi.org/10.1111/j.1600-0528.1975.tb00321.x
Koch G, Petersson LG. Caries preventive effect of a fluoride-containing varnish (Duraphat) after 1 year's study. Community Dentistry and Oral Epidemiology 1975;3:262-6.
--- (article has not been accessed online) ---

McMillan R, Forssell H, Buchanan JA, Glenny AM, Weldon JC, Zakrzewska JM http://doi.org/10.1002/14651858.CD002779.pub3
2016 Interventions for treating burning mouth syndrome Primary studies: 23

2015 Palacios-Sánche
doi.org/10.4317%2Fmedoral.20410
Palacios-Sánchez B, Moreno-López LA, Cerero-Lapiedra R, Llamas-Martínez S, Esparza-Gómez G. Alpha lipoic acid efficacy in burning mouth syndrome. A controlled clinical trial. Medicina Oral, Patología Oral y Cirugía Bucal 2015;20(4):e435-40.
1 approved by the Ethical Committee of the Hospital Clínico San Carlos of Madrid

Cochrane_Author		Primary studies_	doi
2014	Alvarenga da Sil	Alvarenga da Silva L, Siqueira JT, Teixeira MJ, Siqueira SR. The role of xerostomia in burning mouth syndrome: a case-control study. Arquivos de Neuro-psiquiatria 2014;72(2):91-8	doi.org/10.1590%2F0004-282X20130218
		1 approved by the local Ethics Committee	
2014	Cano-Carrillo 20	Cano-Carrillo P, Pons-Fuster A, López-Jornet P. Efficacy of lycopene-enriched virgin olive oil for treating burning mouth syndrome: a double-blind randomised. Journal of Oral Rehabilitation 2014;41(4):296-305	doi.org/10.1111/joor.12147
		1 approved by the Ethics Committee of the University of Murcia	
2012	Heckmann 201	Heckmann SM, Kirchner E, Grushka M, Wichmann MG, Hummel T. A double-blind study on clonazepam in patients with burning mouth syndrome. Laryngoscope 2012;122(4):813-6.	doi.org/10.1002/lary.22490
		1 approved by the ethics committee of the University of Erlangen Medical Schoo	
2012	Silvestre 2012	Silvestre FJ, Silvestre-Rangil J, Tamarit-Santafé C, Bautista D. Application of a capsaicin rinse in the treatment of burning mouth syndrome. Medicina Oral, Patología Oral y Cirugía Bucal 2012;17(1):e1-4	doi.org/10.4317%2Fmedoral.17219
		1 approved by the Clinical Research Ethics Committee of Dr. Peset University Hospital (Valencia, Spain)	
2012	Spanemberg 20	Spanemberg JC, Cherubini K, de Figueiredo MA, Gomes AP, Campos MM, Salum FG. Effect of an herbal compound for treatment of burning mouth syndrome: randomized, controlled, double-blind clinical trial. Oral Surgery, Oral Medicine, Oral Pathology, Oral Radiology, and Endodontics 2012;113(3):373-7	doi.org/10.1016%2Fj.oooo.2011.09.005
		2 approved by the Ethics in Research Committee of the Pontifical Catholic University of Rio Grande do Sul (PUCRS; 09/04817) and local committees	
2011	Lopez-D'alessan	López-D'alessandro E, Escovich L. Combination of alpha lipoic acid and gabapentin, its efficacy in the treatment of Burning Mouth Syndrome: a randomized, double-blind, placebo controlled trial. Medicina Oral, Patología Oral y Cirugía Bucal 2011;16(5):e635-40	doi.org/10.4317%2Fmedoral.16942
		1 approved by the Bioethics Committee of the Institution.	
2011	López-Jornet 20	López-Jornet P, Camacho-Alonso F, Andujar-Mateos P. A prospective, randomized study on the efficacy of tongue protector in patients with burning mouth syndrome. Oral Diseases 2011;17(3):277-82.	doi.org/10.1111/j.1601-0825.2010.01737.x
		1 approved by the Bioethics Committee of the University of Murcia	
2010	Marino 2010	Marino R, Torretta S, Capaccio P, Pignataro L, Spadari F. Different therapeutic strategies for burning mouth syndrome: preliminary data. Journal of Oral Pathology & Medicine 2010;39(8):611-6.	doi.org/10.1111/j.1601-0825.2010.01737.x
		1 approved by the Local Ethics Committee	
2010	Rodríguez de Ri	Rodríguez de Rivera-Campillo E, López-López J, Chimenos-Küstner E. Response to topical clonazepam in patients with burning mouth syndrome: a clinical study. Bulletin de Groupement Europeen pour la Recherche Scientifique en Stomatologie et Odontologie 2010;49(1):19-29	ncbi.nlm.nih.gov/pubmed/22750263
		--- (article has not been accessed online) ---	

Cochrane_Author		Primary studies_	doi
2009	Carbone 2009	Carbone M, Pentenero M, Carrozzo M, Ippolito A, Gandolfo S. Lack of efficacy of alpha-lipoic acid in burning mouth syndrome: a double-blind, randomized, placebo-controlled study. European Journal of Pain 2009;13(5):492-6	
doi.org/10.1016/j.ejpain.2008.06.004		1 approved by the local Ethics Committee	
2009	Cavalcanti 2009	Cavalcanti DR, da Silveira FR. Alpha lipoic acid in burning mouth syndrome - a randomized double-blind placebo-controlled trial. Journal of Oral Pathology & Medicine 2009;38(3):254-61	
doi.org/10.1111/j.1600-0714.2008.00735.x		1 approved by The Ethical Committee in Human Research of The Dentistry School, University of São Paulo, São Paulo, Brazi	
2009	López-Jornet 20	López-Jornet P, Camacho-Alonso F, Leon-Espinosa S. Efficacy of alpha lipoic acid in burning mouth syndrome: a randomized, placebo-treatment study. Journal of Oral Rehabilitation 2009;36(1):52-7	
doi.org/10.1111/j.1365-2842.2008.01914.x		1 approved by the Bioethics Committee of the University of Murcia	
2008	Sardella 2008	Sardella A, Lodi G, Demarosi F, Tarozzi M, Canegallo L, Carrassi A. Hypericum perforatum extract in burning mouth syndrome: a randomized placebo-controlled study. Journal of Oral Pathology & Medicine 2008;37(7):395-401	
doi.org/10.1111/j.1600-0714.2008.00663.x		1 approved by the local University Hospital Ethics Committee	
2004	Grémeau-Richa	Grémeau-Richard C, Woda A, Navez ML, Attal N, Bouhassira D, Gagnieu MC, et al. Topical clonazepam in stomatodynia: a randomised placebo-controlled study. Pain 2004;108(1-2):51-7.	
doi.org/10.1016%2Fj.pain.2003.12.002		1 approved by the local Ethics Committe	
2002	Femiano 2002a	Femiano F, Scully C. Burning mouth syndrome (BMS): double blind controlled study of alpha-lipoic acid (thioctic acid) therapy. Journal of Oral Pathology & Medicine 2002;31(5):267-9	
doi.org/10.1034/j.1600-0714.2002.310503.x		0 Ethics committee not mentioned	
2002	Femiano 2002b	Femiano F. Burning mouth syndrome (BMS): an open trial of comparative efficacy of alpha-lipoic acid (thioctic acid) with other therapies. Minerva Stomatologica 2002;51(9):405-9	
ncbi.nlm.nih.gov/pubmed/12473978		--- (article has not been accessed online) ---	
2000	Femiano 2000	Femiano F, Gombos F, Scully C, Busciolano M, De Luca P. Burning mouth syndrome (BMS): controlled open trial of the efficacy of alpha-lipoic acid (thioctic acid) on symptomatology. Oral Diseases 2000;6(5):274-7.	
doi.org/10.1111/j.1601-0825.2000.tb00138.x		1 approved by the Local ethical committee	
1999	Bogetto 1999	Bogetto F, Bonatto Revello R, Ferro G, Maina L, Ravizza L. Trattamento psicofarmacologico della burning mouth syndrome (BMS). Studio su di un campione di 121 pazienti. Minerva Psichiatrica 1999;40(1):1-10	
		--- (article has not been accessed online) ---	

1999 Sardella 1999
doi.org/10.1016%2FS1079-2104%2899%2970010-7
Sardella A, Uglietti D, Demarosi F, Lodi G, Bez C, Carrassi A. Benzydamine hydrochloride oral rinses in management of burning mouth syndrome. A clinical trial. Oral Surgery, Oral Medicine, Oral Pathology, Oral Radiology, and Endodontics 1999;88(6):683-6
1 approved by the University Ethics Committee

1999 Tammiala-Salon
ncbi.nlm.nih.gov/pubmed/10425979
Tammiala-Salonen T, Forssell H. Trazodone in burning mouth pain: a placebo-controlled, double-blind study. Journal of Orofacial Pain 1999;13(2):83-8
1 approved by the joint commission on ethics of Turku university and Turku university central hospital

1995 Bergdahl 1995a
doi.org/10.1111/j.1600-0714.1995.tb01169.x
Bergdahl J, Anneroth G, Perris H. Cognitive therapy in the treatment of patients with resistant burning mouth syndrome: a controlled study. Journal of Oral Pathology & Medicine 1995;24(5):213-5
--- (article has not been accessed online) ---

Millett DT, Glenny AM, Mattick RC, Hickman J, Mandall NA http://doi.org/10.1002/14651858.CD004485.pub4
2016 Adhesives for fixed orthodontic bands Primary studies: 8

2005 Williams 2005
doi.org/10.1093%2Fejo%2Fcji009
Williams PH, Sherriff M, Ireland AJ. An investigation into the use of two polyacid-modified composite resins (compomers) and a resin-modified glass poly(alkenoate) cement used to retain orthodontic bands. European Journal of Orthodontics 2005;27:245-51
1 approved by the Ethics committee

2003 Clark 2003
doi.org/10.1093%2Fejo%2F25.3.319
Clark JR, Ireland AJ, Sherriff M. An in vivo and ex vivo study to evaluate the use of a glass polyphosphonate cement in orthodontic banding. European Journal of Orthodontics 2003;25(3):319-23
1 approved by the local ethics committee

2003 Galarraga 2003
ortodoncia.ws/publicaciones/2003/art-3/
Galarraga NR, Croce N. Estudio clínico comparativo entre el cementado de bandas ortodóncicas con fosfato de zinc y con vidrio ionomérico convencional]. Latin American Journal of Orthodontics and Pediatric Dentistry
--- (article has not been accessed online) ---

2001 Gillgrass 2001
Gillgrass TJ, Benington PC, Millett DT, Newell J, Gilmour WH. Modified composite or conventional glass ionomer for band cementation? A comparative clinical trial. American Journal of Orthodontics and Dentofacial Orthopedics 2001;120(1):49-53.
--- (article has not been accessed online) ---

1997 Fricker 1997
doi.org/10.1016%2FS0889-5406%2897%2970250-6
Fricker JP. A 12-month clinical comparison of resin-modified light-activated adhesives for the cementation of orthodontic molar bands. American Journal of Orthodontics and Dentofacial Orthopedics 1997;112(3):239-43
0 Ethics committee not mentioned

1991 Stirrups 1991 Stirrups DR. A comparative clinical trial of a glass ionomer and a zinc phosphate cement for securing orthodontic bands. British Journal of Orthodontics 1991;18(1):15-20

doi.org/10.1179%2Fbjo.18.1.15

--- (article has not been accessed online) ---

1989 Durning 1989 Durning P. A clinical and laboratory investigation into the cements used to retain orthodontic bands [dissertation]. Newcastle-upon-Tyne, UK: University of Newcastle-upon-Tyne, 1989.

--- (article has not been accessed online) ---

1983 Kvam 1983 Kvam E, Broch J, Nissen-Meyer IH. Comparison between a zinc phosphate cement and a glass ionomer cement for cementation of orthodontic bands. European Journal of Orthodontics 1983;5(4):307-13

doi.org/10.1093%2Fejo%2F5.4.307

--- (article has not been accessed online) ---

Millett DT, Mandall NA, Mattick RC, Hickman J, Glenny AM http://doi.org/10.1002/14651858.CD008236.pub3

2017 Adhesives for bonded molar tubes during fixed brace treatment Primary studies: 2

2011 Nazir 2011 Nazir M, Walsh T, Mandall NA, Matthew S, Fox D. Banding versus bonding of first permanent molars: a multi-centre randomized controlled trial. Journal of Orthodontics 2011;38(2):81-9

doi.org/10.1179%2F14653121141308

1 approved by the NHS Multi-Centre Research Ethics Committee.

2007 Banks 2007 Banks P, Macfarlane TV. Bonded versus banded first molar attachments: a randomized controlled clinical trial. Journal of Orthodontics 2007;34(2):128-36.

doi.org/10.1179%2F146531207225022032

2 approved by the Burnley Pendle and Rossendale (refLRECBPR 299), and Bury and Rochdale (refBRLREC 225) Local Research Ethics Committees

Montedori A, Abraha I, Orso M, D'Errico PG, Pagano S, Lombardo G http://doi.org/10.1002/14651858.CD010229.pub2

2016 Lasers for caries removal in deciduous and permanent teeth Primary studies: 9

2014 Belcheva 2014 Belcheva A, Shindova M. Subjective acceptance of pediatric patients during cavity preparation with Er:YAG laser and conventional rotary instruments. Journal of IMAB 2014;20(5):631-3.

doi.org/10.5272/jimab.2014205.631

0 Ethics committee not mentioned

2013 Zhang 2013 Zhang S, Chen T, Ge LH. Evaluation of clinical outcomes for Er:YAG laser application in caries therapy of children. Beijing Da Xue Xue Bao (Journal of Peking University-Health Sciences) 2013;45(1):87-91.

--- (article has not been accessed online) ---

2010 Yazim 2010 Yazici AR, Baseren M, Gorucu J. Clinical comparison of bur- and laser-prepared minimally invasive occlusal resin composite restorations: two-year follow-up. Operative Dentistry 2010;35(5):500-7.

doi.org/10.2341%2F09-339-C

1 approved by the Ethics Committee of Hacettepe University

Cochrane_Author	Primary studies_	doi
2006 Liu 2006 doi.org/10.1089%2Fpho.2006.24.489	Liu JF, Lai YL, Shu WY, Lee SY. Acceptance and efficiency of Er:YAG laser for cavity preparation in children. Photomedicine and Laser Surgery 2006;24(4):489-93.	
	2 approved by the ethics committee of Taichung Veterans General Hospital (IRBTCVGH–940329/536)	
2001 Denbesten 200 doi.org/10.1078%2F1615-1615-00025	DenBesten PK, White JM, Pelino JEP, Furnish G, Silveira A, Parkins FM. The safety and effectiveness of an Er:YAG laser for caries removal and cavity preparation in children. Medical Laser Application 2001;16(3):215-22.	
	1 approved by the human subjects institutional review board at each site	
2000 Evans 2000 doi.org/10.1038/sj.bdj.4800575a	Evans DJ, Matthews S, Pitts NB, Longbottom C, Nugent ZJ. A clinical evaluation of an Erbium:YAG laser for dental cavity preparation. British Dental Journal 2000;188(12):677-9.	
	1 approved by the local medical research ethics committee	
2000 Hadley 2000 doi.org/10.14219%2Fjada.archive.2000.0277	Hadley J, Young DA, Eversole LR, Gornbein JA. A laser-powered hydrokinetic system for caries removal and cavity preparation. Journal of the American Dental Association 2000;131(6):777-85.	
	1 approved by the Institutional Review Board on the use of humans for clinical research at the University of the Pacific	
2000 Harris 2000 doi.org/10.1117%2F12.380826	Harris DM, Goodis H, White J, Arcoria C, Simon J, Burkart J, et al. Pulsed Nd: YAG laser selective ablation of surface enamel caries: II. Histology and clinical trials. Lasers in Dentistry VI 2000;3910:184-92.	
	--- (article has not been accessed online) ---	
1998 Keller 1998 doi.org/10.1016%2FS0300-5712%2897%2900036-5	Keller U, Hibst R, Geurtsen W, Schilke R, Heidemann D, Klaiber B, et al. Erbium:YAG laser application in caries therapy. Evaluation of patient perception and acceptance. Journal of Dentistry 1998;26(8):649-56.	
	1 approved by the Ethics Committees responsible for the participating universities.	

Nagraj SK, Naresh S, Srinivas K, Renjith George P, Shrestha A, Levenson D, Fer — http://doi.org/10.1002/14651858.CD010470.pub2

2014 Interventions for the management of taste disturbances — Primary studies: 9

2013 Ikeda 2013 doi.org/10.3950%2Fjibiinkoka.116.17	Ikeda M, Kurono Y, Inokuchi A, Takeda N, Aiba T, Nomura Y, et al. [The effect of zinc agent in 219 patients with zinc deficiency-inductive/Idiopathic taste disorder: A placebo controlled randomized study]. Nippon Jibiinkoka Gakkai Kaiho (Tokyo) 2013;116:17-26.	
	--- (article has not been accessed online) ---	
2009 Sakagami 2009 doi.org/10.1080%2F00016480802552550	Sakagami M, Ikeda M, Tomita H, Ikui A, Aiba T, Takeda N, et al. A zinc-containing compound, Polaprezinc, is effective for patients with taste disorders: randomized, double-blind, placebo-controlled, multi-center study. Acta Oto-Laryngologica 2009;129(10):1115-20.	
	1 approved by the Institutional Review Boards of the participating institutions	

2008	Brandt 2008	doi.org/10.1016%2FS1887-8369%2808%2972005-2	Brandt H, Hauswald B, Langer H, Gleditsch J, Zahnert T. Efficacy of acupuncture in the treatment of idiopathic taste disorders [Efficacia de la acupuntura para el tratamiento de los trastornos idiopaticos del sentido del gusto]. Revista Internacional de Acupuntura 2008;2:155-62.<br>--- (article has not been accessed online) ---
2005	Heckmann 200	doi.org/10.1177%2F154405910508400105	Heckmann SM, Hujoel P, Habiger S, Friess W, Wichmann M, Heckmann JG, et al. Zinc gluconate in the treatment of dysgeusia—a randomized clinical trial. Journal of Dental Research 2005;84(1):35-8.<br>2 approved by the ethics committee of the University of Erlangen-Nuremberg (Nr. 2266)
2002	Sakai 2002	doi.org/10.1080%2F00016480260046517	Sakai F, Yoshida S, Endo S, Tomita H. Double-blind, placebo-controlled trial of zinc picolinate for taste disorders. Acta Oto-Laryngologica 2002;Suppl 546:129-33.<br>0 Ethics committee not mentioned
1983	Watson 1983	ncbi.nlm.nih.gov/pubmed/6347985	Watson AR, Stuart A, Wells FE, Houstan IB, Addison GM. Zinc supplementation and its effect on taste acuity in children with chronic renal failure. Human Nutrition: Clinical Nutrition 1983;37(3):219-25.<br>--- (article has not been accessed online) ---
1982	Eggert 1982	ncbi.nlm.nih.gov/pubmed/7107115	Eggert JV, Siegler RL, Edomkesmalee E. Zinc supplementation in chronic renal failure. The International Journal of Pediatric Nephrology 1982;3(1):21-4.<br>--- (article has not been accessed online) ---
1982	Mahajan 1982	ncbi.nlm.nih.gov/pubmed/6890761	Mahajan SK, Prasad AS, Rabbani P, Briggs WA, McDonald FD. Zinc deficiency: a reversible complication of uremia. The American Journal of Clinical Nutrition 1982;36(6):1177-83.<br>--- (article has not been accessed online) ---
1980	Mahajan 1980	ncbi.nlm.nih.gov/pubmed/6772011	Mahajan SK, Prasad AS, Lambujon J, Abbasi AA, Briggs WA, McDonald FD. Improvement of uremic hypogeusia by zinc: a double-blind study. The American Journal of Clinical Nutrition 1980;33(7):1517-21.<br>--- (article has not been accessed online) ---

Nasser M, Pandis N, Fleming PS, Fedorowicz Z, Ellis E, Ali K http://doi.org/10.1002/14651858.CD006087.pub3

2013 Interventions for the management of mandibular fractures Primary studies: 12

2012	Emam 2012	doi.org/10.1016%2Fj.joms.2011.08.010	Emam HA, Stevens MR. Can an arch bar replace a second lag screw in management of anterior mandibular fractures?. Journal of Oral and Maxillofacial Surgery 2012;70(2):378-83.<br>1 approved by the graduate advisor committee for humans at Cairo University

Cochrane_Author		Primary studies_	doi
2012	Gupta 2012	Gupta A, Singh V, Mohammad S. Bite force evaluation of mandibular fractures treated with microplates and miniplates. Journal of Oral and Maxillofacial Surgery 2012;70(8):1903-8.	doi.org/10.1016%2Fj.joms.2012.03.036
		1 approved by the institutional ethical committee	
2012	Singh 2012	Singh V, Puri P, Arya S, Malik S, Bhagol A. Conventional versus 3-dimensional miniplate in management of mandibular fracture: a prospective randomized study. Otolaryngology - Head and Neck Surgery 2012;147(3):450-5.	doi.org/10.1177%2F0194599812449437
		1 approved by the institutional review board (Post Graduate Institute of Dental Sciences)	
2011	Agarwal 2011	Agarwal M, Mohammad S, Singh RK, Singh V. Prospective randomized clinical trial comparing bite force in 2-mm locking plates versus 2-mm standard plates in treatment of mandibular fractures. Journal of Oral and Maxillofacial Surgery 2011;69(7):1995-2000.	doi.org/10.1016%2Fj.joms.2010.10.014
		1 approved by our local local ethics board	
2010	Bhatt 2010	Bhatt K, Roychoudhury A, Bhutia O, Trikha A, Seith A, Pandey RM. Equivalence randomized controlled trial of bioresorbable versus titanium miniplates in treatment of mandibular fracture: a pilot study. Journal of Oral and Maxillofacial Surgery 2010;68(8):1842-8.	doi.org/10.1016%2Fj.joms.2009.09.005
		2 approved by the all India institute of Medical Sciences ethics committee (# A-54/25.07.2007)	
2010	Jain 2010	Jain MK, Manjunath KS, Bhagwan BK, Shah DK. Comparison of 3-dimensional and standard miniplate fixation in the management of mandibular fractures. Journal of Oral and Maxillofacial Surgery 2010;68(7):1568-72.	doi.org/10.1016%2Fj.joms.2009.07.083
		1 approved by a ethics and research committee	
2009	Sugar 2009	Sugar AW, Gibbons AJ, Patton DW, Silvester KC, Hodder SC, Gray M, et al. A randomised controlled trial comparing fixation of mandibular angle fractures with a single miniplate placed either transbuccally and intra-orally, or intra-orally alone. International Journal of Oral and Maxillofacial Surgery 2009;38(3):241-5.	doi.org/10.1016%2Fj.ijom.2008.11.001
		2 approved by the Iechyd Morgannwg Local Research Ethics Committee, No. 2000.001	
2008	Pigadas 2008	Pigadas N, Whitley SP, Roberts SA, McAlister K, Ameerally P, Avery CM. A randomized controlled trial on cross-infection control in maxillofacial trauma surgery: a comparison of intermaxillary fixation techniques. International Journal of Oral and Maxillofacial Surgery 2008;37(8):716-22.	doi.org/10.1016%2Fj.ijom.2008.05.005
		1 approved by the Central, South Manchester and Leicester Local Research Ethics Committees	
2007	Siddiqui 2007	Siddiqui A, Markose G, Moos KF, McMahon J, Ayoub AF. One miniplate versus two in the management of mandibular angle fractures: a prospective randomised study. The British Journal of Oral and Maxillofacial Surgery 2007;45(3):223-5.	doi.org/10.1016%2Fj.bjoms.2006.08.016
		1 approved by the ethics committee	
2004	Collins 2004	Collins CP, Pirinjian-Leonard G, Tolas A, Alcalde R. A prospective randomized clinical trial comparing 2.0-mm locking plates to 2.0-mm standard plates in treatment of mandible fractures. Journal of Oral and Maxillofacial Surgery 2004;62(11):1392-5.	doi.org/10.1016%2Fj.joms.2004.04.020
		0 Ethics committee not mentioned	

2001	Kaplan 2001	Kaplan BA, Hoard MA, Park SS. Immediate mobilization following fixation of mandible fractures: a prospective, randomized study. Laryngoscope 2001;111(9):1520-4.	
doi.org/10.1097/00005537-200109000-00006			0 Ethics committee not mentioned
1997	Schierle 1997	Schierle HP, Schmelzeisen R, Rahn B, Pytlik C. One- or two-plate fixation of mandibular angle fractures?. Journal of Cranio-Maxillo-Facial Surgery 1997;25(3):162-8.	
doi.org/10.1016%2FS1010-5182%2897%2980009-1			0 Ethics committee not mentioned

Parekh S, Gardener C, Ashley PF, Walsh T http://doi.org/10.1002/14651858.CD009742.pub2

2014 Intraoperative local anaesthesia for reduction of postoperative pain following general anaesthesia for dental treatmen Primary studies: 14

2009	Townsend 2009	Townsend JA, Ganzberg S, Thikkurissy S. The effect of local anesthetic on quality of recovery characteristics following dental rehabilitation under general anesthesia in children. Anesthesia Progress 2009;56(4):115-2	
doi.org/10.2344%2F0003-3006-56.4.115			--- (article has not been accessed online) ---
2009	Watts 2009	Watts AK, Thikkurissy S, Smiley M, McTigue DJ, Smith T. Local anesthesia affects physiologic parameters and reduces anesthesiologist intervention in children undergoing general anesthesia for dental rehabilitation. Pediatric Dentistry 2009;31(5):414-9.	
ingentaconnect.com/contentone/aapd/pd/2009/00000031/00000005/art00009			1 approved by the IRB
2007	Leong 2007	Leong KJ, Roberts GJ, Ashley PF. Perioperative local anaesthetic in young paediatric patients undergoing extractions under outpatient 'short-case' general anaesthesia. A double-blind randomised controlled trial. British Dental Journal 2007;203(6):E11	
doi.org/10.1038%2Fbdj.2007.724			1 approved by the Eastman Dental Institute/Eastman Dental Hospital Joint Research and Ethics Committee,R&D University College London Hospitals
2007	McWilliams 200	McWilliams PA, Rutherford JS. Assessment of early postoperative pain and haemorrhage in young children undergoing dental extractions under general anaesthesia. International Journal of Paediatric Dentistry 2007;17(5):352-7	
doi.org/10.1111/j.1365-263X.2007.00841.x			1 approved by Local ethics committee
2007	Sammons 2007	Sammons HM, Unsworth V, Gray C, Choonara I, Cherrill J, Quirke W. Randmoized controlled trial of the intraligamental use of a local anaesthetic (lignocaine 2%) versus controls in paediatric tooth extraction. International Journal of Paediatric Dentistry 2007;17(4):297-303	
doi.org/10.1111/j.1365-263X.2007.00832.x			1 approved by the Southern Derbyshire Local Research Ethics Committee
2006	Coulthard 2006	Coulthard P, Rolfe S, Mackie IC, Gazal G, Morton M, Jackson-Leech D. Intraoperative local anaesthesia for paediatric postoperative oral surgery pain - a randomized controlled trial. International Journal of Oral Maxillofacial Surgery 2006;35(12):1114-9	
doi.org/10.1016%2Fj.ijom.2006.07.007			1 approved by the Central Manchester and Manchester Children's University Hospitals NHS Trust Research Ethics Committee

Year	Study	Reference	Ethics
2005	Anand 2005 http://admin.ejpd.eu/download/2005-01-01.pdf	Anand P, Wilson R, Sheehy EC. Intraligamental analgesia for post-operative pain control in children having dental extractions under general anaesthesia. European Journal of Paediatric Dentistry 2005;6(1):10-5	1 approved by local ethics committee
2005	Quirke 2005 doi.org/10.1185%2F146300905X65678	Quirke W, Bhaskar K, Choonara I. A clinical trial of topical application of bupivacaine to reduce post-operative pain in children following dental extractions. Pediatric and Perinatal Drug Therapy 2005;6(4):197-9.	--- (article has not been accessed online) ---
2004	Gazal 2004 doi.org/10.1111/j.1365-263X.2004.00587.x	Gazal G, Bowman R, Worthington HV, Mackie IC. A double-blind randomized controlled trial investigating the effectiveness of topical bupivacaine in reducing distress in children following extractions under general anaesthesia. International Journal of Paediatric Dentistry 2004;14(6):425-31.	1 approved by the Central Manchester Local Research Ethics Committee
2002	Andrzejowski 2 doi.org/10.1046/j.1365-2044.2002.2408_5.x	Andrzejowski J, Lamb L. The effect of swabs soaked in bupivacaine and epinephrine for pain relief following simple dental extractions in children. Anaesthesia 2002;57(3):281-3	1 approved by our regional ethics committee
2001	Al-Bahlani 2001 ncbi.nlm.nih.gov/pubmed/11697691	AL-Bahlani S, Sherriff A, Crawford PJM. Tooth extraction, bleeding and pain control. Journal of the Royal College of Surgeons of Edinburgh 2001;46(5):261-4	--- (article has not been accessed online) ---
1994	Noble 1994	Noble DW, Raab, GM, MacLean D, MacLachan D. Prilocaine infiltration as postoperative analgesia for children having dental extractions under general anesthesia. Regional Anesthesia 1994;19(2):126-31.	--- (article has not been accessed online) ---
1993	Elhakim 1993 ncbi.nlm.nih.gov/pubmed/8216665	Elhakim M. Painless dental extraction in children. Anaesthesiologie und Reanimation 1993;18(3):80-2	--- (article has not been accessed online) ---
1990	Rashad 1990 doi.org/10.1016%2F0266-4356%2890%2990132-5	Rashad A, El-Attar A. Cardiac dysrhythmias during oral surgery: effect of combined local and general anaesthesia. British Journal of Oral and Maxillofacial Surgery 1990;28(2):102-4.	0 Ethics committee not mentioned

Pereira-Cenci T, Cenci MS, Fedorowicz Z, Azevedo M http://doi.org/10.1002/14651858.CD007819.pub3

2013 Antibacterial agents in composite restorations for the prevention of dental caries Primary studies: 0

EMPTY Empty Systematic Review

Poklepovic T, Worthington HV, Johnson TM, Sambunjak D, Imai P, Clarkson J http://doi.org/10.1002/14651858.CD009857.pub2

2013 Interdental brushing for the prevention and control of periodontal diseases and dental caries in adults Primary studies: 7

2011 Imai 2011 Imai PH, Hatzimanolakis PC. Interdental brush in type I embrasures: examiner blinded randomized clinical trial of bleeding and plaque efficacy. Canadian Journal of Dental Hygiene 2011;45(1):13-20.

oralscience.com/floss-interdental-cdjh.pdf

1 approved by the University of British Columbia Clinical Research Ethics Committee in Vancouver, Canada

2007 Ishak 2007 Ishak N, Watts TLP. A comparison of the efficacy and ease of use of dental floss and interproximal brushes in a randomised split mouth trial incorporating an assessment of subgingival plaque. Oral Health & Preventive Dentistry 2007;5(1):13-8.

quintpub.com/journals/ohpd/fulltext.php?article_id=9071

1 approved by the Guy's and St Thomas hospitals ethics committee

2006 Jackson 2006 Jackson MA, Kellett M, Worthington HV, Clerehugh V. Comparison of interdental cleaning methods: a randomized controlled trial. Journal of Periodontology 2006;77(8):1421-9.

doi.org/10.1902%2Fjop.2006.050360

1 approved by the Local Research Ethics Committee

2006 Yost 2006 Yost KG, Mallatt ME, Liebman J. Interproximal gingivitis and plaque reduction by four interdental products. Journal of Clinical Dentistry 2006;17(3):79-83.

ncbi.nlm.nih.gov/pubmed/17022370

--- (article has not been accessed online) ---

2005 Jared 2005 Jared H, Zhong Y, Rowe M, Ebisutani K, Tanaka T, Takase N. Clinical trial of a novel interdental brush cleaning system. Journal of Clinical Dentistry 2005;16(2):47-52.

ncbi.nlm.nih.gov/pubmed/16170976

--- (article has not been accessed online) ---

2002 Yankell 2002 Yankell SL, Shi X, Emling RC. Efficacy and safety of BrushPicks, a new cleaning aid, compared to the use of Glide floss. Journal of Clinical Dentistry 2002;13(3):125-9.

ncbi.nlm.nih.gov/pubmed/11887516

--- (article has not been accessed online) ---

1998 Christou 1998 Christou V, Timmerman MF, Van der Velden U, Van der Weijden FA. Comparison of different approaches of interdental oral hygiene: interdental brushes versus dental floss. Journal of Periodontology 1998;69(7):759-64.

doi.org/10.1902%2Fjop.1998.69.7.759

0 Ethics committee not mentioned

Prashanti E, Sumanth KN, Renjith George P, Karanth L, Soe HH http://doi.org/10.1002/14651858.CD011116.pub2

2015 Management of gag reflex for patients undergoing dental treatment Primary studies: 1

2014	Zotelli 2014 doi.org/10.1016%2Fj.jams.2014.04.009	Zotelli VLR, Grillo CM, Rosario de Sousa ML. Nausea control by needling at acupuncture point Neiguan (PC6) during an intraoral impression-taking procedure. Journal of Acupuncture and Meridian Studies 2014;7(6):318-23.	2 approved by the Ethics Committee on Research of the FOP-UNICAMP (116/2012)

Rasines Alcaraz MG, Veitz-Keenan A, Sahrmann P, Schmidlin PR, Davis D, Iheo http://doi.org/10.1002/14651858.CD005620.pub2

2014 Direct composite resin fillings versus amalgam fillings for permanent or adult posterior teeth — Primary studies: 7

2007	Casa Pia 2007 doi.org/10.14219%2Fjada.archive.2007.0265	Bernardo M, Luis H, Martin MD, Leroux BG, Rue T, Leitão J, et al. Survival and reasons for failure of amalgam versus composite posterior restorations placed in a randomized clinical trial. Journal of the American Dental Association 2007;138(6):775-83.	1 approved by the institutional review boards of the University of Washington and the University of Lisbon Faculty of Dental Medicine
2007	NECAT 2007 doi.org/10.14219%2Fjada.archive.2007.0264	Soncini JA, Maserejian NN, Trachtenberg F, Tavares M, Hayes C. The longevity of amalgam versus compomer/composite restorations in posterior primary and permanent teeth: findings from the New England Children's Amalgam Trial. Journal of the American Dental Association 2007;138(6):763-7	1 approved by the institutional review boards of the New England Research Institutes, The Forsyth Institute, Boston; and all participating dental clinics
1990	Cunningham 19 [doi.org/10.1038%2Fsj.bdj.4807369](doi.org/10.1038%2Fsj.bdj.4807369)	Cunningham J, Mair LH, Foster MA, Ireland RS. Clinical evaluation of three posterior composite and two amalgam restorative materials: 3-year results. British Dental Journal 1990;169(10):319-23	--- (article has not been accessed online) ---
1990	Norman 1990 [doi.org/10.1016%2F0022-3913%2890%2990121-R](doi.org/10.1016%2F0022-3913%2890%2990121-R)	Norman RD, Wright JS, Rydberg RJ, Felkner LL. A 5-year study comparing a posterior composite resin and an amalgam. Journal of Prosthetic Dentistry 1990;64(5):523-9	0 Ethics committee not mentioned
1989	Letzel 1989 [doi.org/10.1016%2F0300-5712%2889%2990156-5](doi.org/10.1016%2F0300-5712%2889%2990156-5)	Letzel H. Survival rates and reasons for failure of posterior composite restorations in multicentre clinical trial. Journal of Dentistry 1989;17(Suppl 1):S10-7.	0 Ethics committee not mentioned
1988	Robinson 1988 [doi.org/10.1111/j.1834-7819.1994.tb01377.x](doi.org/10.1111/j.1834-7819.1994.tb01377.x)	Robinson AA, Rowe AH, Maberley ML. A three-year study of the clinical performance of a posterior composite and a lathe cut amalgam alloy. British Dental Journal 1988;164(8):248-52.	--- (article has not been accessed online) ---
1986	Hendriks 1986 [doi.org/10.1111/j.1365-2842.1986.tb01302.x](doi.org/10.1111/j.1365-2842.1986.tb01302.x)	Hendriks FH, Letzel H, Vrijhoef MM. Composite versus amalgam restorations. A three-year clinical evaluation. Journal of Oral Rehabilitation 1986;13(5):401-1	0 Ethics committee not mentioned

Ren Q, Yan X, Zhou Y, Li WX http://doi.org/10.1002/14651858.CD009477.pub2

2016 Periodontal therapy as adjunctive treatment for gastric Helicobacter pylori infection — Primary studies: 7

2014 Wang 2014 ncbi.nlm.nih.gov/pubmed/25179088	Wang XM, Yee KC, Hazeki-Taylor N, Li J, Fu HY, Huang ML, et al. Oral Helicobacter pylori, its relationship to successful eradication of gastric H. pylori and saliva culture confirmation. Journal of Physiology and Pharmacology 2014;65(4):559-66.	1 approved by the ethical committee of the hospital
2012 Liu 2012	Liu Z, Meng WD, Wang ZK. Effect of dental plaque on eradication rate of gastric Helicobacter pylori in patients with peptic ulcer. Zhongguo Wuzhenxue Zazhi [Chinese Journal of Misdiagnostics] 2012;12(5):1038.	--- (article has not been accessed online) ---
2009 Jia 2009	Jia CL, Jiang GS, Yang XX, Dou HQ, Li CR. Effects onHelicobacter pylori reinfection in gastric mucosa by two oral plaque control methods. Huaxi Kongqiang Yixue Zazhi [West China Journal of Stomatology] 2009;27(2):172-4.	--- (article has not been accessed online) ---
2009 Zaric 2009 [doi.org/10.1177%2F0022034509344559](doi.org/10.1177%2F0022034509344559)	Zaric S, Bojic B, Jankovic Lj, Dapcevic B, Popovic B, Cakic S, et al. Periodontal therapy improves gastric Helicobacter pylori eradication. Journal of Dental Research 2009;88(10):946-50.	1 approved by the Ethical Committee of the School of Dentistry, University of Belgrade.
2007 Jin 2007	Jin F, Yuan F, Zhang ZJ, Le QL, Zhu JZ. Effect of eradication treatment associated with initial periodontal therapy on the eradication rate of gastric Helicobacter pylori. Shiyong Yixue Zazhi [The Journal of Practical Medicine] 2007;23(5):677-8.	--- (article has not been accessed online) ---
2006 Lv 2006	Lv X, Yao BT. The effect on Hp relative gastrosia with Rhizoma Coptidis aerosol clearing away the Helicobacter pylori in the dental plaque. Zhonghua Linchuangyixue Zazhi [Chinese Journal of Clinical Practical Medicine] 2006;7(2):28-30.	--- (article has not been accessed online) ---
2003 Jin 2003 en.cnki.com.cn/Article_en/CJFDTotal-ZWZX200310026.htm	Jin F, Yuan F, Jiang HL, Huang XQ, Ma HM, Le QL, et al. Effect of triple therapy associated with initial periodontal therapy on the eradication of gastric Helicobacter pylori. Zhongguo Wuzhenxue Zazhi [Chinese Journal of Misdiagnostics] 2003;3(10):1485-6.	--- (article has not been accessed online) ---

Ricketts D, Lamont T, Innes NP, Kidd E, Clarkson JE http://doi.org/10.1002/14651858.CD003808.pub3

2013 Operative caries management in adults and children Primary studies: 8

2010 Bjørndal 2010 [doi.org/10.1111/j.1600-0722.2010.00731.x](doi.org/10.1111/j.1600-0722.2010.00731.x)	Bjørndal L, Reit C, Bruun G, Markvart M, Kjældgaard M, Näsman P, et al. Treatment of deep caries lesions in adults: randomized clinical trials comparing stepwise vs. direct complete excavation, and direct pulp capping vs. partial pulpotomy. European Journal of Oral Sciences 2010;118(3):290-7.	2 approved by Copenhagen and Frederiksberg ethics committees, Denmark & the Sahlgrenska Academy, University of Gothenburg, Sweden (#083-05)

2010 Orhan 2010
ingentaconnect.com/contentone/aapd/pd/2010/00000032/00000004/art00012
Orhan AI, Oz FT, Orhan K. Pulp exposure occurrence and outcomes after 1- or 2- visit indirect pulp therapy vs complete caries removal in primary and permanent molars. Pediatric Dentistry 2010;32(4):347-55.
1 approved by the Scientific Ethical Committee of Ankara University

2009 Lula 2009
doi.org/10.1159%2F000231572
Lula EC, Monteiro-Neto V, Alves CM, Ribeiro CC. Microbiological analysis after complete or partial removal of carious dentin in primary teeth: a randomized clinical trial. Caries Research 2009;43(5):354-8.
2 approved by the Ethics Committee of the University Hospital of the Federal University of Maranhão (HUUFMA, protocol 380/06

2007 Innes 2007
doi.org/10.1177%2F0022034511422064
Innes NP, Evans DJP, Stirrups DR. Sealing caries in primary molars: Randomized control trial, 5-year results. Journal of Dental Research 2011;90(12):1405-10.
2 approved by the Tayside Committee on Medical Research Ethics (approval 108/00)

1999 Ribeiro 1999
quintpub.com/journals/qi/fulltext.php?article_id=5378
Ribeiro CC, Baratieri LN, Perdigao J, Baratieri NM, Ritter AV. A clinical, radiographic, and scanning electron microscopic evaluation of adhesive restorations on carious dentin in primary teeth. Quintessence International 1999;30(9):591-9.
1 approved by the ethics committee of the federal university of Santa Catarina

1996 Leksell 1996
doi.org/10.1111/j.1600-9657.1996.tb00513.x
Leksell E, Ridell K, Cvek M, Mejare I. Pulp exposure after stepwise versus direct complete excavation of deep carious lesions in young posterior permanent teeth. Endodontics & Dental Traumatology 1996;12(4):192-6.
0 Ethics committee not mentioned

1987 Mertz-Fairhurst
doi.org/10.14219%2Fjada.archive.1987.0288
Mertz-Fairhurst EJ, Call-Smith KM, Shuster GS, Williams JE, Davis QB, Smith CD, et al. Clinical performance of sealed composite restorations placed over caries compared with sealed and unsealed amalgam restorations. Journal of the American Dental Association 1987;115(5):689-94.
--- (article has not been accessed online) ---

1977 Magnusson 197
ncbi.nlm.nih.gov/pubmed/282351
Magnusson BO, Sundell SO. Stepwise excavation of deep carious lesions in primary molars. Journal of the International Association of Dentistry for Children 1977;8(2):36-40.
--- (article has not been accessed online) ---

Riley P, Glenny AM, Worthington HV, Littlewood A, Clarkson JE, McCabe MG http://doi.org/10.1002/14651858.CD011552.pub2

2015 Interventions for preventing oral mucositis in patients with cancer receiving treatment: oral cryotherapy Primary studies: 14

2015 Askarifar 2015
doi.org/10.5812/ircmj.24775
Askarifar M, Lakdizaji S, Ramzi M, Rahmani A, Jabbarzadeh F. The effect of oral cryotherapy on chemotherapy-induced oral mucositis in patients undergoing autologous transplantation of blood stem cells: a clinical trial. Iranian Red Crescent Medical Journal 2015
1 approved by the regional research ethics committee of the Tabriz University of Medical Sciences.

Cochrane_Author		Primary studies_	doi
2013	Kakoei 2013	Kakoei S, Ghassemi A, Nakhaei NR. Effect of cryotherapy on oral mucositis in patients with head and neck cancers receiving radiotherapy. International Journal of Radiation Research 2013;11(2):117-20.	
search.proquest.com/docview/1419399937?accountid=1726 0		1 approved by the ethics committee and registered in the Iranian Registiy of Clinical Trials (IRCT)	
2013	Toro 2013	Toro JJ, Schneider D, Alonzo R, Hasan A, Lee S, Gushiken F, et al. A prospective, randomized clinical trial of cryotherapy vs. Supersaturated calcium phosphate rinses vs. Saline rinses for the prevention of oral mucositis in patients with multiple myeloma (MM) receiving high-dose melphalan (HDM) and autotransplantation. Biology of Blood and Marrow Transplantation 2014;20(2 Suppl):S204-5.	
doi.org/10.1016%2Fj.bbmt.2013.12.340		--- (article has not been accessed online) ---	
2012	Heydari 2012	Heydari A, Sharifi H, Salek R. Effect of oral cryotherapy on combination chemotherapy-induced oral mucositis: a randomized clinical trial. Middle East Journal of Cancer 2012;3(2/3):55-64.	
mejc.sums.ac.ir/index.php/mejc/article/view/42/33		1 approved by the Institutional Review Board	
2012	Katranci 2012	Katranci N, Ovayolu N, Ovayolu O, Sevinc A. Evaluation of the effect of cryotherapy in preventing oral mucositis associated with chemotherapy - a randomized controlled trial. European Journal of Oncology Nursing 2012;16(4):339-44.	
doi.org/10.1016%2Fj.ejon.2011.07.008		1 approved by the Ethics Committee of the Gaziantep University Medical Schoo	
2012	Salvador 2012	Salvador P, Azusano C, Wang L, Howell D. A pilot randomized controlled trial of an oral care intervention to reduce mucositis severity in stem cell transplant patients. Journal of Pain and Symptom Management 2012;44(1):64-73.	
doi.org/10.1016%2Fj.jpainsymman.2011.08.012		1 approved by the study site's Research Ethics Board	
2011	Zhang 2011	Zhang W, Li LS, Lu YW, Zhen JC, Zhang XL. Intervention research on preventing oral mucositis after using high dose methotrexate chemotherapy in osteosarcoma by gargling with calcium folinic. Chinese Pharmaceutical Journal 2011;46(14):1126-8.	
en.cnki.com.cn/Article_en/CJFDTotal-ZGYX201114027.htm		--- (article has not been accessed online) ---	
2008	Sorensen 2008	Sorensen JB, Skovsgaard T, Bork E, Damstrup L, Ingeberg S. Double-blind, placebo-controlled, randomized study of chlorhexidine prophylaxis for 5-fluorouracil-based chemotherapy-induced oral mucositis with nonblinded randomized comparison to oral cooling (cryotherapy) in gastrointestinal malignancies. Cancer 2008; Vol. 112, issue 7:1600-6.	
doi.org/10.1002/cncr.23328		1 approved by the local Ethical Committees.	
2007	Gori 2007	Gori E, Arpinati M, Bonifazi F, Errico A, Mega A, Alberani F, et al. Cryotherapy in the prevention of oral mucositis in patients receiving low-dose methotrexate following myeloablative allogeneic stem cell transplantation: a prospective randomized study of the Gruppo Italiano Trapianto di Midollo Osseo nurses group. Bone Marrow Transplantation 2007; Vol. 39, issue 6:347-52.	
doi.org/10.1038/sj.bmt.1705590		0 Ethics committee not mentioned	
2007	Svanberg 2007	Svanberg A, Birgegard G, Ohrn K. Oral cryotherapy reduces mucositis and opioid use after myeloablative therapy--a randomized controlled trial. Supportive Care in Cancer 2007; Vol. 15, issue 10:1155-61.	
doi.org/10.1007/s00520-007-0245-8		2 approved by the regional Research Ethics Committee, Uppsala, D.no; 02-137	

2006	Lilleby 2006 [doi.org/10.1038%2Fsj.bmt.1705384](doi.org/10.1038%2Fsj.bmt.1705384)	Lilleby K, Garcia P, Gooley T, McDonnell P, Taber R, Holmberg L, et al. A prospective, randomized study of cryotherapy during administration of high-dose melphalan to decrease the severity and duration of oral mucositis in patients with multiple myeloma undergoing autologous peripheral blood stem cell transplantation. Bone Marrow Transplantation 2006:37(11):1031-5.	1 approved by the FHCRC institutional review board
1994	Cascinu 1994 [doi.org/10.1016%2F0964-1955%2894%2990003-5](doi.org/10.1016%2F0964-1955%2894%2990003-5)	Cascinu S, Fedeli A, Fedeli SL, Catalano G. Oral cooling (cryotherapy), an effective treatment for the prevention of 5-fluorouracil-induced stomatitis. European Journal of Cancer. Part B, Oral Oncology 1994;30B(4):234-6.	0 Ethics committee not mentioned
1993	Rocke 1993 [doi.org/10.1002/1097-0142(19931001)72:7%3C2234::AID](doi.org/10.1002/1097-0142(19931001)72:7%3C2234::AID)	Rocke LK, Loprinzi CL, Lee JK, Kunselman SJ, Iverson RK, Finck G, et al. A randomized clinical trial of two different durations or oral cryotherapy for prevention of 5-fluorouracil-related stomatitis. Cancer 1993;72(7):2234-8.	0 Ethics committee not mentioned
1991	Mahood 1991 [doi.org/10.1200/JCO.1991.9.3.449](doi.org/10.1200/JCO.1991.9.3.449)	Mahood DJ, Dose AM, Loprinzi CL, Veeder MH, Athmann LM, Thereau TM, et al. Inhibition of fluorouracil-induced stomatitis by oral cryotherapy. Journal of Clinical Oncology 1991;9(3):449-52.	0 Ethics committee not mentioned

Riley P, Lamont T http://doi.org/10.1002/14651858.CD010514.pub2

2013 Triclosan/copolymer containing toothpastes for oral health Primary studies: 30

2012	Pradeep 2012 [doi.org/10.1902/jop.2011.110371](doi.org/10.1902/jop.2011.110371)	Pradeep AR, Agarwal E, Naik SB. Clinical and microbiologic effects of commercially available dentifrice containing aloe vera: a randomized controlled clinical trial. Journal of Periodontology 2012; Vol. 83, issue 6:797-804.	1 approved by the institutional ethical committee and review board of the Government Dental College and Research Institute, Bangalore, India
2011	Mankodi 2011 ncbi.nlm.nih.gov/pubmed/22216654	Mankodi S, Chaknis P, Panagakos FS, DeVizio W, Proskin HM. Comparative investigation of a dentifrice containing triclosan/copolymer/sodium fluoride and specially-designed silica and a dentifrice containing 0.243% sodium fluoride in a silica base for the control of established supra-gingival plaque and gingivitis: a 6-month clinical study. American Journal of Dentistry 2011; Vol. 24 Spec No A, issue Spec No A:21A-7A.	0 Ethics committee not mentioned
2009	Vered 2009 ncbi.nlm.nih.gov/pubmed/19591339	Vered Y, Zini A, Mann J, DeVizio W, Stewart B, Zhang YP, et al. Comparison of a dentifrice containing 0.243% sodium fluoride, 0.3% triclosan, and 2.0% copolymer in a silica base, and a dentifrice containing 0.243% sodium fluoride in a silica base: a three-year clinical trial of root caries and dental crowns among adults. Journal of Clinical Dentistry 2009; Vol. 20, issue 2:62-5.	--- (article has not been accessed online) ---
2008	Mateu 2008 https://www.ncbi.nlm.nih.gov/pubmed/19301514	Mateu FA, Boneta AE, DeVizio W, Stewart B, Proskin HM. A clinical investigation of the efficacy of two dentifrices for controlling established supragingival plaque and gingivitis. Journal of Clinical Dentistry 2008; Vol. 19, issue 3:85-94.	--- (article has not been accessed online) ---

Cochrane_Author		Primary studies_	doi
2006	Kraivaphan 200	Kraivaphan P, Amornchat C, Triratana T, Leethochawalit U. Clinical effect of a triclosan containing dentifrice on gingivitis during pregnancy and post-partum. Southeast Asian Journal of Tropical Medicine and Public Health 2006; Vol. 37, issue 4:820-5.	http://imsear.li.mahidol.ac.th/bitstream/123456789/32495/2/820.pdf
		0 Ethics committee not mentioned	
2006	Schiff 2006	Schiff T, Proskin HM, Zhang YP, Petrone M, DeVizio W. A clinical investigation of the efficacy of three different treatment regimens for the control of plaque and gingivitis. Journal of Clinical Dentistry 2006; Vol. 17, issue 5:138-44.	
		--- (article has not been accessed online) ---	
2002	Allen 2002	Allen DR, Battista GW, Petrone DM, Petrone ME, Chaknis P, DeVizio W, et al. The clinical efficacy of Colgate Total Plus Whitening Toothpaste containing a special grade of silica and Colgate Total Fresh Stripe Toothpaste in the control of plaque and gingivitis: a six-month clinical study. Journal of Clinical Dentistry 2002; Vol. 13, issue 2:59-64.	
		--- (article has not been accessed online) ---	
2002	Liu 2002	Liu H, Segreto VA, Baker RA, Vastola KA, Ramsey LL, Gerlach RW. Anticalculus efficacy and safety of a novel whitening dentifrice containing sodium hexametaphosphate: a controlled six-month clinical trial. Journal of Clinical Dentistry 2002; Vol. 13, issue 1:25-8.	
		--- (article has not been accessed online) ---	
2002	Triratana 2002	Triratana T, Rustogi KN, Volpe AR, DeVizio W, Petrone M, Giniger M. Clinical effect of a new liquid dentifrice containing triclosan/copolymer on existing plaque and gingivitis. Journal of the American Dental Association (1939) 2002; Vol. 133, issue 2:219-25.	doi.org/10.14219/jada.archive.2002.0147
		0 Ethics committee not mentioned	
2001	Mann 2001	Mann J, Vered Y, Babayof I, Sintes J, Petrone ME, Volpe AR, et al. The comparative anticaries efficacy of a dentifrice containing 0.3% triclosan and 2.0% copolymer in a 0.243% sodium fluoride/silica base and a dentifrice containing 0.243% sodium fluoride/silica base: a two-year coronal caries clinical trial on adults in Israel. Journal of Clinical Dentistry 2001; Vol. 12, issue 3:71-6.	
		--- (article has not been accessed online) ---	
1998	Ellwood 1998	Ellwood RP, Worthington HV, Blinkhorn AS, Volpe AR, Davies RM. Effect of a triclosan/copolymer dentifrice on the incidence of periodontal attachment loss in adolescents. Journal of Clinical Periodontology 1998; Vol. 25, issue 5:363-7.	doi.org/10.1111/j.1600-051X.1998.tb02456.x
		1 approved by the Central Manchester ResearchEthics Committee	
1997	Hu 1997	Hu D, Zhang J, Wan H, Zhang Y, Volpe AR, Petrone ME. Efficacy of a triclosan/copolymer dentifrice in the control of plaque and gingivitis: a six-month study in China. Hua Xi Kou Qiang Yi Xue Za Zhi [West China Journal of Stomatology] 1997;15(4):333-5.	ncbi.nlm.nih.gov/pubmed/11479983
		--- (article has not been accessed online) ---	
1997	McClanahan 19	McClanahan SF, Beiswanger BB, Bartizek RD, Lanzalaco AC, Bacca L, White DJ. A comparison of stabilized stannous fluoride dentifrice and triclosan/copolymer dentifrice for efficacy in the reduction of gingivitis and gingival bleeding: six-month clinical results. Journal of Clinical Dentistry 1997;8(2 Spec No):39-45.	ncbi.nlm.nih.gov/pubmed/9238872
		--- (article has not been accessed online) ---	

Cochrane_Author		Primary studies_	doi
1996	Feller 1996	Feller RP, Kiger RD, Triol CW, Sintes JL, Garcia L, Petrone ME, et al. Comparison of the clinical anticaries efficacy of an 1100 NaF silica-based dentifrice containing triclosan and a copolymer to an 1100 NaF silica-based dentifrice without those additional agents: a study on adults in California. Journal of Clinical Dentistry 1996;7(4):85-9.	ncbi.nlm.nih.gov/pubmed/9238883
		--- (article has not been accessed online) ---	
1996	Mann 1996	Mann J, Karniel C, Triol CW, Sintes JL, Garcia L, Petrone ME, et al. Comparison of the clinical anticaries efficacy of a 1500 NaF silica-based dentifrice containing triclosan and a copolymer to a 1500 NaF silica- based dentifrice without those additional agents: a study on adults in Israel. Journal of Clinical Dentistry 1996;7(4):90-5.	ncbi.nlm.nih.gov/pubmed/9238884
		--- (article has not been accessed online) ---	
1995	Hawley 1995	Hawley GM, Hamilton FA, Worthington HV, Davies RM, Holloway PJ, Davies TG, et al. A 30-month study investigating the effect of adding triclosan/copolymer to a fluoride dentifrice. Caries Research 1995; Vol. 29, issue 3:163-7.	
		--- (article has not been accessed online) ---	
1995	Kanchanakamol	Kanchanakamol U, Umpriwan R, Jotikasthira N, Srisilapanan P, Tuongratanaphan S, Sholitkul W, et al. Reduction of plaque formation and gingivitis by a dentifrice containing triclosan and copolymer. Journal of Periodontology 1995; Vol. 66, issue 2:109-12.	doi.org/10.1902/jop.1995.66.2.109
		0 Ethics committee not mentioned	
1995	Renvert 1995	Renvert S, Birkhed D. Comparison between 3 triclosan dentifrices on plaque, gingivitis and salivary microflora. Journal of Clinical Periodontology 1995; Vol. 22, issue 1:63-70.	doi.org/10.1111/j.1600-051X.1995.tb01772.x
		--- (article has not been accessed online) ---	
1994	Palomo 1994	Palomo F, Wantland L, Sanchez A, Volpe AR, McCool J, DeVizio W. The effect of three commercially available dentifrices containing triclosan on supragingival plaque formation and gingivitis: a six month clinical study. International Dental Journal 1994; Vol. 44, issue 1 Suppl 1:75-81.	
		--- (article has not been accessed online) ---	
1994	Triratana 1994	Triratana T, Amornchat C, Kraivaphan P, Tandhachoon K. Clinical study of a triclosan/copolymer dentifrice on plaque formation, gingivitis and streptococci mutans level in saliva. Journal of the Dental Association of Thailand 1994; Vol. 44, issue 1:27-31.	
		--- (article has not been accessed online) ---	
1993	Lindhe 1993	Lindhe J, Rosling B, Socransky SS, Volpe AR. The effect of a triclosan-containing dentifrice on established plaque and gingivitis. Journal of Clinical Periodontology 1993; Vol. 20, issue 5:327-34.	doi.org/10.1111/j.1600-051X.1993.tb00368.x
		--- (article has not been accessed online) ---	
1993	Svatun 1993	Svatun B, Sadxton CA, Huntington E, Cummins D. The effects of three silica dentifrices containing Triclosan on supragingival plaque and calculus formation and on gingivitis. International Dental Journal 1993; Vol. 43, issue 4 Suppl 1:441-52.	
		--- (article has not been accessed online) ---	

Cochrane_Author	Primary studies_	doi
1993	Triratana 1993	Triratana T, Tuongratanaphan S, Kraivaphan P, Rustogi KN, Volpe AR. The effect on established plaque formation and gingivitis of a Triclosan/copolymer/fluoride dentifrice: a six month clinical study. Journal of the Dental Association of Thailand 1993;43(1):19-28.
		--- (article has not been accessed online) ---
1992	Bolden 1992	Bolden TE, Zambon JJ, Sowinski J, Ayad F, McCool JJ, Volpe AR, et al. The clinical effect of a dentifrice containing triclosan and a copolymer in a sodium fluoride/silica base on plaque formation and gingivitis: a six-month clinical study. Journal of Clinical Dentistry 1992; Vol. 3, issue 4:125-31.
		--- (article has not been accessed online) ---
1992	Denepitiya 199	Denepitiya JL, Fine D, Singh S, DeVizio W, Volpe AR, Person P. Effect upon plaque formation and gingivitis of a triclosan/copolymer/fluoride dentifrice: a 6-month clinical study. American Journal of Dentistry 1992; Vol. 5, issue 6:307-11.
https://www.ncbi.nlm.nih.gov/pubmed/1304177		--- (article has not been accessed online) ---
1992	Mankodi 1992	Mankodi S, Walker C, Conforti N, DeVizio W, McCool JJ, Volpe AR. Clinical effect of a triclosan-containing dentifrice on plaque and gingivitis: a six-month study. Clinical Preventive Dentistry 1992; Vol. 14, issue 6:4-10.
		--- (article has not been accessed online) ---
1991	Cubells 1991	Cubells AB, Dalmau LB, Petrone ME, Chaknis P, Volpe AR. The effect of a triclosan/copolymer/fluoride dentifrice on plaque formation and gingivitis: a six-month clinical study. Journal of Clinical Dentistry 1991; Vol. 2, issue 3:63-9.
		--- (article has not been accessed online) ---
1991	Deasy 1991	Deasy MJ, Singh SM, Rustogi KN, Petrone DM, Battista G, Petrone ME, et al. Effect of a dentifrice containing triclosan and a copolymer on plaque formation and gingivitis. Clinical Preventive Dentistry 1991; Vol. 13, issue 6:12-9.
		--- (article has not been accessed online) ---
1991	Lobene 1991	Lobene RR, Battista GW, Petrone DM, Volpe AR, Petrone ME. Clinical efficacy of an anticalculus fluoride dentifrice containing triclosan and a copolymer: a 6-month study. American Journal of Dentistry 1991; Vol. 4, issue 2:83-5.
https://www.ncbi.nlm.nih.gov/pubmed/1854445		--- (article has not been accessed online) ---
1990	Garcia-Godoy 1	Garcia-Godoy F, DeVizio W, Volpe AR, Ferlauto RJ, Miller JM. Effect of a triclosan/copolymer/fluoride dentifrice on plaque formation and gingivitis: a 7-month clinical study. [Erratum appears in American Journal of Dentistry 1991;4(2):102]. American Journal of Dentistry 1990; Vol. 3 Spec No:S15-26.
https://www.ncbi.nlm.nih.gov/pubmed/2083041		--- (article has not been accessed online) ---

Riley P, Moore D, Ahmed F, Sharif MO, Worthington HV — http://doi.org/10.1002/14651858.CD010743.pub2

2015 Xylitol-containing products for preventing dental caries in children and adults — Primary studies: 10

Cochrane_Author	Primary studies_	doi
2014 Honkala 2014 doi.org/10.1159%2F000358399	Honkala S, Runnel R, Saag M, Olak J, Nõmmela R, Russak S, et al. Effect of erythritol and xylitol on dental caries prevention in children. Caries Research 2014;48(5):482-90.	2 approved by The Research Ethics Committee of the University of Tartu approved the study (166/T-7)
2013 Bader 2013 doi.org/10.14219%2Fjada.archive.2013.0010	Bader JD, Vollmer WM, Shugars DA, Gilbert GH, Amaechi BT, Brown JP, et al. Results from the Xylitol for Adult Caries Trial (X-ACT). Journal of the American Dental Association (1939) 2013;144(1):21-30.	1 approved by The institutional review board at each site
2013 Taipale 2013 doi.org/10.1159%2F000348424	Taipale T, Pienihäkkinen K, Alanen P, Jokela J, Söderling E. Administration of Bifidobacterium animalis subsp. lactis BB-12 in early childhood: a post-trial effect on caries occurrence at four years of age. Caries Research 2013;47(5):364-72.	1 approved by the Ethical Committee of the Hospital District of Southwest Finland.
2012 Lenkkeri 2012 doi.org/10.1111/j.1365-263X.2011.01182.x	Lenkkeri AM, Pienihäkkinen K, Hurme S, Alanen P. The caries-preventive effect of xylitol/maltitol and erythritol/maltitol lozenges: results of a double-blinded, cluster-randomized clinical trial in an area of natural fluoridation. International Journal of Paediatric Dentistry / the British Paedodontic Society [and] the International Association of Dentistry for Children 2012;22(3):180-90.	1 approved by the Ethics board of the Kotka Health Centre
2009 Milgrom 2009 doi.org/10.1001%2Farchpediatrics.2009.77	Milgrom P, Ly KA, Tut OK, Mancl L, Roberts MC, Briand K, et al. Xylitol pediatric topical oral syrup to prevent dental caries: a double-blind randomized clinical trial of efficacy. Archives of Pediatrics & Adolescent Medicine 2009;163(7):601-7.	1 approved by the University of Washington Institutional Review Board, Seattle
2006 Oscarson 2006 doi.org/10.1007%2FBF03262555	Oscarson P, Lif Holgerson P, Sjöström I, Twetman S, Stecksén-Blicks C. Influence of a low xylitol-dose on mutans streptococci colonisation and caries development in preschool children. European Archives of Paediatric Dentistry 2006;7(3):142-7.	1 approved by the local ethical committee of Umeå University.
2002 Sintes 2002 ncbi.nlm.nih.gov/pubmed/12572637	Sintes JL, Elias-Boneta A, Stewart B, Volpe AR, Lovett J. Anticaries efficacy of a sodium monofluorophosphate dentifrice containing xylitol in a dicalcium phosphate dihydrate base. A 30-month caries clinical study in Costa Rica. American Journal of Dentistry 2002;15(4):215-9.	--- (article has not been accessed online) ---
1995 Sintes 1995 ncbi.nlm.nih.gov/pubmed/8634157	Sintes JL, Escalante C, Stewart B, McCool JJ, Garcia L, Volpe AR, et al. Enhanced anticaries efficacy of a 0.243% sodium fluoride/10% xylitol/silica dentifrice: 3-year clinical results. American Journal of Dentistry 1995;8(5):231-5.	--- (article has not been accessed online) ---
1991 Petersson 1991 doi.org/10.1159%2F000261346	Petersson LG, Birkhed D, Gleerup A, Johansson M, Jönsson G. Caries-preventive effect of dentifrices containing various types and concentrations of fluorides and sugar alcohols. Caries Research 1991;25(1):74-9.	--- (article has not been accessed online) ---

Cochrane_Author Primary studies_ doi

Riley P, Worthington HV, Clarkson JE, Beirne PV http://doi.org/10.1002/14651858.CD004346.pub4

2013 Recall intervals for oral health in primary care patients Primary studies: 1

2012 Zhan 2012 Zhan L, Cheng J, Chang P, Ngo M, Denbesten PK, Hoover CI, et al. Effects of xylitol wipes on cariogenic bacteria and caries in young children. Journal of Dental Research 2012;91(7 Suppl):85S-90S.

doi.org/10.1177%2F0022034511434354

1 approved by The Committee on Human Research at the University of California, San Francisco (UCSF)

Rollason V, Laverriere A, MacDonald LC, Walsh T, Tramer MR, Vogt-Ferrier N http://doi.org/10.1002/14651858.CD008455.pub2

2016 Interventions for treating bisphosphonate-related osteonecrosis of the jaw (BRONJ) Primary studies: 1

2012 Freiberger 2012 Freiberger JJ, Padilla-Burgos R, McGraw T, Suliman HB, Kraft KH, Stolp BW, et al. What is the role of hyperbaric oxygen in the management of bisphosphonate-related osteonecrosis of the jaw: a randomized controlled trial of hyperbaric oxygen as an adjunct to surgery and antibiotics. Journal of Oral and Maxillofacial Surgery 2012;70:1573-83

doi.org/10.1016%2Fj.joms.2012.04.001

1 approved by the institutional review board

Schenkel AB, Peltz I, Veitz-Keenan A http://doi.org/10.1002/14651858.CD010526.pub2

2016 Dental cavity liners for Class I and Class II resin-based composite restorations Primary studies: 8

2013 Banom 2013 Banomyong D, Messer, H. Two-year clinical study on postoperative pulpal complications arising from the absence of a glass-ionomer lining in deep occlusal resin-composite restoration. Journal of Investigative and Clinical Dentistry 2013;4(4):265-70

doi.org/10.1111/j.2041-1626.2012.00160.x

2 approved by the ethics committee of Mahidol University, Thailand (ethics ID: MU 2007-109

2013 Strober 2013 Strober B, Veitz-Keenan A, Barna JA, Matthews AG, Vena D, Craig RG, et al. Effectiveness of a resin-modified glass ionomer liner in reducing hypersensitivity in posterior restorations: A study from the Practitioners Engaged in Applied Research and Learning Network. Journal of the American Dental Association 2013;144(8):886-97

doi.org/10.14219%2Fjada.archive.2013.0206

1 approved by the New York University School of Medicine institutional review board (IRB

2012 Boeckle 2012 Boeckler A, Schaller H-G, Gernhardt CR. A prospective, double-blind, randomized clinical trial of a one-step, self-etch adhesive with and without an intermediary layer of a flowable composite: a 2-year evaluation. Quintessence International 2012;43(4):279-86

quintpub.com/journals/qi/fulltext.php?article_id=12024

1 approved by the ethics committee of the university

2009 Burrow 2009 Burrow MF, Banomyong D, Harnirattisai C, Messer HH. Effect of glass-ionomer cement lining on postoperative sensitivity in occlusal cavities restored with resin composite - a randomized clinical trial. Operative Dentistry 2009;34(6):648-55.

doi.org/10.2341%2F08-098-C

2 approved by the Ethics in Human Research Committee of the University of Melbourne, Australia (# 0607777) and Mahidol University, Thailand (#: MU 2007-109)

2009 Wegehaupt 200 Wegehaupt F, Betke H, Solloch N, Musch U, Wiegand A, Attin T. Influence of cavity lining and remaining dentin thickness on the occurrence of postoperative hypersensitivity of composite restorations. Journal of Adhesive Dentistry 2009;11(2):137-41.

quintpub.com/journals/jad/fulltext.php?article_id=8384

2 approved by the ethics committe of university of Göttingen (# 5/11/02)

2006 Brownin 2006 Browning WD, Myers ML, Chan DC, Downey MC, Pohjola RM, Frazier KB. Performance of 2 packable composites at 12 months. Quintessence International 2006;37(5):361-8.

quintpub.com/journals/qi/fulltext.php?article_id=2225

1 approved by the campus institutional review board

2006 Efes 2006 Efes BG, Dorter C, Gomec Y, Koray F. Two-year clinical evaluation of ormocer and nanofill composite with and without a flowable liner. Journal of Adhesive Dentistry 2006;8(2):119-26

quintpub.com/journals/jad/fulltext.php?article_id=9122

1 approved by the ethics committee of the medical school of Istanbul university

2001 Akpata 2001 Akpata ES, Sadiq W. Post-operative sensitivity in glass-ionomer versus adhesive resin-lined posterior restorations. American Journal of Dentistry 2001;14(1):34-8

ncbi.nlm.nih.gov/pubmed/11806478

--- (article has not been accessed online) ---

Sequeira-Byron P, Fedorowicz Z, Carter B, Nasser M, Alrowaili EF http://doi.org/10.1002/14651858.CD009109.pub3

2015 Single crowns versus conventional fillings for the restoration of root-filled teeth Primary studies: 1

2002 Mannocci 2002 Mannocci F, Bertelli E, Sherriff M, Watson TF, Ford TR. Three-year clinical comparison of survival of endodontically treated teeth restored with either full cast coverage or with direct composite restoration. Journal of Prosthetic Dentistry 2002;88(3):297-301.

doi.org/10.1067%2Fmpr.2002.128492

1 approved by the appropriate committee of the University of Siena.

Sharif FN, Oliver R, Sweet C, Sharif MO http://doi.org/10.1002/14651858.CD008464.pub3

2015 Interventions for the treatment of keratocystic odontogenic tumours Primary studies: 0

EMPTY Empty Systematic Review

Sharif MO, Catleugh M, Merry A, Tickle M, Dunne SM, Brunton P, Aggarwal V http://doi.org/10.1002/14651858.CD005971.pub3

2014 Replacement versus repair of defective restorations in adults: resin composite Primary studies: 0

EMPTY Empty Systematic Review

Sharif MO, Merry A, Catleugh M, Tickle M, Brunton P, Dunne SM, Aggarwal V http://doi.org/10.1002/14651858.CD005970.pub3

2014 Replacement versus repair of defective restorations in adults: amalgam Primary studies: 0

Cochrane_Author | Primary studies_ | doi

EMPTY | Empty Systematic Review

Simpson TC, Weldon JC, Worthington HV, Needleman I, Wild SH, Moles DR, S | http://doi.org/10.1002/14651858.CD004714.pub3

2015 Treatment of periodontal disease for glycaemic control in people with diabetes mellitus | Primary studies: 35

2014	Gay 2014<br>doi.org/10.1111/jcpe.12268	Gay IC, Tran DT, Cavender AC, Weltman R, Chang J, Luckenbach E, et al. The effect of periodontal therapy on glycaemic control in a Hispanic population with type 2 diabetes: a randomized controlled trial. Journal of Clinical Periodontology 2014;41(7):673-80.	1 approved by the Committee for the Protection of Human Subjects
2014	Haerian Ardaka<br>jssu.ssu.ac.ir/article-1-2769-en.pdf	Haerian Ardakani A, Asadi Y, Afkhami Ardakani M, Nooorelahi M, Sooratgar A. Effect of topical tetracycline gel with non surgical periodontal therapy on Hba1c and lipid profile in type 2 diabetic patients: a clinico-biochemical study. Journal of Shahid Sadoughi University of Medical Sciences 2014;22(4):1387-95	--- (article has not been accessed online) ---
2014	Macedo 2014<br>doi.org/10.1007%2Fs10103-013-1285-6	Macedo G, Arthur B, Novaes J, Sergio L, Souza S, Mario T, et al. Additional effects of a PDT on nonsurgical periodontal treatment with doxycycline in type II diabetes: a randomized, controlled clinical trial. Lasers in Medical Science 2014;29:881-6	2 approved by the institution's Human Research Committee of School of Dentistry of São Paulo University (protocol,2006.1.934.58.2)
2014	Miranda 2014<br>doi.org/10.1111/jcpe.12282	Miranda TS, Feres M, Perez-Chaparro PJ, Faveri M, Figueiredo LC, Tamashiro NS, et al. Metronidazole and amoxicillin as adjuncts to scaling and root planing for the treatment of type 2 diabetic subjects with periodontitis: 1-year outcomes of a randomized placebo-controlled clinical trial. Journal of Clinical Periodontology 2014;41(9):890-9.	1 approved by the Guarulhos University Clinical Research Ethics Committee
2014	Raman 2014<br>doi.org/10.1186%2F1472-6831-14-79	Raman RP, Taiyeb-Ali TB, Chan SP, Chinna K, Vaithilingam RD. Effect of nonsurgical periodontal therapy versus oral hygiene instructions on Type 2 diabetes subjects with chronic periodontitis: a randomised clinical trial. BMC Oral Health 2014;14(1):2-19.	2 approved by the Medical Ethics Boards of University of Malaya Medical Centre (MEC Ref No: 696.9) and University of Malaya Dental Centre [DF PE1002/0045(P)
2014	Tsalikis 2014<br>doi.org/10.1111/jcpe.12287	Tsalikis L, Sakellari D, Dagalis P, Boura P, Konstantinidis A. Effects of doxycycline on clinical, microbiological and immunological parameters in well-controlled diabetes type-2 patients with periodontal disease: a randomized, controlled clinical trial. Journal of Clinical Periodontology 2014;41(10):972-80.	2 approved by the Ethical Committee of the School of Dentistry (#87458, Research Committee AUTH)
2013	Engebretson 20<br>doi.org/10.1016%2Fj.cct.2013.09.010	Engebretson S, Gelato M, Hyman L, Michalowicz BS, Schoenfeld E. Design features of the Diabetes and Periodontal Therapy Trial (DPTT): a multicenter randomized single-masked clinical trial testing the effect of nonsurgical periodontal therapy on glycosylated hemoglobin (HbA1c) levels in subjects with type 2 diabetes and chronic periodontitis. Contemporary Clinical Trials 2013;36(2):515-26.	1 approved by the institutional review boards at each participating center

Cochrane_Author	Primary studies_	doi
2013	Kothiwale 2013	Kothiwale SV, Kothiwale VA, Bhargava PV. Effect of non-invasive periodontal therapy on glycaemic control in type 2 diabetes mellitus patients - a randomized control trial. Diabetes 2013;62(Suppl 1):Abstract No A229.
		--- (article has not been accessed online) ---
2013	Pradeep 2013	Pradeep AR, Rao NS, Bajaj P, Kumari M. Efficacy of subgingivally delivered simvastatin in the treatment of patients with type 2 diabetes and chronic periodontitis: A randomized double-masked controlled clinical trial. Journal of Periodontology 2013;84(1):24-31.
doi.org/10.1902%2Fjop.2012.110721		1 approved by the Institutional Ethical Committee and Review Board of the Government Dental College and Research Institute
2013	Santos 2013	Santos VR, Lima JA, Miranda TS, Gonçalves TE, Figueiredo LC, Faveri M. Full-mouth disinfection as a therapeutic protocol for type-2 diabetic subjects with chronic periodontitis: Twelve-month clinical outcomes. A randomized controlled clinical trial. Journal of Clinical Periodontology 2013;40(2):155-62.
doi.org/10.1111/jcpe.12040		2 approved by the Guarulhos University's Ethics Committee in Clinical Research (# 100/2007).
2013	Zhang 2013	Zhang H, Li C, Shang S, Luo Z. Scaling and root planing with enhanced root planing on healthcare for type 2 diabetes mellitus: A randomized controlled clinical trial. Journal of Dental Sciences 2013;8(3):272-8
doi.org/10.1016%2Fj.jds.2012.10.009		2 approved by the ethics committee (School and Hospital of Stomatology, Wuhan University) with approval number 200712
2012	Chen 2012	Chen L, Luo G, Xuan D, Wei B, Liu F, Li J, et al. Effects of non-surgical periodontal treatment on clinical response, serum inflammatory parameters, and metabolic control in patients with type 2 diabetes: a randomized study. Journal of Periodontology 2012;83(4):435-43.
doi.org/10.1902%2Fjop.2011.110327		2 approved by the Medical Ethics Committee of Southern Medical University, Guangzhou, China, (ChiCTRTRC-10001062)
2012	Gilowski 2012	Gilowski L, Kondzielnik P, Wiench R, Plocica I, Strojek K, Krzeminski TF. Efficacy of short-term adjunctive subantimicrobial dose doxycycline in diabetic patients--randomized study. Oral Diseases 2012; Vol. 18, issue 8:763-70.
doi.org/10.1111/j.1601-0825.2012.01943.x.		1 approved by the Local Ethical Commit-tee (Institutional Review Board associated with theMedical University of Silesia, Katowice, Poland)
2012	Moeintaghavi 2	Moeintaghavi A, Arab HR, Bozorgnia Y, Kianoush K, Alizadeh M. Non-surgical periodontal therapy affects metabolic control in diabetics: a randomized controlled clinical trial. Australian Dental Journal 2012; Vol. 57, issue 1:31-7.
doi.org/10.1111/j.1834-7819.2011.01652.x		1 approved by the Research Council and the Ethical Committee of Mashhad University of Medical Sciences, Iran.
2012	NCT00801164	NCT00801164. Exploratory study of iocide oral rinse in a diabetic population (diabetes). clinicaltrials.gov/show/NCT00801164
		--- (article has not been accessed online) ---
2012	Santos 2012	Santos VR, Ribeiro FV, Lima JA, Miranda TS, Feres M, Bastos MF, et al. Partial- and full-mouth scaling and root planing in type 2 diabetic subjects: a 12-month follow-up of clinical parameters and levels of cytokines and osteoclastogenesis-related factors. Journal of Periodontal Research 2012;47(1):45-54.
doi.org/10.1111/j.1600-0765.2011.01403.x		1 approved by the Guarulhos University’s Ethics Committee in Clinical Research

Cochrane_Author		Primary studies_	doi
2011	Engebretson 20	Engebretson SP, Hey-Hadavi J. Sub-antimicrobial doxycycline for periodontitis reduces hemoglobin A1c in subjects with type 2 diabetes: a pilot study. Pharmacological Research 2011; Vol. 64, issue 6:624-9.	
doi.org/10.1016/j.phrs.2011.06.024		1 approved by the Committee on Human Research at Columbia University Medical Center	
2011	Koromantzos 2	Koromantzos PA, Makrilakis K, Dereka X, Katsilambros N, Vrotsos IA, Madianos PN. A randomized, controlled trial on the effect of non-surgical periodontal therapy in patients with type 2 diabetes. Part I: effect on periodontal status and glycaemic control. Journal of Clinical Periodontology 2011;38(2):142-7.	
doi.org/10.1111/j. 1600-051X. 2010.01652. x		1 approved by the University of Athens' Dental School and the participating hospital's ethical committees	
2011	Li 2011	Li Z, Sha YQ, Zhang BX, Zhu L, Kang J. [Effect of community periodontal care intervention on periodontal health and glycemic control in type 2 diabetic patients with chronic periodontitis]. [Chinese]. Beijing da Xue Xue Bao (Yi Xue Ban/Journal of Peking University. Health Sciences) 2011;43(2):285-9.	
		--- (article has not been accessed online) ---	
2011	Sun 2011	Sun WL, Chen LL, Zhang SZ, Wu YM, Ren YZ, Qin GM. Inflammatory cytokines, adiponectin, insulin resistance and metabolic control after periodontal intervention in patients with type 2 diabetes and chronic periodontitis. Internal Medicine 2011; Vol. 50, issue 15:1569-74	
doi.org/10.2169/internalmedicine.50.5166		1 approved by the Ethics Committee of the Second Hospital of Zhejiang University College of Medicine	
2009	Al-Zahrani 2009	Al-Zahrani MS, Bamshmous SO, Alhassani AA, Al-Sherbini MM. Short-term effects of photodynamic therapy on periodontal status and glycemic control of patients with diabetes. Journal of Periodontology 2009;80:1568-73.	
doi.org/10.1902%2Fjop.2009.090206		2 approved by the Deanship of Scientific Research at King Abdulaziz University (number 052/427)	
2009	Katagiri 2009	Katagiri S, Nitta H, Nagasawa T, Uchimura I, Izumiyama H, Inagaki K, et al. Multi-center intervention study on glycohemoglobin (HbA1c) and serum, high-sensitivity CRP (hs-CRP) after local anti-infectious periodontal treatment in type 2 diabetic patients with periodontal disease. Diabetes Research and Clinical Practice 2009;83(3):308-15.	
doi.org/10.1016%2Fj.diabres.2008.10.016		1 approved by the Ethics Committee for each university hospital	
2009	Santos 2009	Santos VR, Lima JA, De Mendonca AC, Maximo MBB, Faveri M, Duarte PM. Effectiveness of full-mouth and partial-mouth scaling and root planing in treating chronic periodontitis in subjects with type 2 diabetes. Journal of Periodontology 2009;80:1237-53.	
doi.org/10.1902%2Fjop.2009.090030		1 approved by the Guarulhos University's Ethics Committee in Clinical Research.	
2008	Llambés 2008	Llambés F, Silvestre FJ, Hernandez-Mijares A, Guiha R, Caffesse R. The effect of periodontal treatment on metabolic control of type 1 diabetes mellitus. Clinical Oral Investigations 2008;12(4):337-43.	
doi.org/10.1007%2Fs00784-008-0201-0		1 approved by the Research Committee of Dr. Peset Hospital	
2008	Madden 2008	Madden TE, Herriges B, Boyd LD, Laughlin G, Chiodo G, Rosenstein D. Alterations in HbA1c following minimal or enhanced non-surgical, non-antibiotic treatment of gingivitis or mild periodontitis in type 2 diabetic patients: a pilot trial. The Journal of Contemporary Dental Practice 2008;9(5):9-16.	
jaypeejournals.com/eJournals/ShowText.aspx?ID=1893		1 approved by the Oregon Health and Science University Institutional Review Board	

Cochrane_Author		Primary studies_	doi
2008	O'Connell 2008	O'Connell PA, Taba M, Nomizo A, Foss Freitas MC, Suaid FA, Uyemura SA, et al. Effects of periodontal therapy on glycemic control and inflammatory markers. Journal of Periodontology 2008;79(5):774-83.	doi.org/10.1902%2Fjop.2008.070250
		1 approved by the Institutional Human Research Committee, University of So Paulo-Ribeirao Preto.	
2008	Singh 2008	Singh S, Kumar V, Kumar S, Subbappa A. The effect of periodontal therapy on the improvement of glycaemic control in patients with type 2 diabetes mellitus: A randomized controlled clinical trial. International Journal of Diabetes in Developing Countries 2008;28(2):38-44.	doi.org/10.4103/0973-3930.43097
		1 approved by the colleage ethical committee for the use of human subjects in clinical experimentation	
2007	Jones 2007	Jones JA, Miller DR, Wehler CJ, Rich S, Krall E, Christiansen CL, et al. Study design, recruitment, and baseline characteristics: the Department of Veterans Affairs Dental Diabetes Study. Journal of Clinical Periodontology 2007;34(1):40-5.	doi.org/10.1111/j.1600-051X.2006.00998.x
		1 approved by The institutional review boards at each of the facilities	
2007	Yun 2007	Yun F, Firkova EI, Jun-Qi L, Xun H. Effect of non-surgical periodontal therapy on patients with type 2 diabetes mellitus. Folia Medica 2007;49(1-2):32-6.	https://www.ncbi.nlm.nih.gov/pubmed/18018467
		--- (article has not been accessed online) ---	
2005	Kiran 2005	Kiran M, Arpak N, Unsal E, Erdogan MF. The effect of improved periodontal health on metabolic control in type 2 diabetes mellitus. Journal of Clinical Periodontology 2005;32(3):266-72.	doi.org/10.1111/j.1600-051X.2005.00658.x
		1 approved by the Ethical Committee of Ankara University	
2004	Calbacho 2004	Calbacho V, Carrasco E, Wilckens M, Barboza P, Grant C, Aguirre M, et al. Evaluation of influence of conventional therapy in diabetics type 2. Journal of Dental Research 2004;84((Spec Iss B) Chilean section):65739.	
		2 approved by the Medical Ethics Committee of Southern MedicalUniversity, Guangzhou, China,(ChineseClinicalTrialRegistryNumberChiCTRTRC-10001062)	
2004	Skaleric 2004	Skaleric U, Schara R, Medvescek M, Hanlon A, Doherty F, Lessem J. Periodontal treatment by Arestin and its effects on glycemic control in type 1 diabetes patients. Journal of the International Academy of Periodontology 2004; Vol. 6:160-5.	https://www.ncbi.nlm.nih.gov/pubmed/15536785
		--- (article has not been accessed online) ---	
2003	Rodrigues 2003	Rodrigues DC, Taba MJ, Novaes AB, Souza SL, Grisi MF. Effect of non-surgical periodontal therapy on glycemic control in patients with type 2 diabetes mellitus. Journal of Periodontology 2003;74(9):1361-7.	doi.org/10.1902%2Fjop.2003.74.9.1361
		2 approved by the Medical Ethics Boards of University of Malaya Medical Centre (MEC Ref No: 696.9) and University of Malaya Dental Centre [DF PE1002/0045(P)	
2001	Rocha 2001	Rocha M, Nava LE, Vazquez de la Torre C, Sanchez-Marin F, Garay-Sevilla ME, Malacara JM. Clinical and radiological improvement of periodontal disease in patients with type 2 diabetes mellitus treated with alendronate: a randomized, placebo-controlled trial. Journal of Periodontology 2001;72(2):204-9.	doi.org/10.1902%2Fjop.2001.72.2.204
		2 approved by the Medical Ethics Boards of University of Malaya Medical Centre (MEC Ref No: 696.9) and University of Malaya Dental Centre [DF PE1002/0045(P)	

1997 Grossi 1997 doi.org/10.1902%2Fjop.1997.68.8.713	Grossi SG, Skrepinski FB, DeCaro T, Robertson DC, Ho AW, Dunford RG, et al. Treatment of periodontal disease in diabetics reduces glycated hemoglobin. Journal of Periodontology 1997;68(8):713-9.	0 Ethics committee not mentioned

Smail-Faugeron V, Courson F, Durieux P, Muller-Bolla M, Glenny AM, Fron Ch http://doi.org/10.1002/14651858.CD003220.pub2

2014 Pulp treatment for extensive decay in primary teeth Primary studies: 47

2011 Erdem 2011 ingentaconnect.com/content/aapd/pd/2011/00000033/[illegible]	Erdem AP, Guven Y, Balli B, Ilhan B, Sepet E, Ulukapi I, et al. Success rates of mineral trioxide aggregate, ferric sulfate, and formocresol pulpotomies: a 24-month study. Pediatric Dentistry 2011;33(2):165-70.	1 approved by the Ethics Committee of the Medical Faculty of Istanbul University, Istanbul, Turkey
2011 Malekafzali 201 ncbi.nlm.nih.gov/pubmed/22077689	Malekafzali B, Shekarchi F, Asgary S. Treatment outcomes of pulpotomy in primary molars using two endodontic biomaterials. A 2-year randomised clinical trial. European Journal of Paediatric Dentistry 2011;12(3):189-93	--- (article has not been accessed online) ---
2011 Pinky 2011 doi.org/10.4103%2F0970-4388.84684	Pinky C, Shashibhushan KK, Subbareddy VV. Endodontic treatment of necrosed primary teeth using two different combinations of antibacterial drugs: an in vivo study. Journal of the Indian Society of Pedodontics and Preventive Dentistry 2011;29(2):121-7.	0 Ethics committee not mentioned
2011 Subramaniam 2 doi.org/10.17796%2Fjcpd.35.4.1377v06621143233	Subramaniam P, Gilhotra K. Endoflas, zinc oxide eugenol and Metapex as root canal filling materials in primary molars - a comparative clinical study. Journal of Clinical Pediatric Ddentistry 2011;35(4):365-9	1 approved by the ethical committee of the institution
2010 Aminabadi 201 doi.org/10.17796%2Fjcpd.34.4.pntq604021604234	Aminabadi NA, Farahani RM, Oskouei SG. Formocresol versus calcium hydroxide direct pulp capping of human primary molars: two year follow-up. Journal of Clinical Pediatric Dentistry 2010;34(4):317-21	1 approved by the Ethics Committee and Research Council of the Tabriz University of Medical Sciences.
2010 Ansari 2010 doi.org/10.1111/j.1365-2591.2010.01695.x	Ansari G, Ranjpour M. Mineral trioxide aggregate and formocresol pulpotomy of primary teeth: a 2-year follow-up. International Endodontic Journal 2010;43(5):413-8	1 approved by the Shahid Beheshti University Medical Ethics Committe
2010 Doyle 2010 ingentaconnect.com/content/aapd/pd/2010/00000032/[illegible]	Doyle TL, Casas MJ, Kenny DJ, Judd PL. Mineral trioxide aggregate produces superior outcomes in vital primary molar pulpotomy. Pediatric Dentistry 2010;32(1):41-7	1 approved by the research ethics board of the hospital for sick children

Cochrane_Author		Primary studies_	doi
2010	Nakornchai 201	Nakornchai S, Banditsing P, Visetratana N. Clinical evaluation of 3Mix and Vitapex as treatment options for pulpally involved primary molars. International Journal of Paediatric Dentistry 2010;20(3):214-21.	doi.org/10.1111/j.1365-263X.2010.01044.x
		1 approved by the Committee on Human Rights Related to Human Experimentation, Mahidol University, Bangkok, Thailand	
2010	Ramar 2010	Ramar K, Mungara J. Clinical and radiographic evaluation of pulpectomies using three root canal filling materials: an in-vivo study. Journal of the Indian Society of Pedodontics and Preventive Dentistry 2010;28(1):25-9	doi.org/10.4103%2F0970-4388.60481
		0 Ethics committee not mentioned	
2010	Zealand 2010	Zealand CM, Briskie DM, Botero TM, Boynton JR, Hu JC. Comparing gray mineral trioxide aggregate and diluted formocresol in pulpotomized human primary molars. Pediatric Dentistry 2010;32(5):393-9	ingentaconnect.com/contentone/aapd/pd/2010/00000032/[illegible]
		1 approved by The Institutional Review Board at The University of Michigan, Ann Arbor, Michigan, and Mott Children’s Health Center (MCHC), Flint, Michigan	
2009	Alaçam 2009	Alaçam A, Odabas ME, Tuzuner T, Sillelioglu H, Baygin O. Clinical and radiographic outcomes of calcium hydroxide and formocresol pulpotomies performed by dental students. Oral Surgery, Oral Medicine, Oral Pathology, Oral Radiology & Endodontics 2009;108(5):e127-33.	doi.org/10.1016%2Fj.tripleo.2009.07.017
		0 Ethics committee not mentioned	
2009	Garrocho-Rang	Garrocho-Rangel A, Flores H, Silva-Herzog D, Hernandez-Sierra F, Mandeville P, Pozos-Guillen AJ. Efficacy of EMD versus calcium hydroxide in direct pulp capping of primary molars: a randomized controlled clinical trial. Oral Surgery, Oral Medicine, Oral Pathology, Oral Radiology & Endodontics 2009;107(5):733-8.	doi.org/10.1016%2Fj.tripleo.2008.12.017
		1 approved by the Ethics Committee on Investigation at the Faculty of Dentistry of San Luis Potosi University, Mexico	
2009	Sakai 2009	Sakai VT, Moretti ABS, Oliveira TM, Fornetti APC, Santos CF, Machado M, et al. Summary of pulpotomy of human primary molars with MTA and Portland cement: a randomised controlled trial. British Dental Journal 2009;207(3):128-9	doi.org/10.1038%2Fsj.bdj.2009.665
		2 approved by the Ethics Committee of the Bauru School of Dentistry, University of São Paulo (#34/2005 and #134/2004,	
2009	Subramaniam 2	Subramaniam P, Konde S, Mathew S, Sugnani S. Mineral trioxide aggregate as pulp capping agent for primary teeth pulpotomy: 2 year follow up study. Journal of Clinical Pediatric Dentistry 2009; Vol. 33, issue 4:311-4.	doi.org/10.17796/jcpd.33.4.r83r38423x58h38w
		1 approved by the institutional review board	
2008	Bahrololoomi 2	Bahrololoomi Z, Moeintaghavi A, Emtiazi M, Hosseini G. Clinical and radiographic comparison of primary molars after formocresol and electrosurgical pulpotomy: a randomized clinical trial. Indian Journal of Dental Research 2008; Vol. 19, issue 3:219-23.	ijdr.in/text.asp?2008/19/3/219/42954
		1 approved by the Statistics and Methodology Committee as well as by the Research Ethics Committee of Yazd University of Medical Sciences, Iran	
2008	Coser 2008	Coser RM, Gondim JO, Aparecida Giro EM. Evaluation of 2 endodontic techniques used to treat human primary molars with furcation radiolucency area: a 48-month radiographic study. Quintessence International 2008;39(7):549-57.	quintpub.com/journals/qi/fulltext.php?article_id=3440
		2 approved by the human subjects review Committee from the university center (NO 03/00)	

Cochrane_Author		doi	Primary studies_	
2008	Moretti 2008	doi.org/10.1111/j.1365-2591.2008.01377.x	Moretti AB, Sakai VT, Oliveira TM, Fornetti AP, Santos CF, Machado MA, et al. The effectiveness of mineral trioxide aggregate, calcium hydroxide and formocresol for pulpotomies in primary teeth. International Endodontic Journal 2008; Vol. 41, issue 7:547-55.	1 approved by The Ethics Committee of Bauru School of Dentistry,University of Sao Paulo
2008	Noorollahian 20	doi.org/10.1038/sj.bdj.2008.319	Noorollahian H. Comparison of mineral trioxide aggregate and formocresol as pulp medicaments for pulpotomies in primary molars. British Dental Journal 2008; Vol. 204, issue 11:E20.	1 approved by the Undersecretary for Research at Zahedan University of Medical Sciences
2008	Prabhakar 2008	jisppd.com/text.asp?2008/26/5/5/41746	Prabhakar AR, Sridevi E, Raju OS, Satish V. Endodontic treatment of primary teeth using combination of antibacterial drugs: an in vivo study. Journal of the Indian Society of Pedodontics and Preventive Dentistry 2008;26(5):5-10.	1 approved by ethical committee of concerned authorities
2008	Sabbarini 2008	doi.org/10.1016/j.joen.2007.12.002	Sabbarini J, Mohamed A, Wahba N, El-Meligy O, Dean J. Comparison of enamel matrix derivative versus formocresol as pulpotomy agents in the primary dentition. Journal of Endodontics 2008; Vol. 34, issue 3:284-7. Sakai 2009 {published data only}	0 Ethics committee not mentioned
2008	Sonmez 2008	doi.org/10.1016%2Fj.joen.2008.05.009	Sonmez D, Sari S, Cetinbas T. A comparison of four pulpotomy techniques in primary molars: a long-term follow-up. Journal of Endodontics 2008;34(8):950-5.	2 approved by the Ankara University, approval (no. 9-8/59-1791)
2008	Trairatvorakul 2	ingentaconnect.com/contentone/aapd/pd/2008/00000030/00000004/art00005	Trairatvorakul C, Chunlasikaiwan S. Success of pulpectomy with zinc oxide-eugenol vs calcium hydroxide/iodoform paste in primary molars: a clinical study. Pediatric Dentistry 2008; Vol. 30, issue 4:303-8.	1 approved by the Ethics Committee of Chulalongkorn University, Bangkok, Thailand
2008	Tuna 2008	doi.org/10.1111/j.1365-2591.2008.01377.x	Tuna D, Olmez A. Clinical long-term evaluation of MTA as a direct pulp capping material in primary teeth. International Endodontic Journal 2008; Vol. 41, issue 4:273	1 approved by The Ethics Committee of Bauru School of Dentistry,University of Sao Paulo
2008	Zurn 2008	ingentaconnect.com/contentone/aapd/pd/2008/00000030/00000001/art00008	Zurn D, Seale NS. Light-cured calcium hydroxide vs formocresol in human primary molar pulpotomies: a randomized controlled trial. Pediatric Dentistry 2008; Vol. 30, issue 1:34-41.	1 approved by the Institutional Review Board
2007	Aeinehchi 2007	doi.org/10.1111/j.1365-2591.2007.01209.x	Aeinehchi M, Dadvand S, Fayazi S, Bayat-Movahed S. Randomized controlled trial of mineral trioxide aggregate and formocresol for pulpotomy in primary molar teeth. International Endodontic Journal 2007; Vol. 40, issue 4:261-7.	1 approved by the Ethics Committee of Azad University of MedicalSciences

	Cochrane_Author		Primary studies_	doi
2007	Demir 2007	ncbi.nlm.nih.gov/pubmed/17672261	Demir T, Cehreli ZC. Clinical and radiographic evaluation of adhesive pulp capping in primary molars following hemostasis with 1.25% sodium hypochlorite: 2-year results. American Journal of Dentistry 2007;20(3):182-8.	1 approved by the Institutional Human Subject Review Committee
2006	Vargas 2006	ingentaconnect.com/contentone/aapd/pd/2006/00000028/00000006/art00006	Vargas KG, Packham B, Lowman D. Preliminary evaluation of sodium hypochlorite for pulpotomies in primary molars. Pediatric Dentistry 2006; Vol. 28, issue 6:511-7.	1 approved by the Internal Review Board of The University of Iowa, Iowa City, IA
2005	Farsi 2005	doi.org/10.17796%2Fjcpd.29.4.n80t77w625118k73	Farsi N, Alamoudi N, Balto K, Mushayt A. Success of mineral trioxide aggregate in pulpotomized primary molars. Journal of Clinical Pediatric Dentistry 2005;29(4):307-11.	0 Ethics committee not mentioned
2005	Holan 2005	ingentaconnect.com/contentone/aapd/pd/2005/00000027/00000002/art00007	Holan G, Eidelman E, Fuks AB. Long-term evaluation of pulpotomy in primary molars using mineral trioxide aggregate or formocresol. Pediatric Dentistry 2005; Vol. 27, issue 2:129-36	1 approved by the Institutional Review Board of Human Subjects experiments
2005	Huth 2005	doi.org/10.1177%2F154405910508401210	Huth KC, Paschos E, Hajek-Al-Khatar N, Hollweck R, Crispin A, Hickel R, et al. Effectiveness of 4 pulpotomy techniques - randomized controlled trial. Journal of Dental Research 2005;84(12):1144-8.	2 approved by the University of Munich, (No. 281/99)
2005	Markovic 2005		Markovic D, Zivojinovic V, Vucetic M. Evaluation of three pulpotomy medicaments in primary teeth. European Journal of Paediatric Dentistry 2005; Vol. 6, issue 3:133-8.	--- (article has not been accessed online) ---
2005	Naik 2005	doi.org/10.4103%2F0970-4388.16020	Naik S, Hegde AH. Mineral trioxide aggregate as a pulpotomy agent in primary molars: an in vivo study. Journal of the Indian Society of Pedodontics and Preventive Dentistry 2005;23(1):13-6.	1 approved by the institutional review board of human subjects experiments
2005	Ozalp 2005	https://www.ncbi.nlm.nih.gov/pubmed/16433405	Ozalp N, Saroglu I, Sonmez H. Evaluation of various root canal filling materials in primary molar pulpectomies: an in vivo study. American Journal of Dentistry 2005; Vol. 18, issue 6:347-50.	--- (article has not been accessed online) ---
2005	Saltzman 2005	doi.org/10.1111/j.1365-263X.2005.00670.x	Saltzman B, Sigal M, Clokie C, Rukavina J, Titley K, Kulkarni GV. Assessment of a novel alternative to conventional formocresol-zinc oxide eugenol pulpotomy for the treatment of pulpally involved human primary teeth: diode laser-mineral trioxide aggregate pulpotomy. International Journal of Paediatric Dentistry 2005; Vol. 15, issue 6:437-47.	1 approved by the University of Toronto Health Sciences Ethics Review Committee

Cochrane_Author		Primary studies_	doi	
2004	Agamy 2004	ingentaconnect.com/contentone/aapd/pd/2004/00000026/[illegible]	Agamy HA, Bakry NS, Mounir MM, Avery DR. Comparison of mineral trioxide aggregate and formocresol as pulp-capping agents in pulpotomized primary teeth. Pediatric Dentistry 2004; Vol. 26, issue 4:302-9.	0 Ethics committee not mentioned
2004	Casas 2004	ingentaconnect.com/contentone/aapd/pd/2004/00000026/[illegible]	Casas MJ, Kenny DJ, Johnston DH, Judd PL. Long-term outcomes of primary molar ferric sulfate pulpotomy and root canal therapy. Pediatric Dentistry 2004;26(1):44-8.	1 approved by the Research Ethics Board at The Hospital for Sick Children a
2004	Mortazavi 2004	doi.org/10.1111/j.1365-263X.2004.00544.x	Mortazavi M, Mesbahi M. Comparison of zinc oxide and eugenol, and Vitapex for root canal treatment of necrotic primary teeth. International Journal of Paediatric Dentistry 2004; Vol. 14, issue 6:417-24.	0 Ethics committee not mentioned
2002	Dean 2002	doi.org/10.1046/j.1365-263X.2002.00355.x	Dean JA, Mack RB, Fulkerson BT, Sanders BJ. Comparison of electrosurgical and formocresol pulpotomy procedures on children. International Journal of Paediatric Dentistry 2002;12:177-82	1 approved by the Indiana University Institutional Review Board
2001	Eidelman 2001	ncbi.nlm.nih.gov/pubmed/11242724	Eidelman E, Holan G, Fuks AB. Mineral trioxide aggregate vs. formocresol in pulpotomized primary molars: a preliminary report. Pediatric Dentistry 2001;23(1):15-8.	--- (article has not been accessed online) ---
2000	Ibricevic 2000	doi.org/10.17796%2Fjcpd.24.4.d7u6405nw1132705	Ibricevic H, Al-Jame Q. Ferric sulfate as pulpotomy agent in primary teeth: twenty month clinical follow-up. Journal of Clinical Pediatric Dentistry 2000;24(4):269-72	0 Ethics committee not mentioned
2000	Nadkarni 2000	http://medind.nic.in/jao/t00/i1/jaot00i1p1o.pdf	Nadkarni U, Damle SG. Comparative evaluation of calcium hydroxide and zinc oxide eugenol as root canal filling materials for primary molars: a clinical and radiographic study. Journal of the Indian Society of Pedodontics and Preventive Dentistry 2000;18(1):1-10	0 Ethics committee not mentioned
2000	Waterhouse 20	doi.org/10.1046/j.1365-263X.2002.00321.x	Waterhouse PJ, Nunn JH, Whitworth JM. Prostaglandin E2 and treatment outcome in pulp therapy of primary molars with carious exposures. International Journal of Paediatric Dentistry 2002;12(2):116-23	1 approved by the Combined Ethics Committee for Newcastle and North Tyneside Health Authorities and the University of Newcastle
1999	Shumayrikh 199	doi.org/10.1111/j.1600-9657.1999.tb00784.x	Shumayrikh NM, Adenubi JO. Clinical evaluation of glutaraldehyde with calcium hydroxide and glutaraldehyde with zinc oxide eugenol in pulpotomy of primary molars. Endodontics & Dental Traumatology 1999;15(6):259-64	1 approved by the Ethics Committee of the College of Dentistry Research Center (CDRC)

1997 Fuks 1997
Fuks AB, Holan G, Davis JM, Eidelman E. Ferric sulfate versus dilute formocresol in pulpotomized primary molars: long-term follow up. Pediatric Dentistry 1997;19(5):327-30.
ncbi.nlm.nih.gov/pubmed/9260226
--- (article has not been accessed online) ---

1996 Fishman 1996
Fishman SA, Udin RD, Good DL, Rodef F. Success of electrofulguration pulpotomies covered by zinc oxide and eugenol or calcium hydroxide: a clinical study. Pediatric Dentistry 1996;18(5):385-90
ncbi.nlm.nih.gov/pubmed/8897531
--- (article has not been accessed online) ---

1991 Fei 1991
Fei AL, Udin RD, Johnson R. A clinical study of ferric sulfate as a pulpotomy agent in primary teeth. Pediatric Dentistry 1991;13(6):327-32
ncbi.nlm.nih.gov/pubmed/1843987
--- (article has not been accessed online) ---

1989 Alaçam 1989
Alaçam A. Long term effects of primary teeth pulpotomies with formocresol, glutaraldehyde-calcium and glutaraldehyde-zinc oxide eugenol on succudaneous teeth. Journal of Pedodontics 1989;13:307-13.
--- (article has not been accessed online) ---

Sumanth KN, Prashanti E, Aggarwal H, Kumar P, Lingappa A, Muthu MS, Kiran
http://doi.org/10.1002/14651858.CD011930.pub2
2016 Interventions for treating post-extraction bleeding
Primary studies: 0

EMPTY
Empty Systematic Review

Taylor J, Glenny AM, Walsh T, Brocklehurst P, Riley P, Gorodkin R, Pemberton
http://doi.org/10.1002/14651858.CD011018.pub2
2014 Interventions for the management of oral ulcers in Behcet's disease
Primary studies: 15

2012 Fani 2012
Fani MM, Ebrahimi H, Pourshahidi S, Aflaki E, Shafiee Sarvestani S. Comparing the effect of phenytoin syrup and triamcinolone acetonide ointment on aphthous ulcers in patients with Behcet's syndrome. Iranian Red Crescent Medical Journal 2012;14(2):74-7.
ncbi.nlm.nih.gov/pmc/articles/PMC3372045/
0 Ethics committee not mentioned

2009 Kilic 2009
Kilic H, Zeytin HE, Korkmaz C, Mat C, Gul A, Cosan F, et al. Low-dose natural human interferon-alpha lozenges in the treatment of Behcet's syndrome. Rheumatology 2009;48(11):1388-91.
doi.org/10.1093%2Frheumatology%2Fkep237
1 approved by the local ethical committees of these medical schools, the Central Ethical Board of Ministry of Health of Turkey

2006 Mat 2006
Mat C, Yurdakul S, Uysal S, Gogus F, Ozyazgan Y, Uysal O, et al. A double-blind trial of depot corticosteroids in Behçet's syndrome. Rheumatology (Oxford, England) 2006;45(3):348-52.
doi.org/10.1093%2Frheumatology%2Fkei165
1 approved by the Ethics Committee of Cerrahpasa Medical Faculty

	Cochrane_Author	doi	Primary studies_	
2005	Melikoglu 2005	jrheum.org/content/32/1/98	Melikoglu M, Fresko I, Mat C, Ozyazgan Y, Gogus F, Yurdakul S, et al. Short-term trial of etanercept in Behcet's disease: a double blind, placebo controlled study. Journal of Rheumatology 2005;32(1):98-105.	1 approved by the faculty ethics committee
2003	Matsuda 2003	doi.org/10.2165%2F00126839-200304010-00002	Matsuda T, Ohno S, Hirohata S, Miyanaga Y, Ujihara H, Inaba G, et al. Efficacy of rebamipide as adjunctive therapy in the treatment of recurrent oral aphthous ulcers in patients with Behcet's disease: A randomised, double-blind, placebo-controlled study. Drugs in R&D 2003;4(1):19-28.	--- (article has not been accessed online) ---
2002	Alpsoy 2002	doi.org/10.1001%2Farchderm.138.4.467	Alpsoy E, Durusoy C, Yilmaz E, Ozgurel Y, Ermis O, Yazar S, et al. Interferon alfa-2a in the treatment of Behcet disease: a randomized placebo-controlled and double-blind study. Archives of Dermatology 2002;138(4):467-71.	1 approved by the ethics committees
2001	Yurdakul 2001	doi.org/10.1002/1529-0131(200111)44:11%3C2686::[illegible]	Yurdakul S, Mat C, Tüzün Y, Ozyazgan Y, Hamuryudan V, Uysal O, et al. A double-blind trial of colchicine in Behçet's syndrome. Arthritis and Rheumatism 2001;44(11):2686-92.	0 Ethics committee not mentioned
1999	Alpsoy 1999	doi.org/10.1001%2Farchderm.135.5.529	Alpsoy E, Er H, Durusoy C, Yilmaz E. The use of sucralfate suspension in the treatment of oral and genital ulceration of Behcet disease: a randomized, placebo-controlled, double-blind study. Archives of Dermatology 1999;135(5):529-32.	1 approved by the ethics committees
1998	Hamuryudan 19	doi.org/10.7326%2F0003-4819-128-6-199803150-00004	Hamuryudan V, Mat C, Saip S, Ozyazgan Y, Siva A, Yurdakul S, et al. Thalidomide in the treatment of the mucocutaneous lesions of the Behcet syndrome. A randomized, double-blind, placebo-controlled trial. Annals of Internal Medicine 1998;6:443-50.	1 approved by the institutional ethics committee of the Cerrahpasa Medical Faculty
1997	Ergun 1997	doi.org/10.1111/j.1365-4362.1997.tb03133.x	Ergun T, Gurbuz O, Yurdakul S, Hamuryudan V, Bekiroglu N, Yazici H, et al. Topical cyclosporine-A for treatment of oral ulcers of Behcet's syndrome [letter]. International Journal of Dermatology 1997;36(9):720.	--- (article has not been accessed online) ---
1992	Koc 1992	doi.org/10.3109%2F09546639209088723	Koc Y, Akpek G, Akpolat T, Gullu I, Kansu E, Kiraz S, et al. Topical sucralfate therapy for oral ulcers in Behcet's disease: a randomized double-blind study. Journal of Dermatological Treatment 1992;3(4):197-9.	--- (article has not been accessed online) ---
1991	Hamuryudan 19	doi.org/10.1093%2Frheumatology%2F30.5.395-a	Hamuryudan V, Yurdakul S, Rosenkaimer F, Yazici H. Inefficacy of topical alpha interferon in the treatment of oral ulcers of Behcet's syndrome: a randomized, double blind trial. British Journal of Rheumatology 1991;30(5):395-6.	--- (article has not been accessed online) ---

1989	Masuda 1989	Masuda K, Nakajima A, Urayama A, Nakae K, Kogure M, Inaba G. Double-masked trial of cyclosporin versus colchicine and long-term open study of cyclosporin in Behcet's disease. Lancet 1989;1(8647):1093-6.	
doi.org/10.1016%2FS0140-6736%2889%2992381-7		0 Ethics committee not mentioned	
1988	Davies 1988	Davies UM, Palmer RG, Denman AM. Treatment with acyclovir does not affect orogenital ulcers in Behcet's syndrome: a randomized double-blind trial. British Journal of Rheumatology 1988;27(4):300-2.	
doi.org/10.1093%2Frheumatology%2F27.4.300		--- (article has not been accessed online) ---	
1980	Aktulga 1980	Aktulga E, Altaç M, Müftüoglu A, Ozyazgan Y, Pazarli H, Tüzün Y, et al. A double blind study of colchicine in Behçet's disease. Haematologica 1980;65(3):399-402.	
		--- (article has not been accessed online) ---	

Thiruvenkatachari B, Harrison JE, Worthington HV, O'Brien KD http://doi.org/10.1002/14651858.CD003452.pub3

2013 Orthodontic treatment for prominent upper front teeth (Class II malocclusion) in children Primary studies: 17

2012	Yaqoob 2012	Yaqoob O, Dibiase AT, Fleming PS, Cobourne MT. Use of the Clark Twin Block functional appliance with and without an upper labial bow: a randomized controlled trial. The Angle Orthodontist 2012;82(2):363-9.	
doi.org/10.2319%2F041411-268.1		2 approved by the East Kent Research Ethics Committee (08/H1103/66)	
2011	Bilgiç 2011	Bilgiç F, Hamamci O, Başaran G. Comparison of the effects of fixed and removable functional appliances on the skeletal and dentoalveolar structures. Australian Orthodontic Journal 2011;27(2):110-6.	
ncbi.nlm.nih.gov/pubmed/22372266		1 approved by the Human Ethics Committee at the University of Otago	
2011	Jamilian 2011	Jamilian A, Showkatbakhsh R, Amiri SS. Treatment effects of the R-appliance and twin block in Class II division 1 malocclusion. European Journal of Orthodontics 2011;33(4):354-8.	
doi.org/10.1093%2Fejo%2Fcjq082		0 Ethics committee not mentioned	
2011	Showkatbakhsh	Showkatbakhsh R, Meybodi SE, Jamilian A, Meybodi SA, Meybodi EM. Treatment effects of R-appliance and anterior inclined bite plate in class II, division I malocclusion. Journal of Applied Oral Science 2011;19(6):634-8.	
doi.org/10.1590%2FS1678-77572011000600015		0 Ethics committee not mentioned	
2010	Thiruvenkatach	Thiruvenkatachari B, Sandler J, Murray A, Walsh T, O'Brien K. Comparison of Twin-block and Dynamax appliances for the treatment of Class II malocclusion in adolescents: a randomized controlled trial. American Journal of Orthodontics and Dentofacial Orthopedics 2010;138(2):144.e1-9; discussion 144-5.	
doi.org/10.1016%2Fj.ajodo.2010.01.025		1 approved by the Central Research Ethics Committee of the United Kingdom in 2007	

2009	UK (Mixed) 200	O'Brien K, Wright J, Conboy F, Appelbe P, Davies L, Connolly I, et al. Early treatment for Class II Division 1 malocclusion with the Twin-block appliance: a multi-center, randomized, controlled trial. American Journal of Orthodontics and Dentofacial Orthopedics 2009;135(5):573-9.	
doi.org/10.1016%2Fj.ajodo.2007.10.042			0 Ethics committee not mentioned
2007	Lee 2007	Lee RT, Kyi CS, Mack GJ. A controlled clinical trial of the effects of the Twin Block and Dynamax appliances on the hard and soft tissues. European Journal of Orthodontics 2007;29(3):272-82.	
doi.org/10.1093%2Fejo%2Fcjm004			1 approved by the East London and City Local Research Committee
2004	Banks 2004	Banks P, Wright J, O'Brien K. Incremental versus maximum bite advancement during twin-block therapy: a randomized controlled clinical trial. American Journal of Orthodontics and Dentofacial Orthopedics 2004;126(5):583-8.	
doi.org/10.1016%2Fj.ajodo.2004.03.024			1 approved by the local research ethics committees
2004	North Carolina	Tulloch JF, Proffit WR, Phillips C. Outcomes in a 2-phase randomized clinical trial of early Class II treatment. American Journal of Orthodontics and Dentofacial Orthopedics 2004;125(6):657-67.	
doi.org/10.1016%2Fj.ajodo.2004.02.008			0 Ethics committee not mentioned
2003	Cevidanes 2003	Cevidanes LH, Franco AA, Scanavini MA, Vigorito JW, Enlow DH, Proffit WR. Clinical outcomes of Frankel appliance therapy assessed with a counterpart analysis. American Journal of Orthodontics and Dentofacial Orthopedics 2003;123(4):379-87.	
doi.org/10.1067%2Fmod.2003.74			0 Ethics committee not mentioned
2003	UK (11-14) 2003	O'Brien K, Wright J, Conboy F, Sanjie Y, Mandall N, Chadwick S, et al. Effectiveness of treatment for Class II malocclusion with the Herbst or twin-block appliances: a randomized, controlled trial. American Journal of Orthodontics and Dentofacial Orthopedics 2003;124(2):128-37.	
doi.org/10.1016%2FS0889-5406%2803%2900345-7			1 approved by the relevant ethics committees
2000	New Zealand 20	Courtney M, Harkness M, Herbison P. Maxillary and cranial base changes during treatment with functional appliances. American Journal of Orthodontics and Dentofacial Orthopedics 1996;109(6):616-24.	
doi.org/10.1016%2FS0889-5406%2896%2970073-2			0 Ethics committee not mentioned
1998	Florida 1998	Chen DR, McGorray SP, Dolce C, Wheeler TT. Effect of early Class II treatment on the incidence of incisor trauma. American Journal of Orthodontics and Dentofacial Orthopedics 2011;140(4):e155-60.	
doi.org/10.1016%2Fj.ajodo.2011.02.023			0 Ethics committee not mentioned
1998	Ghafari 1998	Ghafari J, Shofer FS, Jacobsson-Hunt U, Markowitz DL, Laster LL. Headgear versus function regulator in the early treatment of Class II, division 1 malocclusion: a randomized clinical trial. American Journal of Orthodontics and Dentofacial Orthopedics 1998;113(1):51-61	
doi.org/10.1016%2FS0889-5406%2898%2970276-8			0 Ethics committee not mentioned

1998	London 1998	Illing HM, Morris DO, Lee RT. A prospective evaluation of Bass, Bionator and Twin Block appliances. Part I--The hard tissues. European Journal of Orthodontics 1998;20(5):501-16.
doi.org/10.1093%2Fejo%2F20.5.501		0 Ethics committee not mentioned
1997	Cura 1997	Cura N, Sarac M. The effect of treatment with the Bass appliance on skeletal Class II malocclusions: a cephalometric investigation. European Journal of Orthodontics 1997;19(6):691-702.
doi.org/10.1093%2Fejo%2F19.6.691		0 Ethics committee not mentioned
1997	Mao 1997	Mao J, Zhao H. The correction of Class II, division 1 malocclusion with bionator headgear combination appliance. Journal of Tongji Medical University 1997;17(4):254-6.
doi.org/10.1007%2FBF02895633		0 Ethics committee not mentioned

Walsh T, Liu JL, Brocklehurst P, Glenny AM, Lingen M, Kerr AR, Ogden G, War http://doi.org/10.1002/14651858.CD010173.pub2

2013 Clinical assessment to screen for the detection of oral cavity cancer and potentially malignant disorders in apparently h Primary studies: 13

	DIAGNOSTIC TE	REVIEW OF DIAGNOSTIC TESTS

Walsh T, Oliveira-Neto JM, Moore D http://doi.org/10.1002/14651858.CD008457.pub2

2015 Chlorhexidine treatment for the prevention of dental caries in children and adolescents Primary studies: 8

2013	Plonka 2013	Plonka KA, Pukallus ML, Holcombe TF, Barnett AG, Walsh LJ, Seow WK. Randomized controlled trial: a randomized controlled clinical trial comparing a remineralizing paste with an antibacterial gel to prevent early childhood caries. Pediatric Dentistry 2013;35(1):8E-12.
ingentaconnect.com/contentone/aapd/pd/2013/00000035/00000001/art00016		1 approved by the University of Queensland and Queensland Health
2013	Pukallus 2013	Pukallus ML, Plonka KA, Barnett AG, Walsh LJ, Holcombe TF, Seow WK. A randomised, controlled clinical trial comparing chlorhexidine gel and low-dose fluoride toothpaste to prevent early childhood caries. International Journal of Paediatric Dentistry 2013;23:216-24.
doi.org/10.1111/j.1365-263X.2012.01248.x		1 approved by the Queensland Health and The University of Queensland
2006	Du 2006	Du MQ, Tai BJ, Jiang H, Lo EC, Fan MW, Bian Z. A two-year randomized clinical trial of chlorhexidine varnish on dental caries in Chinese preschool children. Journal of Dental Research 2006;85(6):557-9.
doi.org/10.1177%2F154405910608500615		1 approved by the Ethics Committee of the School of Dentistry of the Wuhan University
2003	Baca 2003	Baca P, Junco P, Bravo M, Baca AP, Munoz MJ. Caries incidence in permanent first molars after discontinuation of a school-based chlorhexidine-thymol varnish program. Community Dentistry and Oral Epidemiology 2003;31(3):179-83.
doi.org/10.1034/j.1600-0528.2003.00034.x		0 Ethics committee not mentioned

Cochrane_Author	Primary studies_	doi

2002	De Soet 2002	De Soet JJ, Gruythuysen RJ, Bosch JA, Van Amerongen WE. The effect of 6-monthly application of 40% chlorhexidine varnish on the microflora and dental caries incidence in a population of children in Surinam. Caries Research 2002;36(6):449-55.
doi.org/10.1159%2F000066536		0 Ethics committee not mentioned
2000	Forgie 2000	Forgie AH, Paterson M, Pine CM, Pitts NB, Nugent ZJ. A randomised controlled trial of the caries-preventive efficacy of a chlorhexidine-containing varnish in high-caries-risk adolescents. Caries Research 2000;34(5):432-9.
doi.org/10.1159%2F000016619		1 approved by the Tayside Committee on Medical Research Ethics and Education Authorities
1999	Nordling 1999	Nordling M, Koch G, Hallonsten AL. Effect of a chlorhexidine varnish regiment on caries in children. International Journal of Paediatric Dentistry. 1999; Vol. 9:(Suppl 1):20 (Abstr 14.03) 17th Congress of the IAPD, London 2-4 Sept 1999.
		--- (article has not been accessed online) ---
1997	Bretz 1997	Bretz WA, do Valle EV, Almeida R, Djahjah C, Chen YM, Schork MA. Effects of a chlorhexidine varnish on the mutans streptococci and on dental caries. Biofilm Journal 1997;2(1):BF97002.
		--- (article has not been accessed online) ---

Wang Y, Li C, Yuan H, Wong MC, Zou J, Shi Z, Zhou X — http://doi.org/10.1002/14651858.CD009858.pub2

2016 Rubber dam isolation for restorative treatment in dental patients — Primary studies: 4

2013	Ammann 2013	Ammann P, Kolb A, Lussi A, Seemann R. Influence of rubber dam on objective and subjective parameters of stress during dental treatment of children and adolescents - a randomized controlled clinical pilot study. International Journal of Paediatric Dentistry 2013;23(2):110-5.
doi.org/10.1111/j.1365-263X.2012.01232.x		2 approved by the ethics committee of the University Clinic Charité (Berlin, Germany, (#EA2/081/08)
2012	Ma 2012	Ma J. Influence of rubber dam isolation on the performance of restorations for teeth wedge-shaped defects. Chinese Community Doctors 2012;14(309):164.
		--- (article has not been accessed online) ---
2010	Carvalho 2010	Carvalho TS, Sampaio FC, Diniz A, Bönecker M, Van Amerongen WE. Two years survival rate of Class II ART restorations in primary molars using two ways to avoid saliva contamination. International Journal of Paediatric Dentistry 2010;20(6):419-25.
doi.org/10.1111/j.1365-263X.2010.01060.x		2 approved by the Committee of Ethics in Research from the Federal University of Paraíba, under protocol number 134/04.
2010	Kemoli 2010	Kemoli AM, van Amerongen WE, Opinya GN. Short communication: Influence of different isolation methods on the survival of proximal ART restorations in primary molars after two years. European Archives of Paediatric Dentistry 2010;11(3):136-9.
doi.org/10.1007%2FBF03262729		1 approved by the University of Nairobi and the Kenyatta National Hospital Research and Ethical Committees

Watkinson S, Harrison JE, Furness S, Worthington HV — http://doi.org/10.1002/14651858.CD003451.pub2

2013 Orthodontic treatment for prominent lower front teeth (Class III malocclusion) in children — Primary studies: 7

Cochrane_Author Primary studies_ doi

2010 Abdelnaby 201 Abdelnaby YL, Nassar EA. Chin cup effects using two different force magnitudes in the management of Class III malocclusions. Angle Orthodontist 2010;80(5):957-62.

doi.org/10.2319%2F022210-110.1

0 Ethics committee not mentioned

2010 Atalay 2010 Atalay Z, Tortop T. Dentofacial effects of a modified tandem traction bow appliance. European Journal of Orthodontics 2010;32(6):655-61.

doi.org/10.1093%2Fejo%2Fcjp153

1 approved by the Health Science Institute of Gazi University

2010 Mandall 2010 Mandall N, Cousley R, DiBiase A, Dyer F, Littlewood S, Mattick R, et al. Is early class III protraction facemask treatment effective? A multicentre, randomized, controlled trial: 15-month follow-up. Journal of Orthodontics 2010;37(3):149-61.

doi.org/10.1179%2F14653121043056

2 approved by the multicentre and local ethical committees(MREC reference: 03/8/2)

2005 Vaughn 2005 Vaughn GA, Mason B, Moon HB, Turley PK. The effects of maxillary protraction therapy with or without rapid palatal expansion: A prospective, randomized clinical trial. American Journal of Orthodontics and Dentofacial Orthopedics 2005;128(3):299-309.

doi.org/10.1016%2Fj.ajodo.2005.04.030

0 Ethics committee not mentioned

2002 Keles 2002 Keles A, Tokmak EC, Erverdi N, Nanda R. Effect of varying the force direction on maxillary orthopedic protraction. Angle Orthodontist 2002;72(5):387–96.

angle.org/doi/10.1043/0003-3219(2002)072<0387:EOVTFD [illegible]

0 Ethics committee not mentioned

2001 Xu 2001 Xu B, Lin J. The orthopedic treatment of skeletal class III malocclusion with maxillary protraction therapy. Chinese Journal of Stomatology 2001;36(6):401-3.

cmadent.com/CN112144200106/654999.htm

--- (article has not been accessed online) ---

1994 Arun 1994 Arun T, Erverdi N. A cephalometric comparison of mandibular headgear and chin-cap appliances in orthodontic and orthopaedic view points. Journal of Marmara University Dental Faculty 1994;2(1):392-8.

--- (article has not been accessed online) ---

Worthington HV, Clarkson JE, Bryan G, Beirne PV http://doi.org/10.1002/14651858.CD004625.pub4

2013 Routine scale and polish for periodontal health in adults Primary studies: 3

2011 Jones 2011 Jones CL, Milsom KM, Ratcliffe P, Wyllie A, Macfarlane TV, Tickle M. Clinical outcomes of single-visit oral prophylaxis: a practice-based randomised controlled trial. BMC Oral Health 2011;11:35.

doi.org/10.1186%2F1472-6831-11-35

2 approved by the Cheshire Local Research Ethics Committee (reference: Q/1506/100.)

1985 Listgarten 1985 Listgarten MA, Schifter CC, Laster L. 3-year longitudinal study of the periodontal status of an adult population with gingivitis. Journal of Clinical Periodontology 1985;12(13):225-38.

doi.org/10.1111/j.1600-051X.1985.tb00920.x

--- (article has not been accessed online) ---

1971 Lightner 1971 Lightner LM, O'Leary JT, Drake RB, Crump PP, Allen MF. Preventive periodontic treatment procedures: results over 46 months. Journal of Periodontology 1971;42(9):555-61.

doi.org/10.1902%2Fjop.1971.42.9.555

0 Ethics committee not mentioned

Yeung CA, Chong LY, Glenny AM http://doi.org/10.1002/14651858.CD003876.pub4

2015 Fluoridated milk for preventing dental caries Primary studies: 1

2004 Maslak 2004 Maslak EE, Afonina IV, Kchmizova TG, Litovkina LS, Luneva NA. The effect of a milk fluoridation project in Volgograd. Caries Research 2004;38(4):377. Abstract no. 60.

--- (article has not been accessed online) ---

Yu Y, Sun J, Lai W, Wu T, Koshy S, Shi Z http://doi.org/10.1002/14651858.CD008734.pub2

2013 Interventions for managing relapse of the lower front teeth after orthodontic treatment Primary studies: 0

EMPTY Empty Systematic Review

Yaacob M, Worthington HV, Deacon SA, Deery C, Walmsley AD, Robinson PG, http://doi.org/10.1002/14651858.CD002281.pub3

2014 Powered versus manual toothbrushing for oral health Primary studies: 56

2011 Kallar 2011 Kallar S, Pandit IK, Srivastava N, Gugnani N. Plaque removal efficacy of powered and manual toothbrushes under supervised and unsupervised conditions: A retrospective clinical study. Journal of Indian Society of Pedodontics and Preventive Dentistry 2011;29(3):235-8.

doi.org/10.4103%2F0970-4388.85832

0 Ethics committee not mentioned

2010 Biavati Silvestri Silvestrini Biavati A, Gastaldo L, Dessi M, Biavati Silvestrini F, Migliorati M. Manual Orthodontic versus oscillating-rotating electric toothbrush in orthodontic patients: a randomised clinical trial. European Journal of Pediatric Dentistry 2010;11:200-2.

ejpd.eu/bibliografia_dettaglio.asp?id=353

--- (article has not been accessed online) ---

2010 Sharma 2010 Sharma NC, Qaqish JG, He T, Walters PA, Grender JM, Biesbrock AR. Plaque and gingivitis reduction efficacy of an advanced pulsonic toothbrush: a 4-week randomized and controlled clinical trial. American Journal of Dentistry 2010;23(6):305-10.

ncbi.nlm.nih.gov/pubmed/21344827

--- (article has not been accessed online) ---

	Cochrane_Author	doi	Primary studies_	
2009	Dorfer 2009	jaypeejournals.com/eJournals/ShowText.aspx?ID=1976	Dorfer CE, Joerss D, Wolff D. A prospective clinical study to evaluate the effect of manual and power toothbrushes on pre-existing gingival recessions. Journal of Contemporary Dental Practice 2009;10(4):1-8.	1 approved by the Independent International Freiburg Ethics Committee
2009	McCracken 200	doi.org/10.1111/j.1600-051X.2009.01472.x	McCracken GI, Heasman L, Stacey F, Swan M, Steen N, de Jager M, et al. The impact of powered and manual toothbrushing on incipient gingival recession. Journal of Clinical Periodontology 2009;36(11):950-7.	1 approved by the Newcastle and North Tyneside NHS Research Ethics Committee
2008	Moritis 2008		Moritis K, Jenkins W, Hefti A, Schmitt P, McGrady M. A randomized, parallel design study to evaluate the effects of a Sonicare and a manual toothbrush on plaque and gingivitis. Journal of Clinical Dentistry 2008;19(2):64-8.	--- (article has not been accessed online) ---
2008	Rosema 2008	doi.org/10.1902%2Fjop.2008.070654	Rosema NA, Timmerman MF, Versteeg PA, van Palenstein Helderman WH, Van der Velden U, Van der Weijden GA. Comparison of the use of different modes of mechanical oral hygiene in prevention of plaque and gingivitis. Journal of Periodontology 2008;79:1368-94.	2 approved by the Medical Ethics Committee of the Academic Medical Center (AMC) of Amsterdam (approval #: MEC 05/035 #05.17.0679).
2007	Biesbrock 2007	https://www.ncbi.nlm.nih.gov/pubmed/18277739	Biesbrock AR, Bartizek RD, Gerlach RW, Terezhalmy GT. Oral hygiene regimens, plaque control, and gingival health: a two-month clinical trial with antimicrobial agents. Journal of Clinical Dentistry 2007;18(4):101-5.	1 approved by an institutional review board
2007	Costa 2007	angle.org/doi/10.2319/0003-3219(2007)077[0361:EOUEA [illegible]	Costa MR, Silva VC, Miqui MN, Sakima T, Spolidorio DM, Cirelli JA. Efficacy of ultrasonic, electric and manual toothbrushes in patients with fixed orthodontic appliances. Angle Orthodontist 2007;77(22):361-6.	2 approved by the Ethics and Research Committee of the School of Dentistry (protocol number 03/03)
2007	Goyal 2007	https://www.ncbi.nlm.nih.gov/pubmed/17912998	Goyal CR, Qaqish JG, Galustians J, Ortblad K. Efficacy and safety of a new power toothbrush in a population with mild to moderate gingivitis. Journal of Clinical Dentistry 2007;18(3):65-9.	--- (article has not been accessed online) ---
2007	Gugerli 2007	doi.org/10.1902/jop.2007.060279	Gugerli P, Secci G, Mombelli A. Evaluation of the benefits of using a power toothbrush during the initial phase of periodontal therapy. Journal of Periodontology 2007; Vol. 78, issue 4:654-60.	1 approved by the Ethics Committee of the School of Dental Medicine, University of Geneva
2007	Moreira 2007	https://www.ncbi.nlm.nih.gov/pubmed/18277743	Moreira CH, Luz PB, Villarinho EA, Petri LC, Weidlich P, Rösing CK. A clinical trial testing the efficacy of an ionic toothbrush for reducing plaque and gingivitis. The Journal of Clinical Dentistry 2007;18(4):123-5.	--- (article has not been accessed online) ---

Cochrane_Author		Primary studies_	doi	
2005	Zimmer 2005	Zimmer S, Strauss J, Bizhang M, Krage T, Raab W H, Barthel C. Efficacy of the Cybersonic in comparison with the Braun 3D Excel and a manual toothbrush. Journal of Clinical Periodontology 2005; Vol. 32, issue 4:360-3.	doi: 10.1111/j.1600-051X.2005.00683.x.	1 approved by theethics committee of the Charite´Berlin,Germany
2004	McCracken 200	McCracken GI, Heasman L, Stacey F, Steen N, DeJager M, Heasman PA. A clinical comparison of an oscillating/rotating powered toothbrush and a manual toothbrush in patients with chronic periodontitis. Journal of Clinical Periodontology 2004;31(9):805-12.	doi.org/10.1111/j.1600-051X.2004.00559.x	1 approved by the Newcastle and North Tyneside NHS Research Ethics Committee
2004	Silverman 2004	Silverman J, Rosivack RG, Matheson PB, Houpt MI. Comparison of powered and manual toothbrushes for plaque removal by 4- to 5-year-old children. Pediatric Dentistry 2004;26:225-30.	ingentaconnect.com/contentone/aapd/pd/2004/00000026/00000003/art00005	1 approved by the University of Medicine and Dentistry of New Jersey Institutional Review Board.
2003	Lazarescu 2003	Lazarescu D, Boccaneala S, Illiescu A, De Boever JA. Efficacy of plaque removal and learning effect of a powered and a manual toothbrush. Journal of Clinical Periodontology 2003;30(8):726-31.	doi.org/10.1034/j.1600-051X.2003.00361.x	0 Ethics committee not mentioned
2002	Dentino 2002	Dentino AR, Derderian G, Wolf M, Cugini M, Johnson R, Van Swol RL, et al. Six-month comparison of powered versus manual toothbrushing for safety and efficacy in the absence of professional instruction in mechanical plaque control. Journal of Periodontology 2002;73(7):770-8.	doi.org/10.1902%2Fjop.2002.73.7.770	1 approved by the Marquette university human subjects institutional review board
2002	Hickman 2002	Hickman J, Millett DT, Sander L, Brown E, Love J. Powered vs manual tooth brushing in fixed appliance patients: a short term randomized clinical trial. The Angle Orthodontist 2002;72(2):135-40.	angle.org/doi/10.1043/0003-3219(2002)072<0135:PVMTBI>2.0.CO;2	1 approved by the Local Ethical Committee
2002	Lapiere unpubli	Lapiere A, Donck L, De Vree H, De Boever JA. Effectiveness of two electric toothbrushes as compared to a manual for plaque control in periodontitis patients with low compliance. Unpublished 2002.		--- (article has not been accessed online) ---
2002	Zimmer 2002	Zimmer S, Nezhat V, Bizhang M, Seemann R, Barthel CR. Clinical efficacy of a new sonic/ultrasonic toothbrush. Journal of Clinical Periodontolgy 2002;29(6):496-500.	doi.org/10.1034/j.1600-051X.2002.290604.x	0 Ethics committee not mentioned
2001	Garcia-Godoy 2	Garcia-Godoy F, Marcushamer M, Cugini M, Warren PR. The safety and efficacy of a children's power toothbrush and a manual toothbrush in 6-11 year-olds. American Journal of Dentistry 2001;14(4):195-9.	https://www.ncbi.nlm.nih.gov/pubmed/11699736	0 Ethics committee not mentioned

Cochrane_Author		Primary studies_	doi
2001	Haffajee 2001a	Haffajee AD, Thompson M, Torresyap G, Guerrero D, Socransky SS. Efficacy of manual and powered toothbrushes (I). Effect on clinical parameters. Journal of Clinical Periodontology 2001;28(10):937-46.	doi.org/10.1034/j.1600-051x.2001.028010937.x
		0 Ethics committee not mentioned	
2001	Warren 2001	Warren PR, Cugini M, Marks P, King DW. Safety, efficacy and acceptability of a new power toothbrush: a 3-month comparative clinical investigation. American Journal of Dentistry 2001;14(1):3-7.	https://www.ncbi.nlm.nih.gov/pubmed/11806476
		--- (article has not been accessed online) ---	
2000	Sharma 2000	Sharma N, Galustians HJ, Qaqish JG, Rustogi KN, Petrone ME, Volpe AR. Comparative efficacy on supragingival plaque and gingivitis of a manual toothbrush (Colgate Plus) and a battery-powered toothbrush (Colgate Actibrush) over a 30-day period. Compendium of Continuing Education Dental Supplement 2000;21(31):S9-13.	https://www.ncbi.nlm.nih.gov/pubmed/11908375
		--- (article has not been accessed online) ---	
2000	Singh unpublish	Singh A, Maddalozzo D, Geivelis M, Koerber A, Cornell W, Grys E. Efficacy of the Butler Pulse (TM) plaque remover in orthodontic patients. Journal of Dental Research 2000;79(IADR Abstracts):298 (Abstract No 1237)	
		--- (article has not been accessed online) ---	
2000	Soparkar 2000	Soparkar PM, Rustogi KN, Petrone ME, Volpe AR. Comparison of gingivitis and plaque efficacy of a battery-powered toothbrush and an ADA-provided manual toothbrush. Compendium of Continuing Education in Dentistry Supplement 2000;(31):S14-8.	https://www.ncbi.nlm.nih.gov/pubmed/11908370
		--- (article has not been accessed online) ---	
2000	Sowinski 2000	Sowinski JA, Battista GW, Petrone DM, Petrone ME, Rustogi KN, Chaknis P, et al. Comparative efficacy of Colgate Actibrush battery-powered toothbrush and Colgate Plus (manual) toothbrush on established plaque and gingivitis: a 30-day clinical study in New Jersey. Compendium of Continuing Education in Dentistry Supplement 2000;(31):S4-8.	https://www.ncbi.nlm.nih.gov/pubmed/11908374
		--- (article has not been accessed online) ---	
1999	Heasman 1999	Heasman PA, Stacey F, Heasman L, Sellers P, Macgregor ID, Kelly PJ. A comparative study of the Philips HP 735, Braun/Oral B D7 and the Oral B 35 Advantage toothbrushes. Journal of Clinical Periodontology 1999;26(2):85-90.	doi.org/10.1034/j.1600-051X.1999.260204.x
		1 approved by the Joint Ethics Committeeof the Newcastle and North Tyneside Health Authorities	
1999	Pucher 1999	Pucher JJ, Lamendola-Sitenga K, Ferguson D, Van Swoll R. The effectiveness of an ionic toothbrush in the removal of dental plaque and reduction on gingivitis in orthodontic patients. Journal of the Western Society of Periodontology/Periodontal Abstracts 1999;47(4):101-7.	
		--- (article has not been accessed online) ---	
1998	Clerehugh 1998	Clerehugh V, Williams P, Shaw WC, Worthington HV, Warren P. A practice-based randomised controlled trial of the efficacy of an electric and a manual toothbrush on gingival health in patients with fixed orthodontic appliances. Journal of Dentistry 1998;26(8):633-9.	doi.org/10.1016%2FS0300-5712%2897%2900065-1
		1 approved by the Dental Hospital/Central Manchester Health Authority Research Ethical Committee	

Cochrane_Author	Primary studies_	doi
1998 Cronin 1998 https://www.ncbi.nlm.nih.gov/pubmed/10530095	Cronin M, Dembling W, Warren PR, King DW. A 3-month clinical investigation comparing the safety and efficacy of a novel electric toothbrush (Braun Oral-B 3D Plaque Remover) with a manual toothbrush. American Journal of Dentistry 1998;11(Spec No):S17-21.	--- (article has not been accessed online) ---
1998 Forgas-B 1998 doi.org/10.1111/j.1600-051X.1998.tb02458.x	Forgas-Brockmann LB, Carter-Hanson C, Killoy WJ. The effects of an ultrasonic toothbrush on plaque accumulation and gingival inflammation. Journal of Clinical Periodontology 1998;25(5):375-9.	1 approved by the Institutional Re-view Board at the University of Missouri-Kansas City.
1997 Ainamo 1997 doi.org/10.1111/j.1600-051X.1997.tb01180.x	Ainamo J, Xie Q, Ainamo A, Kallio P. Assessment of the effect of an oscillating/rotating electric toothbrush on oral health. A 12-month longitudinal study. Journal of Clinical Periodontology 1997;24(1):28-33.	0 Ethics committee not mentioned
1997 Ho 1997	Ho HP, Niederman R. Effectiveness of the Sonicare sonic toothbrush on reduction of plaque, gingivitis, probing pocket depth and subgingival bacteria in adolescent orthodontic patients. Journal of Clinical Dentistry 1997;8(1 Spec No):15-9.	--- (article has not been accessed online) ---
1997 Yankell 1997	Yankell SL, Emling RC. A thirty-day safety and efficacy evaluation of the Rowenta, Braun and Sonicare powered toothbrushes and a manual toothbrush. Journal of Clinical Dentistry 1997;8:120-7.	--- (article has not been accessed online) ---
1996 Galgut 1996	Galgut PN. Efficacy of a new electronic toothbrush in removing bacterial dental plaque in young adults. General Dentistry 1996;44(5):441-5.	--- (article has not been accessed online) ---
1996 O'Beirne 1996 doi.org/10.1902%2Fjop.1996.67.9.900	O'Beirne G, Johnson RH, Persson GR, Spektor MD. Efficacy of a sonic toothbrush on inflammation and probing depth in adult periodontitis. Journal of Periodontology 1996;67(9):900-8.	1 approved by the the Human Subjects Review Committee.
1996 Stabholz 1996	Stabholz A, Babayof I, Mann J. The clinical effect of a newly designed electric toothbrush on supragingival plaque, gingivitis and gingival bleeding. Journal of Clinical Dentistry 1996;7(1):17-20.	--- (article has not been accessed online) ---
1996 Tritten 1996 doi.org/10.1111/j.1600-051X.1996.tb00588.x	Tritten CB, Armitage GC. Comparison of a sonic and a manual toothbrush for efficacy in supragingival plaque removal and reduction of gingivitis. Journal of Clinical Periodontology 1996;23(7):641-8.	--- (article has not been accessed online) ---

Cochrane_Author		Primary studies_	doi
1996	Van Swol 1996	Van Swol RL, Van Scotter DE, Pucher JJ, Dentino AR. Clinical evaluation of an ionic toothbrush in the removal of established plaque and reduction of gingivitis. Quintessence International 1996;27(6):389-94.	quintpub.com/journals/qi/fulltext.php?article_id=5759
		1 approved by the institutional review board at Marquette University	
1996	Yankell 1996	Yankell SL, Emling RC. A thirty-day evaluation of the Rowenta Dentiphant powered toothbrush in children for safety and efficacy. Journal of Clinical Dentistry 1996;7(4):96-100.	
		--- (article has not been accessed online) ---	
1995	Terezhalmy 199	Terezhalmy GT, Iffland H, Jelepis C, Waskowski J. Clinical evaluation of the effect of an ultrasonic toothbrush on plaque, gingivitis, and gingival bleeding: a six-month study. Journal of Prosthetic Dentistry 1995;73(1):97-103.	doi.org/10.1016%2FS0022-3913%2805%2980278-1
		0 Ethics committee not mentioned	
1994	Johnson 1994	Johnson BD, McInnes C. Clinical evaluation of the efficacy and safety of a new sonic toothbrush. Journal of Periodontology 1994;65(7):692-7.	doi.org/10.1902%2Fjop.1994.65.7.692
		1 approved by the University of Washington's Human Subjects Review Committee.	
1994	Stoltze 1994	Stoltze K, Bay L. Comparison of a manual and a new electric toothbrush for controlling plaque and gingivitis. Journal of Clinical Periodontology 1994;21(2):86-90.	doi.org/10.1111/j.1600-051X.1994.tb00284.x
		--- (article has not been accessed online) ---	
1994	van der Weijde	van der Weijden GA, Timmerman MF, Reijerse E, Danser MM, Mantel MS, Nijboer A, et al. The long-term effect of an oscillating/rotating electric toothbrush on gingivitis. An 8-month clinical study. Journal of Clinical Periodontology 1994;21(2):139-45.	doi.org/10.1111/j.1600-051X.1994.tb00292.x
		--- (article has not been accessed online) ---	
1993	Barnes 1993	Barnes CM, Weatherford TW 3rd, Menaker L. A comparison of the Braun Oral-B Plaque Remover (D5) electric and a manual toothbrush in affecting gingivitis. Journal of Clinical Dentistry 1993;4(2):48-51.	
		--- (article has not been accessed online) ---	
1993	Wilson 1993	Wilson S, Levine D, Dequincey G, Killoy WJ. Effects of two toothbrushes on plaque, gingivitis, gingival abrasion, and recession: a 1-year longitudinal study. Compendium Supplement 1993;(16):S569-79.	
		--- (article has not been accessed online) ---	
1993	Yukna 1993b	Yukna RA, Shaklee RL. Evaluation of a counter-rotational powered brush in patients in supportive periodontal therapy. Journal of Periodontology 1993;64(9):859-64.	doi.org/10.1902%2Fjop.1993.64.9.859
		1 approved by the University of Colorado Health Sciences Center Humans at Risk Committee	

Cochrane_Author		Primary studies_	doi
1992	Khocht 1992	Khocht A, Spindel L, Person P. A comparative clinical study of the safety and efficacy of three toothbrushes. Journal of Periodontology 1992;63(7):603-10.	
doi.org/10.1902%2Fjop.1992.63.7.603		0 Ethics committee not mentioned	
1991	Emling 1991	Emling RC, Raidl A, Greco MR, Shi X, Yankell SL. Clinical evaluation of the Plak Trac toothbrush. Journal of Clinical Dentistry 1991;2(3):57-62.	
		--- (article has not been accessed online) ---	
1989	Baab 1989	Baab DA, Johnson RH. The effect of a new electric toothbrush on supragingival plaque and gingivitis. Journal of Periodontology 1989;60(6):336-41.	
doi.org/10.1902%2Fjop.1989.60.6.336		1 approved by the University ofWashington's Human Subjects Review Committee.	
1989	Walsh 1989	Walsh M, Heckman B, Leggott P, Armitage G, Robertson PB. Comparison of manual and power toothbrushing, with and without adjunctive oral irrigation, for controlling plaque and gingivitis. Journal of Clinical Periodontology 1989;16(7):419-27.	
doi.org/10.1111/j.1600-051X.1989.tb01670.x		--- (article has not been accessed online) ---	
1966	Toto 1966	Toto PD, Goljan KR, Evans JA, Sawinski VJ. A study on the uninstructed use of an electric toothbrush. Journal of the American Dental Association 1966;72(4):904-5.	
doi.org/10.14219%2Fjada.archive.1966.0104		--- (article has not been accessed online) ---	
1965	Glass 1965	Glass RL. A clinical study of hand and electric toothbrushing. Journal of Periodontology 1965;36:322-7.	
doi.org/10.1902%2Fjop.1965.36.4.322		0 Ethics committee not mentioned	
1964	Lobene 1964a	Lobene RR. The effect of an automatic toothbrush on gingival health. Journal of Periodontology 1964;35:137-9.	
doi.org/10.1902%2Fjop.1964.35.2.137		0 Ethics committee not mentioned	
1964	Soparkar 1964	Soparkar PM, Quigley GA. Power versus hand brushing: effect on gingivitis. Journal of the American Dental Association 1964;68:182-7.	
doi.org/10.14219%2Fjada.archive.1964.0071		--- (article has not been accessed online) ---	
